# Trimethoxylated Halogenated Chalcones as Dual Inhibitors of MAO-B and BACE-1 for the Treatment of Neurodegenerative Disorders

**DOI:** 10.3390/pharmaceutics13060850

**Published:** 2021-06-08

**Authors:** Vishal Payyalot Koyiparambath, Jong Min Oh, Ahmed Khames, Mohamed A. Abdelgawad, Aathira Sujathan Nair, Lekshmi R. Nath, Nicola Gambacorta, Fulvio Ciriaco, Orazio Nicolotti, Hoon Kim, Bijo Mathew

**Affiliations:** 1 Department of Pharmaceutical Chemistry, AIMS Health Sciences Campus, Amrita School of Pharmacy, Amrita Vishwa Vidyapeetham, Kochi 682041, India; vishalpk11@gmail.com (V.P.K.); sujuaathira@gmail.com (A.S.N.); 2 Department of Pharmacy, Research Institute of Life Pharmaceutical Sciences, Sunchon National University, Suncheon 57922, Korea; ddazzo005@naver.com; 3 Department of Pharmaceutics and Industrial Pharmacy, College of Pharmacy, Taif University, P.O. Box-11099, Taif 21944, Saudi Arabia; dr_akhames@yahoo.com; 4 Department of Pharmaceutical Chemistry, College of Pharmacy, Jouf University, Sakaka 72341, Saudi Arabia; mhmdgwd@ju.edu.sa; 5 Department of Pharmaceutical Organic Chemistry, Faculty of Pharmacy, Beni-Suef University, Beni Suef 62514, Egypt; 6 Department of Pharmacogonosy, Amrita School of Pharmacy, Amrita Vishwa Vidyapeetham, AIMS Health Sciences Campus, Kochi 682041, India; lrnvaishnav@gmail.com; 7 Dipartimento di Farmacia—Scienze del Farmaco, Università degli Studi di Bari “Aldo Moro”, Via E. Orabona, 4, I-70125 Bari, Italy; nicola.gambacorta1@uniba.it (N.G.); orazio.nicolotti@uniba.it (O.N.); 8 Dipartimento di Chimica, Università degli Studi di Bari “Aldo Moro”, Via E. Orabona, 4, I-70125 Bari, Italy; fulvio.ciriaco@uniba.it

**Keywords:** monoamine oxidase, β-secretase, chalcones, reversibility, cytotoxicity, molecular docking

## Abstract

Six halogenated trimethoxy chalcone derivatives (CH1–CH6) were synthesized and spectrally characterized. The compounds were further evaluated for their inhibitory potential against monoamine oxidases (MAOs) and β-secretase (BACE-1). Six compounds inhibited MAO-B more effectively than MAO-A, and the 2′,3′,4′-methoxy moiety in CH4–CH6 was more effective for MAO-B inhibition than the 2′,4′,6′-methoxy moiety in CH1–CH3. Compound CH5 most potently inhibited MAO-B, with an IC_50_ value of 0.46 µM, followed by CH4 (IC_50_ = 0.84 µM). In 2′,3′,4′-methoxy derivatives (CH4-CH6), the order of inhibition was –Br in CH5 > -Cl in CH4 > -F in CH6 at the *para*-position in ring B of chalcone. CH4 and CH5 were selective for MAO-B, with selectivity index (SI) values of 15.1 and 31.3, respectively, over MAO-A. CH4 and CH5 moderately inhibited BACE-1 with IC_50_ values of 13.6 and 19.8 µM, respectively. When CH4 and CH5 were assessed for their cell viability studies on the normal African Green Monkey kidney cell line (VERO) using MTT assays, it was noted that both compounds were found to be safe, and only a slightly toxic effect was observed in concentrations above 200 µg/mL. CH4 and CH5 decreased reactive oxygen species (ROS) levels of VERO cells treated with H_2_O_2_, indicating both compounds retained protective effects on the cells by antioxidant activities. All compounds showed high blood brain barrier permeabilities analyzed by a parallel artificial membrane permeability assay (PAMPA). Molecular docking and ADME prediction of the lead compounds provided more insights into the rationale behind the binding and the CNS drug likeness. From non-test mutagenicity and cardiotoxicity studies, CH4 and CH5 were non-mutagenic and non-/weak-cardiotoxic. These results suggest that CH4 and CH5 could be considered candidates for the cure of neurological dysfunctions.

## 1. Introduction

A well-known aspect of monoamine oxidases (MAOs) is their role in metabolizing various types of biogenic amines, which can impart a crucial role in modulating the central nervous system (CNS) neurotransmitter functions [1]. The malfunctioning of these neurotransmitters by the oxidative deamination process by MAOs leads to a variety of neurodegenerative disorders (NDDs) such as Alzheimer’s disease (AD) and Parkinson’s disease (PD) [2,3,4]. Among the MAO family, selective inhibitors of MAO-A can be employed for managing various depressive states, and at the same time, inhibitors of MAO-B can be widely used for the adjuvant therapy for NDDs [5,6]

Subsequently, MAOs have been extensively researched, with over 23,000 publications dealing with their properties, enzymology, functions, and specific inhibitors from various classes, according to the PubMed database [7]. Monoamine oxidase was chosen as a target here since it is a common enzyme for various neurological disorders and has the benefit in kinetics studies of allowing us to determine how quickly enzymes will function in tissues with the substrate concentrations they are likely to encounter, as well as how different substrates will fight for the enzyme [8]. The life of two isoforms, MAO-A and MAO-B, each with distinct structural features, has inspired clinical strategies to develop isoform-tailored inhibitors that might be used to treat a wide range of depressive diseases and CNS-related disorders [9,10].

β-secretase (BACE-1) is a crucial cleaving enzyme that takes part in the degradation of the amyloid precursor protein (APP) [11]. This cleavage process leads to the formation of amyloid β (Aβ) protein, which is neurotoxic and can trigger the neurodegeneration and plaque deposition in the AD brain [12]. Numerous studies have documented that inhibitors of BACE-1 have a prominent role in diminishing the Aβ concentration in the brain and thwarting the progression of AD [13]. Considering the benefits of a multitarget directed ligand (MTDL) approach by inhibiting MAO-B and Aβ production would be of great interest in AD therapy. Very limited literature has been reported regarding the development of dual-acting MAO-B and BACE-1 inhibitors [14,15].

Chalcones are well-known structural motifs with α,β-unsaturated ketones and are open-chain flavonoids [16]. The presence of three rotatable bonds and the Michael acceptor nature of the linker present in the chalcone scaffold provided a remarkable ability to reach different enzyme targets [17]. Thus, many pharmacological active profiles involve chalcone-containing compounds, including anti-tumor, anti-diabetic, anti-inflammatory, and anti-AD agents [18,19,20,21,22]. Based upon the wealth of information, halogens have now established themselves as a central player in supramolecular chemistry with wide applications in medicinal chemistry [23]. The electron-accepting nature of halogens can preferably promote more non-covalent hydrophobic interactions in several enzyme targets [24]. A recent report documented that the presence of halogens in various classes of MAO inhibitors resulted in improved MAO-B selectivity by the stabilizing property of halogen bonding in the inhibitor-binding cavity (IBC) of the enzyme [25].

Based on the previous studies, in the simple framework of non-nitrogenous based chalcones, the selective and potent candidates against the MAO-B isoform were characterized by the presence of methoxyl or methyl substituents at the *para* or *ortho* positions of phenyl ring **A [26,27,28,29,30,31]**. At the same time, the presence of halogens and electron-releasing substituents, preferably methoxy, dimethylamino, ethyl acetohydroxamate, and ethoxy on the *para* position of phenyl ring **B**, are well tolerated in the MAO-B inhibition [32,33,34,35,36,37,38,39,40,41,42,43,44,45,46,47,48,49,50]. Based upon the existing literature, it is clearly proved that 99% of chalcone derived compounds are a selective and reversible type of MAO-B inhibitors [51,52,53]. In this scenario, our attention is mainly focused on the development of two series by the incorporation of three methoxy pharmacophores on the **A** ring of the phenyl ring of chalcones at the positions of 2′, 4′, and 6′ and 2′, 3′, and 4′, respectively. A variety of halogens such as chlorine, bromine, and fluorine have been also introduced on the *para* position on the **B** ring phenyl unit. To the best of our knowledge, such a design strategy has not been reported regarding the effect and orientation of various halogens and trimethoxyl groups on chalcones framework for the development of multi-targeted MAO-B/BACE-1 inhibitors.

The present study emphasizes the synthesis of 2′, 4′, and 6′ and 2′, 3′, and 4′ trimethoxylated halogenated chalcones and the evaluation of their MAO-A, MAO-B, and BACE-1 inhibition. The lead molecules were further studied in kinetics, reversibility, cytotoxicity, and reactive oxygen species (ROS) assays. A molecular docking study established the detailed protein–ligand binding interactions of the lead molecules in their respective active sites of enzyme targets.

## 2. Materials and Methods

### 2.1. Synthesis

**Scheme 1:**
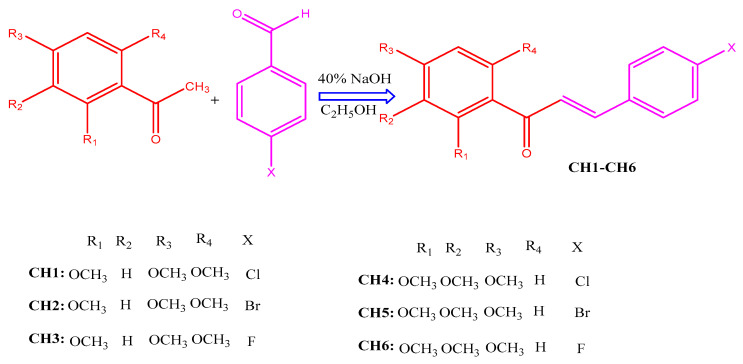
Synthetic route for (CH1–CH6) series.

A mixture of trimethoxy acetophenone (0.01 mol), *para*-substituted halogenated benzaldehyde (0.01 mol), and 50% KOH was added to 15 mL of methanol and was kept stirring at room temperature for 15 h [54]. The resulting solution was then filtered, washed with water, and dried. The product was then recrystallized from methanol-yielded pure crystals. The synthetic route for the titled derivatives is depicted in Scheme 1.

#### 2.1.1. (E)-3-(4-chlorophenyl)-1-(2,4,6-trimethoxyphenyl)prop-2-en-1-one (CH1)

M.P: 136–138 °C; ^1^H NMR (500 MHz, CDCl_3_) δ: 3.77 (s, 6H, Ar-2′OCH_3,_ Ar-6′OCH_3,_), 3.86 (s, 3H, Ar-4′OCH_3_), 6.15 (s, 2H, Ar-3′CH_,_ Ar-5′CH), 6.94–6.91 (1H, d, *J* = 15.0 Hz, -CH_α_), 7.33–7.30 (1H, d, *J* = 15.0 Hz, -CH_β_), 7.34 (2H, d, Ar-3CH, Ar-5CH), 7.46 (2H, d, Ar-2H, Ar-6H). ^13^C-NMR (500 MHz, CDCl_3_) δ: 193.91, 162.57, 158.93, 142.39, 136.01, 133.55, 129.54, 129.39, 129.09, 111.60, 90.69, 55.94. Molecular formula C_18_H_17_ClO_4_ (HRMS) Calculated = 332.7781, Observed = 333.0892.

#### 2.1.2. (E)-3-(4-bromophenyl)-1-(2,4,6-trimethoxyphenyl)prop-2-en-1-one (CH2)

M.P: 120–122 °C; ^1^H NMR (500 MHz, CDCl_3_) δ: 3.77 (s, 6H, Ar-2′OCH_3,_ Ar-6′OCH_3,_), 3.85 (s, 3H, Ar-4′OCH_3_), 6.15 (s, 2H, Ar-3′CH_,_ Ar-5′CH), 6.96–6.93 (1H, d, *J* = 15.0 Hz, -CH_α_), 7.29–7.26 (1H, d, *J* = 15.0 Hz, -CH_β_), 7.39–7.37 (2H, d, Ar-3CH, Ar-5CH), 7.50–7.48 (2H, d, Ar-2H, Ar-6H). ^13^C-NMR (500 MHz, CDCl_3_) δ: 193.84, 162.59, 158.95, 142.38, 134.00, 132.04, 129.75, 129.49, 124.36, 111.62, 90.71, 55.95. Molecular formula C_18_H_17_BrO_4_ (HRMS) Calculated = 377.2291, Observed = 377.0370.

#### 2.1.3. (E)-3-(4-fluorophenyl)-1-(2,4,6-trimethoxyphenyl)prop-2-en-1-one (CH3)

M.P: 110–112 °C; ^1^H NMR (500 MHz, CDCl_3_) δ: 3.76–3.71 (s, 6H, Ar-2′OCH_3,_ Ar-6′OCH_3,_), 3.86–3.82 (s, 3H, Ar-4′OCH_3_), 6.16 (s, 2H, Ar-3′CH_,_ Ar-5′CH), 6.90–6.93 (1H, d, *J* = 15.0 Hz, -CH_α_), 7.35–7.32 (1H, d, *J* = 15.0 Hz, -CH_β_), 7.03–7.07 (2H, d, Ar-3CH, Ar-5CH), 7.49–7.52 (2H, d, Ar-2H, Ar-6H). ^13^C-NMR (500 MHz, CDCl_3_) δ: 194.04, 164.85, 161.40, 158.74, 144.32, 142.72, 131.28, 130.12, 127.00, 115.87, 90.71, 55.49. Molecular formula C_18_H_17_FO_4_ (HRMS) Calculated = 316.3235, Observed = 317.1219.

#### 2.1.4. (E)-3-(4-chlorophenyl)-1-(2,3,4-trimethoxyphenyl)prop-2-en-1-one (CH4)

M.P: 108–110 °C; ^1^H NMR (500 MHz, CDCl_3_) δ: 3.92 (s, 9H, Ar-2′OCH_3,_ Ar-3′OCH_3,_ Ar-4′OCH_3_), 6.76 (d, Ar-5′CH), 7.38–7.36 (d,2H, Ar-3H, Ar-5H), 7.55–7.53 (d,2H, Ar-2H, Ar-6H), 7.51 (Ar-6′CH), 7.50–7.47 (1H, d, *J* = 15.0 Hz, -CH_α_), 7.65–7.62 (1H, d, *J* = 15.0 Hz, -CH_β_). ^13^C-NMR (500 MHz, CDCl_3_) δ: 190.56, 157.26, 153.87, 142.14, 141.37, 136.04, 133.72, 129.53, 129.18, 127.00, 126.56, 125.97, 107.41, 62.17, 61.12, 56.16. Molecular formula C_18_H_17_ClO_4_ (HRMS) Calculated = 332.7781, Observed = 333.0892. Molecular formula C_18_H_17_ClO_4_ (HRMS) Calculated = 332.7781, Observed = 333.0892.

#### 2.1.5. (E)-3-(4-bromophenyl)-1-(2,3,4-trimethoxyphenyl)prop-2-en-1-one (CH5)

M.P: 98–100 °C; ^1^H NMR (500 MHz, CDCl_3_) δ: 3.92 (s, 9H, Ar-2′OCH_3,_ Ar-3′OCH_3,_ Ar-4′OCH_3_), 6.75 (d, 1H, Ar-5′CH), 7.38–7.36 (d,2H, Ar-3H, Ar-5H), 7.55–7.53 (d,2H, Ar-2H, Ar-6H), 7.51–7.47 (1H, Ar-6′CH), 7.51–7.48 (1H, d, *J* = 15.0 Hz, -CH_α_), 7.65–7.62 (1H, d, *J* = 15.0 Hz, -CH_β_) ^13^C-NMR (500 MHz, CDCl_3_) δ: 190.50, 157.25, 153.87, 142.14, 141.34, 136.04, 133.71, 129.53, 129.19, 126.99, 126.56, 125.97, 107.41, 62.17, 61.12, 56.17. Molecular formula C_18_H_17_BrO_4_ (HRMS) Calculated = 377.2291, Observed = 377.0370.

#### 2.1.6. (E)-3-(4-fluorophenyl)-1-(2,3,4-trimethoxyphenyl)prop-2-en-1-one (CH6)

M.P: 80–82 °C; ^1^H NMR (500 MHz, CDCl_3_) δ: 3.92 (s, 9H, Ar-2′OCH_3,_ Ar-3′OCH_3,_ Ar-4′OCH_3_), 6.75 (d, Ar-5′CH), 7.11–7.07 (d,2H, Ar-3H, Ar-5H), 7.62–7.59 (d,2H, Ar-2H, Ar-6H), 7.50 (Ar-6′CH), 7.45–7.42 (1H, d, *J* = 15.0 Hz, -CH_α_), 7.67–7.64 (1H, d, *J* = 15.0 Hz, -CH_β_). ^13^C-NMR (500 MHz, CDCl_3_) δ: 190.63, 157.15, 153.81, 142.14, 141.67, 131.44, 131.42, 130.28, 130.21, 126.66, 126.29, 125.91, 107.41, 62.17, 61.12, 56.17. Molecular formula C_18_H_17_FO_4_ (HRMS) Calculated = 316.3235, Observed = 317.1219.

### 2.2. MAOs and BACE1 Inhibition Studies

MAO events were assessed with recombinant MAO-A and MAO-B, as well as the substrates kynuramine (0.06 mM) and benzylamine (0.3 mM). The kinetics of MAO-B were analyzed under 2.4 × K_m_ (i.e., 0.051 mM) of benzylamine. BACE-1 was measured using a β-secretase (BACE-1) assay kit at 320 nm for excitation and 405 nm for emission using a fluorescence spectrometer. The inhibitory activities of CH1-CH6 against MAO-A, MAO-B and BACE-1 were observed at 10 µM. IC_50_ values of CH4 and CH5, which showed residual activities of <50% for MAO-A and MAO-B, were evaluated, as described previously. The IC_50_ value of CH5 for BACE-1 was also analyzed [55,56].

### 2.3. Enzyme Inhibition and Kinetic Studies

The inhibitory actions of the six molecules against MAO-A, MAO-B, and BACE-1 were first tested at 10 µM. Initially, the IC_50_ values of the compounds for MAOs were calculated [57]. At three inhibitory concentrations and five substrate concentrations, kinetic tests were conducted on CH4 and CH5, which inhibited MAO-B most potently.

### 2.4. Inhibitor Reversibility Analysis

Dialysis was used to assess the reversibility of MAO-B inhibitions by CH4 and CH5 after preincubating them for 30 min at 0.15 µM with MAO-B. For contrast, MAO-B was preincubated with lazabemide (a reference reversible MAO-B inhibitor) or pargyline (a reference irreversible MAO-B inhibitor) at 0.20 and 0.30 µM. Reversibility patterns were analyzed by comparing the activities of dialyzed (A_D_) and undialyzed (A_U_) samples [58].

### 2.5. Cytotoxicity and ROS Assay

The lead molecules CH4 and CH5 in the current series were further evaluated for their cytotoxic nature and ROS scavenging ability by the previously reported procedure [59,60] (Supplementary Material).

### 2.6. Blood-Brain Barrier (BBB) Permeability

The CNS bioavailability of the molecules was confirmed by a parallel artificial membrane permeability assay (PAMPA) by Di et al. [61].

### 2.7. Computational Studies

X-ray coordinates of the crystal structures of both the MAO isoforms and BACE-1 were retrieved from the Protein Data Bank with the entries 2Z5X, 2V5Z and 3TPP, respectively [62,63,64]. Protein and ligand structures were treated with the Protein Preparation Wizard and Ligprep Tool, respectively [65,66,67]. These preliminary steps are crucial for further docking analyses, allowing the generation of the correct protonation states at physiological pH or the generation of all allowed tautomers and possible conformations. Enclosing boxes were centered on the center of a mass of cognate ligand, and SP docking protocols were employed using GLIDE software [68]. To corroborate the reliability of docking simulations, redocking analyses were performed on the cognate ligands in their binding sites. Cognate ligands were moved back to the original positions with Root Mean Square Deviations (RMSD), considering all the heavy atoms, equal to 0.155 Å, 0.208 Å, and 0.467 Å for BACE-1, MAO-A, and MAO-B cognate ligands, respectively. Discovery Studio 2.5 software package (Accelrys, San Diego, CA, USA) was used to predict the ADMET parameters of all the molecules. The Vega and PredHerg platforms were employed to assess the mutagenicity and cardiotoxicity potentials [69,70].

## 3. Results

### 3.1. Synthesis

The synthesis of two small series of trimethoxylated halogenated chalcones was achieved by the Claisen–Schmidt condensation of 2,4,6-trimethoxy acetophenone/2,3,4-trimethoxy acetophenone with various *para*-substituted halogenated benzaldehydes. ^1^H NMR showed a sharp doublet peak for Hα and Hβ at 7.65–7.62 and 7.50–7.47, respectively. A large coupling constant of 15Hz of the double bond of chalcones revealed its *trans* conformation [71]. The protons of methoxy groups showed a singlet peak at 3.92, while the ^13^C NMR showed a peak at 55.95–62.17 for the methoxy carbon and a characteristic peak of carbonyl carbon at 190.63–193.91. The HRMS analysis showed the molecular weight of the targeted compounds [Supplementary Material].

### 3.2. MAO-A, MAO-B, and BACE1 Inhibition Studies

The six compounds CH1-CH6 inhibited MAO-B more effectively than MAO-A, and CH4 and CH5 revealed the most inhibitory activities against MAO-A and MAO-B at 10 µM with residual activities of <50% (Table 1). Compound CH5 most potently inhibited MAO-A with an IC_50_ value of 0.46 µM, followed by CH4 (IC_50_ = 0.84 µM). In the compounds, 2′,3′,4′-methoxy moiety in CH4-CH6 was more effective for MAO-B inhibition than 2′,4′,6′-methoxy moiety in CH1-CH3. In 2′,3′,4′-methoxy derivatives (CH4-CH6), inhibitory activities against MAO-B increased in the order of –Br in CH5 > –Cl in CH4 > –F in CH6 at *para*-position in ring B. CH5 and CH4 were selective for MAO-B with selectivity index (SI) values of 31.3 and 15.1, respectively, over MAO-A. No compounds effectively inhibited BACE-1, except CH4 and CH5, which moderately inhibited BACE-1 with IC_50_ values of 13.6 and 19.8 µM, respectively (Table 1). The IC_50_ value of **CH4** for BACE-1 (13.6 µM) was similar to that of reference quercetin (IC_50_ = 13.4 µM)

Based upon the above small selected library of compounds, it is possible to expand some interesting structure–activity relationships (SARs) on the basis of the orientation pattern of methoxyl groups on the various positions of phenyl ring **A** and the presence of various halogens on the *para*-position of ring **B** of chalcones. The results clearly documented that all the trimethoxylated halogenated derivatives showed moderate to good inhibition of MAO-B as compared to MAO-A. Considering the orientation pattern, it is clearly understood that introduction of methoxyl groups on the 2′, 4′, and 6′ positions does not have much influence when compared with the positions of 2′, 3′, and 4′. This suggests that the crowding of methoxyl groups without the separation of a carbon atom on the **A** ring is crucial for the activity. As regards halogen substitution on the **B** ring, bromine or chlorine improved MAO-B inhibitory activity when compared to fluorine substitution (the residual activities were 0.93% and 0.38% at 10.0 μM, respectively).

The two representative MAO-B inhibitors, **CH4** and **CH5,** showed good BACE-1 inhibition with 54.3% and 64.3% residual activity, respectively, at 10.0 μM. The IC_50_ value of the BACE-1 inhibition of **CH4** was 13.6 μM, which, interestingly, is very close to the standard value. In BACE-1 inhibition, both chlorine and bromine have a large impact on the fluorine substitution

### 3.3. Kinetic Study

**Figure 1:**
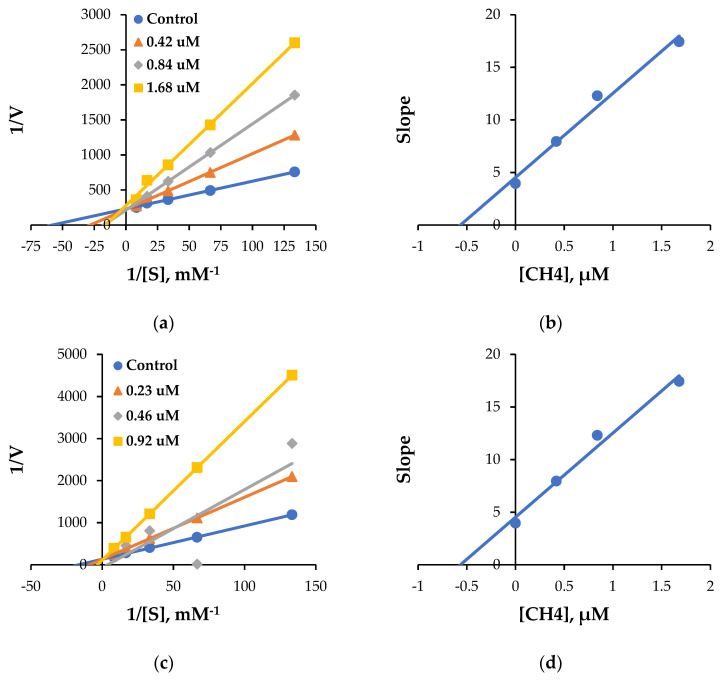
Lineweaver–Burk plots for MAO-B inhibition by CH4 and CH5 (**a**,**c**), and their respective secondary plots (**b**,**d**) of the slopes vs. inhibitor concentrations.

Kinetic experiments were carried out at five concentrations of the substrates and three inhibitor concentrations. In the kinetic studies of MAO-B binding by CH4 and CH5, Lineweaver–Burk plots showed that CH4 and CH5 were competitive inhibitors of MAO-B (Figure 1a,c), and their secondary plots showed that their K_i_ values were 0.68 ± 0.17 and 0.31 ± 0.014 µM, respectively (Figure 1b,d). These results suggest that CH4 and CH5 compete with the substrate at the active site of MAO-B.

### 3.4. Reversibility Studies

**Figure 2:**
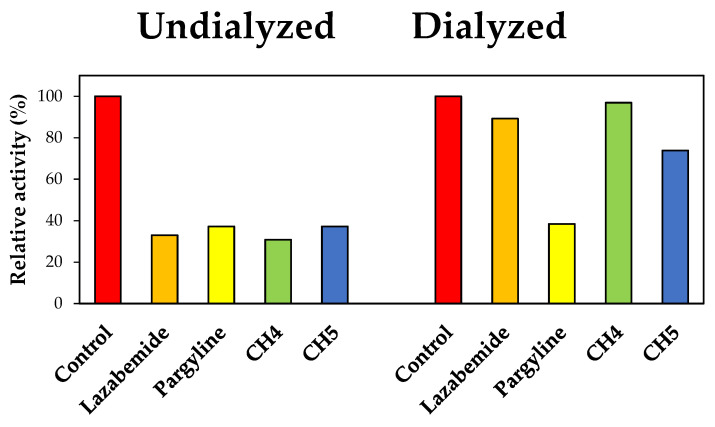
Recoveries of MAO-B inhibitions by CH4 and CH5 using dialysis experiments.

The reversibilities of the inhibitions were analyzed using dialysis after preincubating MAO-B with CH4 or CH5 for 30 min, as previously described. In the experiments, the concentrations used were the following: CH4 at 1.68 µM, CH5 at 0.92 µM, lazabemide (a reference reversible inhibitor) at 0.22 µM, and pargyline (a reference irreversible inhibitor) at 0.28 µM. The relative activities for undialyzed (A_U_) and dialyzed (A_D_) samples were compared to determine the reversibility patterns. In reversibility experiments using dialysis, the inhibition of MAO-B by CH4 and CH5 was recovered from 30.9% (A_U_) to 96.9% (A_D_), and from 37.2% to 73.8%, respectively (Figure 2). The recovery values were indistinguishable to those of the reversible reference lazabemide (from 33.0% to 89.2%), contrary to the inhibition of MAO-B by the irreversible inhibitor pargyline, which was not recovered (i.e., from 37.2% to 38.5%). These experiments indicated that CH4 and CH5 were reversible inhibitors of MAO-B.

### 3.5. Blood–Brain Barrier (BBB) Permeation Studies

From the assay, highly effective permeabilities and high CNS bioavailabilities were observed for the halogenated chalcones with Pe ranges of 13.22~15.65 × 10^−6^ cm/s (Table 2). All halogenated substituted derivatives showed higher CNS permeabilities than unsubstituted ones.

### 3.6. In Vitro Toxicity Evaluation

**Figure 3:**
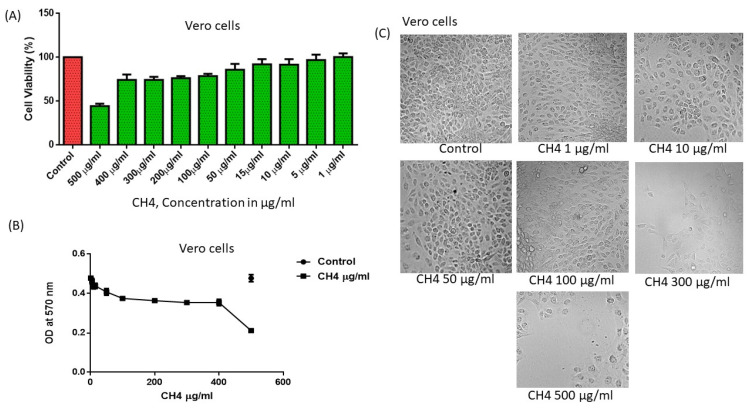
Evaluation of CH4 effect on cell viability in Vero cell line. (**A**) Cell viability; a very high percentage of viable cells (>90%) up to 200 µg/mL. (**B**) Representation of a dose–response curve and IC_50_; 198.5 µg/mL according to GraphPad Prism 6.0 software (USA). (**C**) Morphological observation of Vero cells in treatment with different concentrations under phase-contrast microscope. Vero cells were incubated with CH4 for 48 h, and cell viability was calculated by MTT assay as described in “Experimental Procedures.” Untreated cells (control) were considered to have 100% viability.

**Figure 4:**
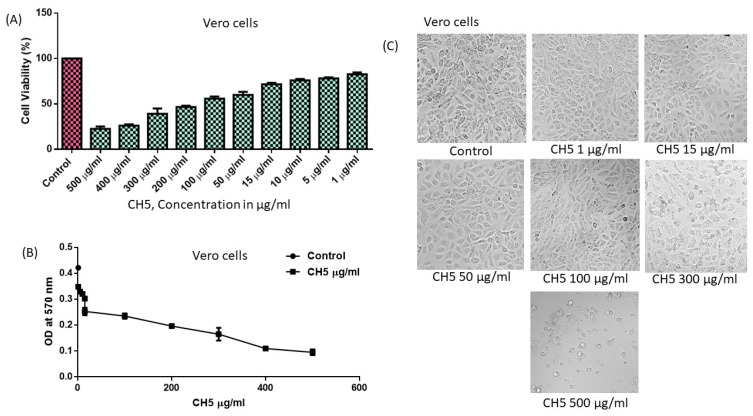
Evaluation of CH5 effect on cell viability in Vero cell line. (**A**) Cell viability; a very high percentage of viable cells (>90%) up to 100 times the effective concentration. (**B**) Representation of a dose–response curve and IC_50_; 126.4 µg/mL. (**C**) Morphological observation of Vero cells on treatment with different concentration under phase-contrast microscope. Vero cells were incubated with CH5 for 48 h, and cell viability was calculated by MTT assay as described in “Experimental Procedures.” Untreated cells (control) were considered to have 100% viability.

An in vitro toxicity evaluation of CH4 and CH5 was carried out using an MTT assay. We used the African Green Monkey kidney cell line (Vero) as a valuable in vitro model for basal toxicity studies [72]. To check the toxicity of the tested compounds, the Vero cell line was incubated with different concentrations of CH4 and CH5 (1 to 500 µg/mL), and the relative cell viability was measured after 48 h. Both the compounds induced toxicity at concentrations higher than 200 µg/mL (Figure 3A and Figure 4A). The result of the cell viability assay was represented as a dose–response curve using the GraphPad Prism 6.0 software (USA). The IC_50_ value was calculated at a 198.5 µg/mL for CH4 (Figure 3B) and 126.4 µg/mL for CH5 (Figure 4B). The reduction in cell number and also the membrane damage were very evident in treatment with CH4 and CH5 at IC_50_ and higher concentrations, as analyzed by phase-contrast microscope. These two experiments strongly support that CH4 and CH5 are one hundred times safer than the effective concentration of one micromolar (Figure 3C and Figure 4C). Both results demonstrate that the undesired changes in membrane integrity and a reduction in cell number are indicative of oxidative stress only at higher concentrations.

### 3.7. ROS Assay

**Figure 5:**
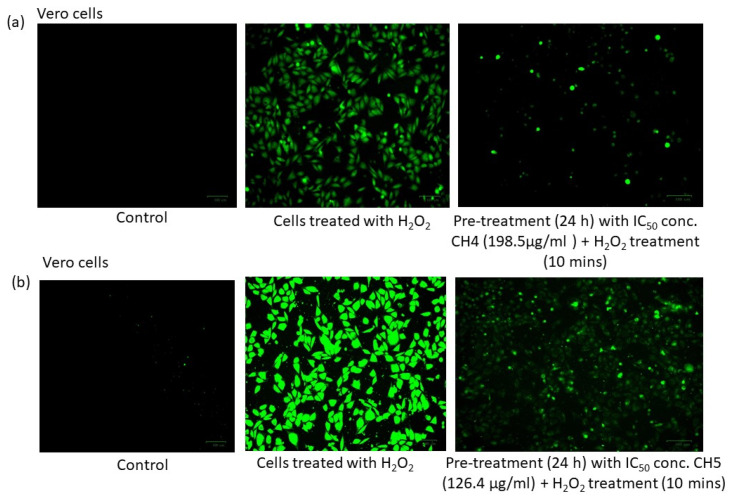
Effects of CH4 (**a**) and CH5 (**b**) on Vero cells treated with H_2_O_2_ to induce ROS. Vero cells were treated with 100 µg/mL of 30% H_2_O_2_, and the production of ROS was evaluated immediately as described in “Experimental Procedures”.

Our next attempt was to study the effect of CH4 and CH5 on inhibiting the excess reactive oxygen species (ROS) involved in the pathogenesis of neurodegenerative diseases. This ROS causes neuronal damage via oxidative damage in the brain during neurodegenerative diseases [73,74]. We examined the intracellular ROS generation on Vero cell cultures after exposure to exogenous H_2_O_2_. During our experiment, it was noted H_2_O_2_-treated Vero cells had a higher ROS generation. When we incubated Vero cells with CH4 and CH5 at IC_50_, we observed a decrease in ROS levels compared with non-treated cells (Figure 5a,b). Based on this observation, it is understood that the disruption of the balance between pro-oxidant and antioxidant levels can be retained by CH4 and CH5 at IC_50_ concentrations.

### 3.8. Computational Studies

**Figure 6:**
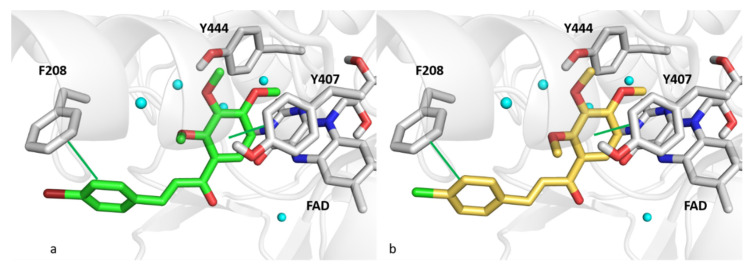
Zoomed in view of an MAO-A binding pocket. Panels (**a**,**b**) reported the best pose returned from docking analysis for CH5 (green sticks) and CH4 (yellow sticks), respectively. Cyan spheres indicate water molecules. Green lines show Pi–Pi contacts.

**Figure 7:**
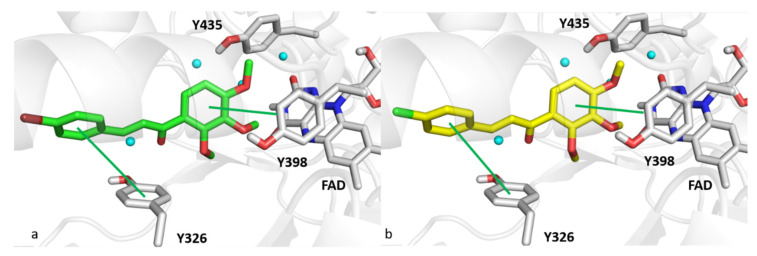
Zoomed in view of an MAO-B binding pocket. Panels (**a**,**b**) reported the best pose returned from docking analysis for CH5 (green sticks) and CH4 (yellow sticks), respectively. Cyan spheres indicate water molecules. Green lines show Pi–Pi contacts.

**Figure 8:**
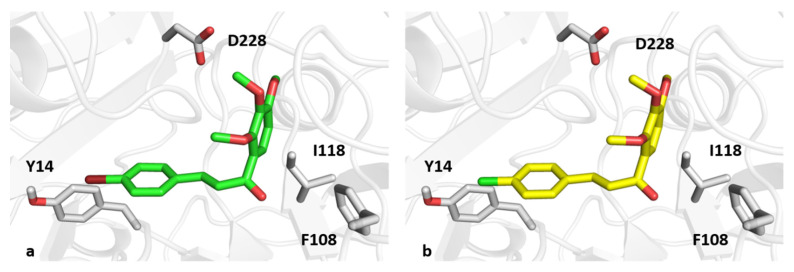
Zoomed in view of a BACE-1 binding pocket. Panels (**a**,**b**) reported the best pose returned from docking analysis for CH5 (green sticks) and CH4 (yellow sticks), respectively.

Compounds CH5 and CH4 can bind the MAO-A binding pocket with similar posing (Figure 6). The *para*-bromine and *para*-fluorine styrene can establish Pi–Pi interactions with F208, and tri-methoxy phenyl rings can cause Pi–Pi interaction with Y407, facing FAD. Docking score values were equal to −6.416 kcal/mol and −6.465 kcal/mol for CH5 and CH4, respectively. As far as MAO-B is concerned, the *para*-bromine and *para*-fluorine styrene can interact with the MAO-B-selective residue Y326, and trimethoxy phenyl rings face the FAD cofactor, forming Pi–Pi interactions with the side chain of the Y398 aromatic residue (Figure 7). Docking score values were equal to −9.824 kcal/mol and −10.251 kcal/mol for CH5 and CH4, respectively. As far as BACE-1 is concerned, compounds CH5 and CH4 can establish mainly hydrophobic interactions. In particular, the tri-methoxy aromatic rings can face a hydrophobic region made by side chains of F108 and I118, and the halogenated styrene moieties take place towards Y14 side chain (Figure 8). Docking score values were equal to −6.749 kcal/mol and −6.762 kcal/mol for CH5 and CH4, respectively.

Computational analyses were performed in order to shed light on the interactions behind the higher affinity towards MAO-B. The interaction between CH5 and CH4 with Y326 MAO-B selective residue is crucial for enhancing activity and selectivity towards MAO-B. Furthermore, despite the binding poses of the two compounds being similar between MAO binding pockets, the gap between the docking score values towards the two receptors significantly favors MAO-B. For this reason, a more detailed analysis of the terms that constitute the GLIDE scoring function was performed, revealing that the Glide energy term values, which are a sum of van der Waals and Coulomb energy terms, were equal to −50.263 kcal/mol and −46.128 kcal/mol for CH4 and CH5 bonded to MAO-B, respectively, and −21.341 kcal/mol and −23.449 kcal/mol for CH5 and CH4 bonded to MAO-A, respectively, thus considerably favoring the binding to MAO-B. Regarding computational simulations of BACE-1, the binding was mainly due to hydrophobic interactions and the Glide energy term values were equal to −46.583 kcal/mol and −45.809 kcal/mol, thus comparable to those of MAO-B.

**Figure 9:**
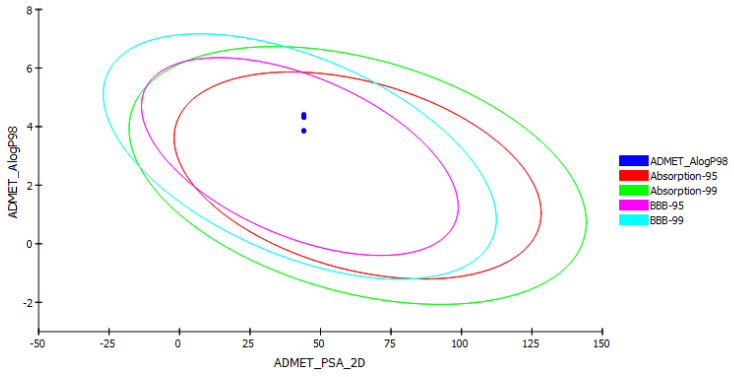
Predicted pharmacokinetic profile of **CH** series.

On the basis of ADME prediction, all the compounds in the present study showed good absorption and BBB permeability, at 99% and 95%, respectively (Figure 9). A drug target matching analysis was also performed by using our predictive platform MuSSel (Multifingerprint Similarity Search aLgorithm) [75,76]. It is notable that MAO-A and MAO-B were predicted as reliable targets for CH4 and CH5 [Supplementary Material].

Finally, we predicted the mutagenicity and cardiotoxicity potentials for CH4 and CH5 [77]. These are two relevant toxicity endpoints of the utmost importance to prioritize compounds in early stages of drug discovery and are often behind the attrition of preclinical and clinical studies [78,79]. Regarding the mutagenicity, four non-test predictive models (i.e., CAESAR model version 2.1.13; SARpy/IRFMN model version 1.0.7; ISS model version 1.0.2; KNN/read-across model version 1.0.0) were employed *in consensus* to provide a qualitative assessment of the mutagenic potentials of CH4 and CH5 [80]. Interestingly, both the compounds were found to be non-mutagenic [Supplementary Materials]. Predictive QSAR models of hERG blockage were employed for cardiotoxicity analysis [81]. In the results, CH4 was returned to be non-cardiotoxic, while CH5 was flagged as potentially weak cardiotoxic (Supplementary Materials).

## 4. Conclusions

In summary, two sets of trimethoxylated halogenated chalcones were synthesized, characterized, and evaluated for their MAO and BACE-1 inhibitory activities. CH4 and CH5 were revealed as competitive and reversible inhibitors of MAO-B, with K_i_ values of 0.68 ± 0.17 and 0.31 ± 0.014 µM, respectively. On the other hand, CH4 and CH5 showed good BACE-1 inhibition with IC_50_ values of 13.6 and 19.8 μM, respectively. Additionally, CH4 and CH5 were found to be safe as analyzed by an in-vitro toxicity study. Additionally, the pro-oxidant and antioxidant levels can be retained by CH4 and CH5. These results suggest that CH4 and CH5 could be considered as a potential multi-targeted ligand for the treatment of neurological disorders.

## Figures and Tables

**Table 1:** Inhibitions of MAO-A, MAO-B, AChE, BChE, and BACE-1 by halogenated trimethoxy chalcone derivatives ^a^.

Compounds	Residual Activity (%)					IC_50_ (µM)			SI ^b^
	MAO-A	MAO-B	AChE	BChE	BACE-1	MAO-A	MAO-B	BACE-1	
CH1	90.1 ± 7.51	70.7 ± 2.16	89.2 ± 2.23	71.1 ± 0.68	61.3 ± 0.078				
CH2	92.4 ± 1.10	84.3 ± 3.00	83.8 ± 7.96	74.5 ± 0.71	57.2 ± 0.46				
CH3	94.9 ± 7.19	75.4 ± 0.27	75.2 ± 3.43	79.9 ± 0.76	59.7 ± 0.077				
CH4	63.7 ± 1.02	0.93 ± 0.25	72.5 ± 7.22	73.8 ± 0.70	54.3 ± 2.20	12.7 ± 0.23	0.84 ± 0.025	13.6 ± 0.094	15.1
CH5	66.7 ± 3.96	0.38 ± 0.53	81.5 ± 5.31	67.1 ± 0.64	64.3 ± 0.74	14.4 ± 1.10	0.46 ± 0.12	19.8 ± 0.12	31.3
CH6	92.1 ± 0.75	19.6 ± 2.17	83.2 ± 1.23	84.6 ± 0.80	66.7 ± 0.45		4.17 ± 0.23		
Toloxatone						1.08 ± 0.025	-	-	
Lazabemide						-	0.11 ± 0.016	-	
Clorgyline						0.007 ± 0.0007	-	-	
Pargyline						-	0.14 ± 0.0059	-	
Quercetin						-	-	13.4 ± 0.035	

^a^ Results are the means ± standard errors of duplicate or triplicate experiments. ^b^ Selectivity index (SI) values are expressed for MAO-B as compared with MAO-A. IC_50_ values of donepezil for AChE and BChE were 0.0095 ± 0.0019 and 0.18 ± 0.0038 µM, respectively. Values for reference compounds were determined after preincubation with enzymes for 30 min.3.3. Kinetic Study.

**Table 2:** BBB assay of the CH compounds.

Compounds	Bibliography Pe (×10^−6^ cm/s) ^a^	Experimental Pe (×10^−6^ cm/s)	Prediction
Progesterone	9.3	9.02 ± 0.11	CNS+
Verapamil	16.0	15.53 ± 0.24	CNS+
Piroxicam	2.5	2.43 ± 0.30	CNS+/−
Lomefloxacin	1.1	1.12 ± 0.01	CNS−
Dopamine	0.2	0.22 ± 0.01	CNS−
CH1		13.22 ± 0.33	CNS+
CH2		14.06 ± 0.80	CNS+
CH3		15.33 ± 0.71	CNS+
CH4		14.56 ± 0.26	CNS+
CH5		15.65 ± 0.22	CNS+
CH6		15.22 ± 0.26	CNS+

CNS+ (high): Pe (10^−6^ cm/s) > 4.00; CNS− (low): Pe (10^−6^ cm/s) < 2.00; CNS± (uncertain): Pe (10^−6^ cm/s) from 4.00 to 2.00. ^a^ from [61].

## Data Availability

All data generated or analyzed during this study have been included in this article.

## Supplementary Materials

Available online at https://www.mdpi.com/article/10.3390/pharmaceutics13060850/s1, spectral interpretation; cytotoxicity studies; ROS Assay; MUSSEL Prediction; Toxicity Predicition.

## References

[1] Youdim M.B., Edmondson D., Tipton K.F. (2006). The therapeutic potential of monoamine oxidase inhibitors. Nat. Rev. Neurosci..

[2] Carradori S., Secci D., Petzer J.P. (2018). MAO inhibitors and their wider applications: A patent review. Expert Opin. Ther. Pat..

[3] Manzoor S., Hoda N. (2020). A comprehensive review of monoamine oxidase inhibitors as Anti-Alzheimer’s disease agents: A review. Eur. J. Med. Chem..

[4] Dezsi L., Vecsei L. (2017). Monoamine oxidase B inhibitors in Parkinson’s disease. CNS Neurol. Disord. Drug Targets.

[5] Riederer P., Müller T. (2017). Use of monoamine oxidase inhibitors in chronic neurodegeneration. Expert Opin. Drug Metab. Toxicol..

[6] Tripathi R.K.P., Ayyannan S.R. (2019). Monoamine oxidase-B inhibitors as potential neurotherapeutic agents: An overview and update. Med. Res. Rev..

[7] Guglielmi P., Carradori S., Ammazzalorso A., Secci D. (2019). Novel approaches to the discovery of selective human monoamine oxidase-B inhibitors: Is there room for improvement?. Expert Opin. Drug Discov..

[8] Hong R., Li X. (2018). Discovery of monoamine oxidase inhibitors by medicinal chemistry approaches. Medchemcomm.

[9] Youdim M.B., Bakhle Y.S. (2006). Monoamine oxidase: Isoforms and inhibitors in Parkinson’s disease and depressive illness. Br. J. Pharmacol..

[10] Ramsay R.R., Albreht A. (2018). Kinetics, mechanism, and inhibition of monoamine oxidase. J. Neural Transm..

[11] Ghosh A.K., Osswald H.L. (2014). BACE1 (β-secretase) inhibitors for the treatment of Alzheimer’s disease. Chem. Soc. Rev..

[12] Kumar D., Ganeshpurkar A., Kumar D., Modi G., Gupta S.K., Singh S.K. (2018). Secretase inhibitors for the treatment of Alzheimer’s disease: Long road ahead. Eur. J. Med. Chem..

[13] Moussa-Pacha N.M., Abdin S.M., Omar H.A., Alniss H., Al-Tel T.H. (2020). BACE1 inhibitors: Current status and future directions in treating Alzheimer’s disease. Med. Res. Rev..

[14] León R., Garcia A.G., Marco-Contelles J. (2013). Recent advances in the multitarget-directed ligands approach for the treatment of Alzheimer’s disease. Med. Res. Rev..

[15] Schaduangrat N., Prachayasittikul V., Choomwattana S., Wongchitrat P., Phopin K., Suwanjang W., Malik A.A., Vincent B., Nantasenamat C. (2019). Multidisciplinary approaches for targeting the secretase protein family as a therapeutic route for Alzheimer’s disease. Med. Res. Rev..

[16] Huang C., Zhang W., Sheng C., Zhang W., Xing C., Miao Z. (2017). Chalcone: A privileged structure in medicinal chemistry. Chem. Rev..

[17] Matos M.J., Vazquez-Rodriguez S., Uriarte E., Santana L. (2015). Potential pharmacological uses of chalcones: A patent review. Expert Opin. Ther. Pat..

[18] Singh P., Anand A., Kumar V. (2014). Recent developments in biological activities of chalcones: A mini review. Eur. J. Med. Chem..

[19] Sharma R., Kumar R., Kodwani R., Kapoor S., Khare A., Bansal R., Khurana S., Singh S., Thomas J., Roy B. (2015). A review on mechanisms of anti tumor activity of chalcones. Anticancer Agents Med. Chem..

[20] Nowakowska Z. (2007). A review of anti-infective and anti-inflammatory chalcones. Eur. J. Med. Chem..

[21] Rocha S., Ribeiro D., Fernandes E., Freitas M. (2020). A systematic review on anti-diabetic properties of chalcones. Curr. Med. Chem..

[22] Mathew B., Parambi D.G.T., Sivasankarapillai V.S., Uddin M.S., Suresh J., Mathew G.E., Joy M., Marathakam A., Gupta S.V. (2019). Perspective design of chalcones for the management of CNS disorders: A mini-review. CNS Neurol. Disord. Drug Targets.

[23] Wilcken R., Zimmermann M.O., Lange A., Joerger A.C., Boeckler F.M. (2013). Principles and applications of halogen bonding in medicinal chemistry and chemical biology. J. Med. Chem..

[24] Lu Y., Wang Y., Xu Z., Yan X., Luo X., Jiang H., Zhu W. (2009). C-X⋯H contacts in biomolecular systems: How they contribute to protein-ligand binding affinity. J. Phys. Chem. B..

[25] Mathew B., Carradori S., Guglielmi P., Uddin M.S., Kim H. (2021). New aspects of monoamine oxidase b inhibitors: The key role of halogens to open the golden door. Curr. Med. Chem..

[26] Mathew B., Mathew G.E., Uçar G., Baysal I., Suresh J., Vilapurathu J.K., Prakasan A., Suresh J.K., Thomas A. (2015). Development of fluorinated methoxylated chalcones as selective monoamine oxidase-B inhibitors: Synthesis, biochemistry and molecular docking studies. Bioorg. Chem..

[27] Morales-Camilo N., Salas C.O., Sanhueza C., Espinosa-Bustos C., Sepúlveda-Boza S., Reyes-Parada M., Gonzalez-Nilo F., Caroli-Rezende M., Fierro A. (2015). Synthesis, biological evaluation, and molecular simulation of chalcones and aurones as selective MAO-B inhibitors. Chem. Biol. Drug Des..

[28] Choi J.W., Jang B.K., Cho N.C., Park J.H., Yeon S.K., Ju E.J., Lee Y.S., Han G., Pae A.N., Kim D.J. (2015). Synthesis of a series of unsaturated ketone derivatives as selective and reversible monoamine oxidase inhibitors. Bioorg. Med. Chem..

[29] Hammuda A., Shalaby R., Rovida S., Edmondson D.E., Binda C., Khalil A. (2016). Design and synthesis of novel chalcones as potent selective monoamine oxidase-B inhibitors. Eur. J. Med. Chem..

[30] Mathew B., Uçar G., Mathew G.E., Mathew S., Purapurath K.P., Moolayil F., Mohan S., Gupta S.V. (2016). Monoamine oxidase inhibitory activity: Methyl- versus Chloro chalcone derivatives. Chem. Med. Chem..

[31] Mathew B., Mathew G.E., Ucar G., Joy M., Nafna E.K., Lohidakshan K.K., Suresh J. (2017). Monoamine oxidase inhibitory activity of methoxy-substituted chalcones. Int. J. Biol. Macromol..

[32] Chimenti F., Fioravanti R., Bolasco A., Chimenti P., Secci D., Rossi F., Yáñez M., Orallo F., Ortuso F., Alcaro S. (2009). Chalcones: A valid scaffold for monoamine oxidases inhibitors. J. Med. Chem..

[33] Minders C., Petzer J.P., Petzer A., Lourens A.C. (2015). Monoamine oxidase inhibitory activities of heterocyclic chalcones. Bioorg. Med. Chem. Lett..

[34] Sasidharan R., Manju S.L., Uçar G., Baysal I., Mathew B. (2016). Identification of indole-based chalcones: Discovery of a potent, selective, and reversible class of MAO-B inhibitors. Arch. Pharm..

[35] Mathew B., Haridas A., Uçar G., Baysal I., Joy M., Mathew G.E., Lakshmanan B., Jayaprakash V. (2016). Synthesis, biochemistry, and computational studies of brominated thienyl chalcones: A new class of reversible MAO-B Inhibitors. Chem. Med. Chem..

[36] Xiao G., Li Y., Qiang X., Xu R., Zheng Y., Cao Z., Luo L., Yang X., Sang Z., Su F. (2017). Design, synthesis and biological evaluation of 4′-aminochalcone-rivastigmine hybrids as multifunctional agents for the treatment of Alzheimer’s disease. Bioorg. Med. Chem..

[37] Cao Z., Yang J., Xu R., Song Q., Zhang X., Liu H., Qiang X., Li Y., Tan Z., Deng Y. (2018). Design, synthesis and evaluation of 4′-OH-flurbiprofen-chalcone hybrids as potential multifunctional agents for Alzheimer’s disease treatment. Bioorg. Med. Chem..

[38] Sasidharan R., Baek S.C., Sreedharannair Leelabaiamma M., Kim H., Mathew B. (2018). Imidazole bearing chalcones as a new class of monoamine oxidase inhibitors. Biomed. Pharmacother..

[39] Suresh J., Baek S.C., Ramakrishnan S.P., Kim H., Mathew B. (2018). Discovery of potent and reversible MAO-B inhibitors as furanochalcones. Int. J. Biol. Macromol..

[40] Reeta, Baek S.C., Lee J.P., Rangarajan T.M., Ayushee, Singh R.P., Singh M., Mangiatordi G.F., Nicolotti O., Kim H. (2019). Ethyl acetohydroxamate incorporated chalcones: Unveiling a novel class of chalcones for multitarget monoamine oxidase-B inhibitors against Alzheimer’s disease. CNS Neurol. Disord. Drug Targets.

[41] Mathew B., Baek S.C., Thomas Parambi D.G., Lee J.P., Mathew G.E., Jayanthi S., Vinod D., Rapheal C., Devikrishna V., Kondarath S.S. (2019). Potent and highly selective dual-targeting monoamine oxidase-B inhibitors: Fluorinated chalcones of morpholine versus imidazole. Arch. Pharm..

[42] Parambi D.G.T., Oh J.M., Baek S.C., Lee J.P., Tondo A.R., Nicolotti O., Kim H., Mathew B. (2019). Design, synthesis and biological evaluation of oxygenated chalcones as potent and selective MAO-B inhibitors. Bioorg. Chem..

[43] Sang Z., Wang K., Zhang P., Shi J., Liu W., Tan Z. (2019). Design, synthesis, in-silico and biological evaluation of novel chalcone derivatives as multi-function agents for the treatment of Alzheimer’s disease. Eur. J. Med. Chem..

[44] Zhang X., Song Q., Cao Z., Li Y., Tian C., Yang Z., Zhang H., Deng Y. (2019). Design, synthesis and evaluation of chalcone Mannich base derivatives as multifunctional agents for the potential treatment of Alzheimer’s disease. Bioorg. Chem..

[45] Shalaby R., Petzer J.P., Petzer A., Ashraf U.M., Atari E., Alasmari F., Kumarasamy S., Sari Y., Khalil A. (2019). SAR and molecular mechanism studies of monoamine oxidase inhibition by selected chalcone analogs. J. Enzyme Inhib. Med. Chem..

[46] Bai P., Wang K., Zhang P., Shi J., Cheng X., Zhang Q., Zheng C., Cheng Y., Yang J., Lu X. (2019). Development of chalcone-O-alkylamine derivatives as multifunctional agents against Alzheimer’s disease. Eur. J. Med. Chem..

[47] Oh J.M., Rangarajan T.M., Chaudhary R., Singh R.P., Singh M., Singh R.P., Tondo A.R., Gambacorta N., Nicolotti O., Mathew B. (2020). Novel class of chalcone oxime ethers as potent monoamine oxidase-B and acetylcholinesterase inhibitors. Molecules.

[48] Jeong G.S., Kaipakasseri S., Lee S.R., Marraiki N., Batiha G.E., Dev S., Palakkathondi A., Kavully F.S., Gambacorta N., Nicolotti O. (2020). Selected 1,3-benzodioxine-containing chalcones as multipotent oxidase and acetylcholinesterase inhibitors. ChemMedChem.

[49] Kong Z., Sun D., Jiang Y., Hu Y. (2020). Design, synthesis, and evaluation of 1, 4-benzodioxan-substituted chalcones as selective and reversible inhibitors of human monoamine oxidase B. J. Enzyme Inhib. Med. Chem..

[50] Sasidharan R., Eom B.H., Heo J.H., Park J.E., Abdelgawad M.A., Musa A., Gambacorta N., Nicolotti O., Manju S.L., Mathew B. (2021). Morpholine-based chalcones as dual-acting monoamine oxidase-B and acetylcholinesterase inhibitors: Synthesis and biochemical investigations. J. Enzyme Inhib. Med. Chem..

[51] Zhang X., Rakesh K.P., Bukhari S.N.A., Balakrishna M., Manukumar H.M., Qin H.L. (2018). Multi-targetable chalcone analogs to treat deadly Alzheimer’s disease: Current view and upcoming advice. Bioorg. Chem..

[52] Guglielmi P., Mathew B., Secci D., Carradori S. (2020). Chalcones: Unearthing their therapeutic possibility as monoamine oxidase B inhibitors. Eur. J. Med. Chem..

[53] Mathew B. (2020). Privileged pharmacophore of FDA approved drugs in combination with chalcone framework: A new hope for alzheimer’s treatment. Comb. Chem. High Throughput Screen..

[54] Burmaoglu S., Kazancioglu E.A., Kaya R., Kazancioglu M., Karaman M., Algul O., Gulcin I. (2020). Synthesis of novel organohalogen chalcone derivatives and screening of their molecular docking study and some enzymes inhibition effects. J. Mol. Struct..

[55] Mathew B., Baek S.C., Grace Thomas Parambi D., Pil Lee J., Joy M., Annie Rilda P.R., Randev R.V., Nithyamol P., Vijayan V., Inasu S.T. (2018). Selected aryl thiosemicarbazones as a new class of multi-targeted monoamine oxidase inhibitors. MedChemComm.

[56] Mathew B., Oh J.M., Baty R.S., Batiha G.E., Parambi D.G.T., Gambacorta N., Nicolotti O., Kim H. (2021). Piperazine-substituted chalcones: A new class of MAO-B, AChE, and BACE-1 inhibitors for the treatment of neurological disorders. Environ. Sci. Pollut. Res..

[57] Nair A.S., Oh J.-M., Koyiparambath V.P., Kumar S., Sudevan S.T., Soremekun O., Soliman M.E., Khames A., Abdelgawad M.A., Pappachen L.K. (2021). Development of halogenated pyrazolines as selective monoamine oxidase-B Inhibitors: Deciphering via molecular dynamics approach. Molecules.

[58] Oh J.M., Jang H.J., Kim W.J., Kang M.G., Baek S.C., Lee J.P., Park D., Oh S.R., Kim H. (2020). Calycosin and 9-O-methylretusin isolated from *Maackia amurensis* as potent and selective reversible inhibitors of human monoamine oxidase-B. Int. J. Biol. Macromol..

[59] Fotakis G., Timbrell J.A. (2006). In vitro cytotoxicity assays: Comparison of LDH, neutral red, MTT and protein assay in hepatoma cell lines following exposure to cadmium chloride. Toxicol. Lett..

[60] Jambunathan N. (2010). Determination and detection of reactive oxygen species (ROS), lipid peroxidation, and electrolyte leakage in plants. Methods Mol. Biol..

[61] Di L., Kerns E.H., Fan K., McConnell O.J., Carter G.T. (2003). High throughput artificial membrane permeability assay for blood-brain barrier. Eur. J. Med. Chem..

[62] Son S.Y., Ma J., Kondou Y., Yoshimura M., Yamashita E., Tsukihara T. (2008). Structure of human monoamine oxidase A at 2.2-A resolution: The control of opening the entry for substrates/inhibitors. Proc. Natl. Acad. Sci. USA.

[63] Binda C., Wang J., Pisani L., Caccia C., Carotti A., Salvati P., Edmondson D.E., Mattevi A. (2007). Structures of human monoamine oxidase B complexes with selective noncovalent inhibitors: Safinamide and coumarin analogs. J. Med. Chem..

[64] Xu Y., Li M., Greenblatt H., Chen W., Paz A., Dym O., Peleg Y., Chen T., Shen X., He J. (2012). Flexibility of the flap in the active site of BACE1 as revealed by crystal structures and molecular dynamics simulations. Acta Cryst. D.

[65] (2016). Schrödinger Release 2020-4: Protein Preparation Wizard.

[66] Madhavi Sastry G., Adzhigirey M., Day T., Annabhimoju R., Sherman W. (2013). Protein and Ligand Preparation: Parameters, protocols, and influence on virtual screening enrichments. J. Comput. Aided. Mol. Des..

[67] (2020). Schrödinger Release 2020-4: LigPrep.

[68] Friesner R.A., Banks J.L., Murphy R.B., Halgren T.A., Klicic J.J., Mainz D.T., Repasky M.P., Knoll E.H., Shelley M., Perry J.K. (2004). Glide: A new approach for rapid, accurate docking and scoring. 1. Method and assessment of docking accuracy. J. Med. Chem..

[69] http://www.caesar-project.eu.

[70] http://predherg.labmol.com.br/.

[71] Mathew B., Adeniyi A.A., Joy M., Mathew G.E., Ashona Singh-Pillay A.S., Sudarsanakumar C., Soliman M.E.S., Suresh J. (2017). Anti-oxidant behavior of functionalized chalcone-a combined quantum chemical and crystallographic structural investigation. J. Mol. Struct..

[72] Kumar V., Sharma N., Maitra S.S. (2007). In vitro and in vivo toxicity assessment of nanoparticles. Int. Nano Lett..

[73] Zuo L., Zhou T., Pannell B.K., Ziegler A.C., Best T.M. (2015). Biological and physiological role of reactive oxygen species—The good, the bad and the ugly. Acta Physiol. (Oxf.).

[74] Park C., Cha H.J., Hong S.H., Kim G.Y., Kim S., Kim H.S., Kim B.W., Jeon Y.J., Choi Y.H. (2019). Protective effect of phloroglucinol on oxidative stress-induced DNA damage and apoptosis through activation of the Nrf2/HO-1 signaling pathway in HaCaT human keratinocytes. Mar. Drugs.

[75] Alberga D., Trisciuzzi D., Montaruli M., Leonetti F., Mangiatordi G.F., Nicolotti O. (2019). A new approach for drug target and bioactivity prediction: The multifingerprint similarity search algorithm (MuSSeL). J. Chem. Inf. Model..

[76] Montaruli M., Alberga D., Ciriaco F., Trisciuzzi D., Tondo A.R., Mangiatordi G.F., Nicolotti O. (2019). Accelerating drug discovery by early protein drug target prediction based on a multi-fingerprint similarity search. Molecules.

[77] Nicolotti O., Benfenati E., Carotti A., Gadaleta D., Gissi A., Mangiatordi G.F., Novellino E. (2014). REACH and in silico methods: An attractive opportunity for medicinal chemists. Drug Discov. Today.

[78] Cavalluzzi M.M., Imbrici P., Gualdani R., Stefanachi A., Mangiatordi G.F., Lentini G., Nicolotti O. (2020). Human ether-à-go-go-related potassium channel: Exploring SAR to improve drug design. Drug Discov. Today.

[79] Mangiatordi G.F., Alberga D., Altomare C.D., Carotti A., Catto M., Cellamare S., Gadaleta D., Lattanzi G., Leonetti F., Pisani L. (2016). Mind the gap! A journey towards computational toxicology. Mol. Inform..

[80] Ferrari T., Gini G. (2010). An open source multistep model to predict mutagenicity from statistical analysis and relevant structural alerts. Chem Cent. J..

[81] Braga R.C., Alves V.M., Silva M.F., Muratov E., Fourches D., Lião L.M., Tropsha A., Andrade C.H. (2015). Pred-hERG: A novel web-accessible computational tool for predicting cardiac toxicity. Mol. Inform..

