# Supplementary material for: Trimethoxylated Halogenated Chalcones as Dual Inhibitors of MAO-B and BACE-1 for the Treatment of Neurodegenerative Disorders
Source: Pharmaceutics. 2021 Jun 8;13(6):850. doi: 10.3390/pharmaceutics13060850 (PMC8226672; doi:10.3390/pharmaceutics13060850)
Supplement: Supplementary file 1 [file pharmaceutics-13-00850-s001.zip › pharmaceutics-1241084-supplementary.pdf]

pharmaceutics

Supplementary Materials: Trimethoxylated Halogenated Chalcones as Dual Inhibitors of MAO-B and BACE-1 for the Treatment of Neurodegenerative Disorders

Vishal Payyalot Koyiparambath, Jong Min Oh, Ahmed Khames, Mohamed A. Abdelgawad, Aathira Sujathan Nair, Lekshmi R. Nath, Nicola Gambacorta, Fulvio Ciriaco, Orazio Nicolotti, Hoon Kim and Bijo Mathew

Contents

1.1. Spectral Interpretation

1.2. Cytotoxicity Studies

1.3. ROS Assay

1.4. Mussel Prediction

1.5. Toxicity Prediction

1.1. Spectral Interpretation

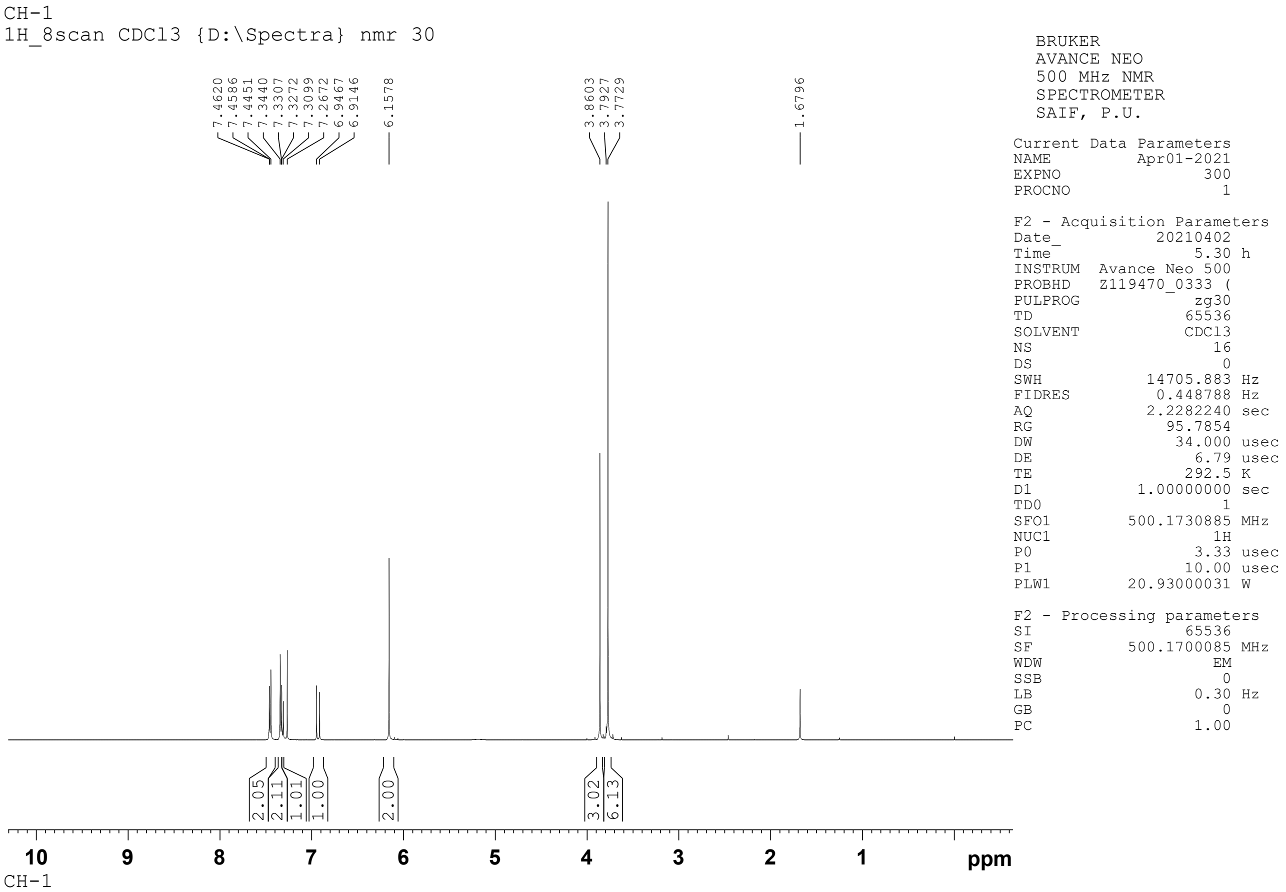

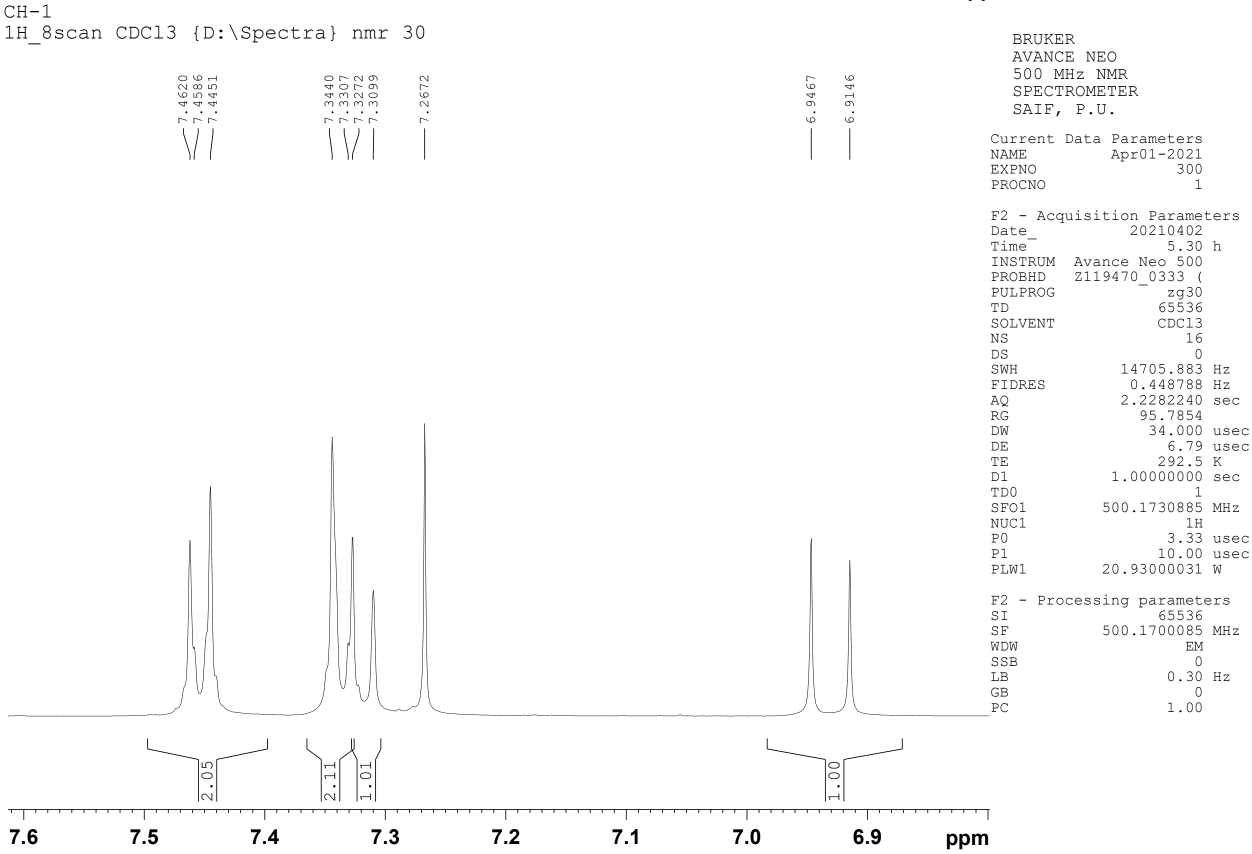

CH-1
1H_8scan CDCl3 {D:\Spectra} nmr 30

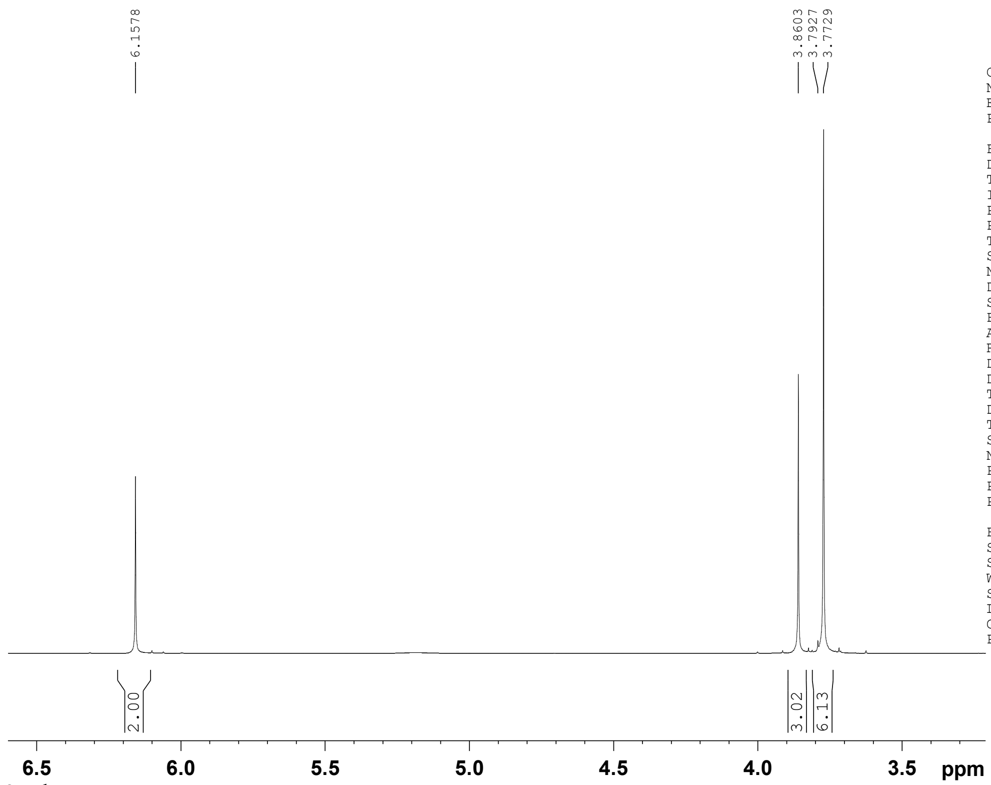

BRUKER
AVANCE NEO
500 MHz NMR
SPECTROMETER
SAIF, P.U.

Current Data Parameters	
NAME	Apr01-2021
EXPNO	300
PROCNO	1

F2 - Acquisition Parameters	
Date_	20210402
Time	5.30 h
INSTRUM	Avance Neo 500
PROBHD	Z119470_0333 (
PULPROG	zg30
TD	65536
SOLVENT	CDCl3
NS	16
DS	0
SWH	14705.883 Hz
FIDRES	0.448788 Hz
AQ	2.2282240 sec
RG	95.7854
DW	34.000 usec
DE	6.79 usec
TE	292.5 K
D1	1.00000000 sec
TD0	1
SFO1	500.1730885 MHz
NUC1	1H
P0	3.33 usec
P1	10.00 usec
PLW1	20.93000031 W

F2 - Processing parameters	
SI	65536
SF	500.1700085 MHz
WDW	EM
SSB	0
LB	0.30 Hz
GB	0
PC	1.00

CH-1
C13CPD CDCl3 {D:\Spectra} nmr 30

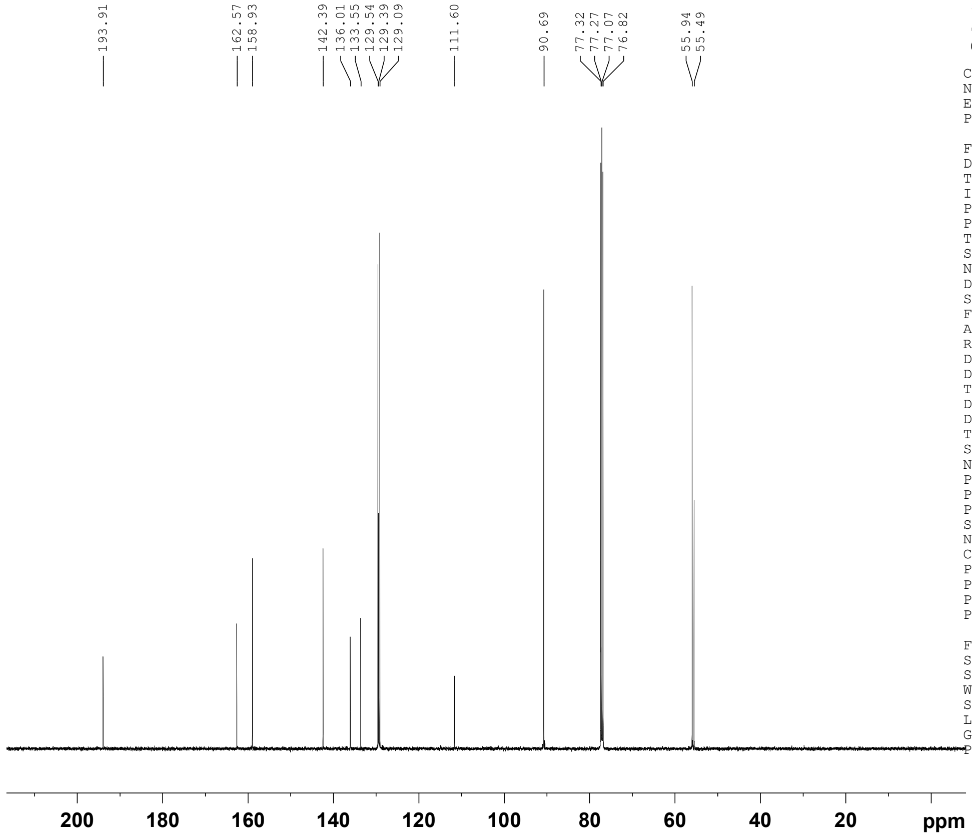

BRUKER
AVANCE NEO
500 MHz NMR SPECTROMETER
SAIF, PANJAB UNIVERSITY,
CHANDIGARH

Current Data Parameters	
NAME	Apr01-2021
EXPNO	301
PROCNO	1

F2 - Acquisition Parameters	
Date_	20210402
Time	6.22 h
INSTRUM	Avance Neo 500
PROBHD	Z119470_0333 (
PULPROG	zgpg30
TD	65536
SOLVENT	CDCl3
NS	1024
DS	4
SWH	37037.035 Hz
FIDRES	1.130281 Hz
AQ	0.8847360 sec
RG	101
DW	13.500 usec
DE	6.50 usec
TE	293.4 K
D1	2.00000000 sec
D11	0.03000000 sec
TD0	1
SFO1	125.7804233 MHz
NUC1	13C
P0	3.33 usec
P1	10.00 usec
PLW1	83.14099884 W
SFO2	500.1720007 MHz
NUC2	1H
CPDPRG[2	waltz65
PCPD2	80.00 usec
PLW2	20.93000031 W
PLW12	0.32703000 W
PLW13	0.16449000 W

F2 - Processing parameters	
SI	32768
SF	125.7678465 MHz
WDW	EM
SSB	0
LB	1.00 Hz
GB	0
PC	1.40

CH-1
C13CPD CDCl3 {D:\Spectra} nmr 30

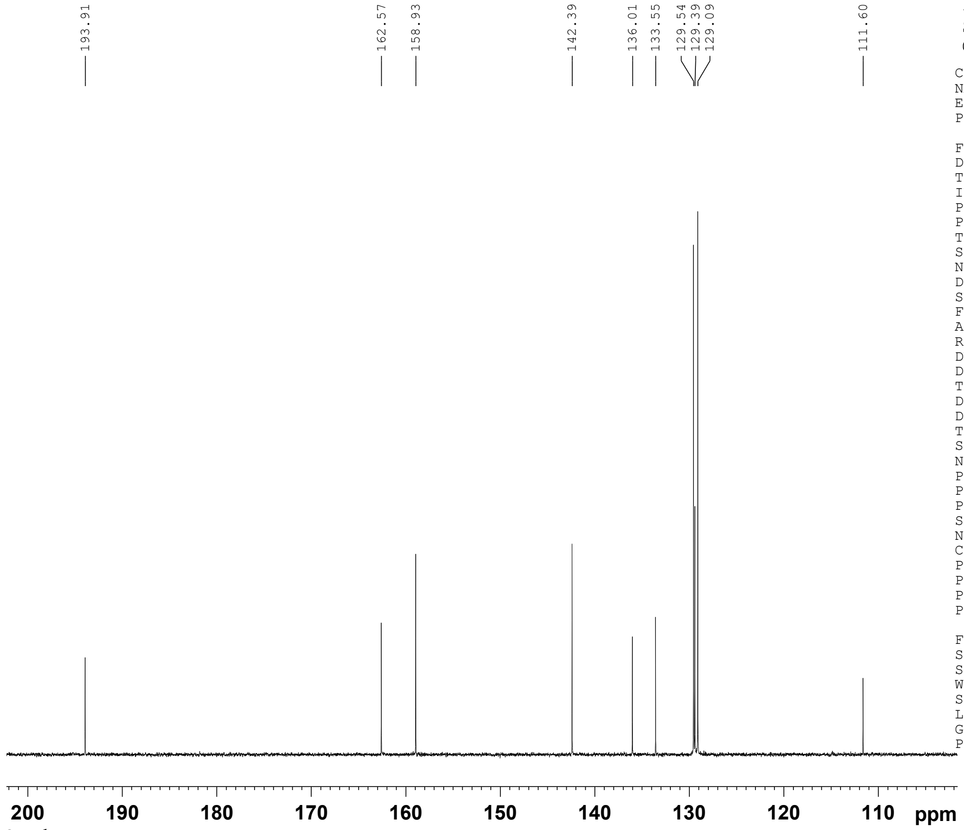

BRUKER
AVANCE NEO
500 MHz NMR SPECTROMETER
SAIF, PANJAB UNIVERSITY,
CHANDIGARH

Current Data Parameters
NAME Apr01-2021
EXPNO 301
PROCNO 1

F2 - Acquisition Parameters
Date_ 20210402
Time 6.22 h
INSTRUM Avance Neo 500
PROBHD Z119470_0333 (
PULPROG zgpg30
TD 65536
SOLVENT CDCl3
NS 1024
DS 4
SWH 37037.035 Hz
FIDRES 1.130281 Hz
AQ 0.8847360 sec
RG 101
DW 13.500 usec
DE 6.50 usec
TE 293.4 K
D1 2.00000000 sec
D11 0.03000000 sec
TD0 1
SFO1 125.7804233 MHz
NUC1 13C
P0 3.33 usec
P1 10.00 usec
PLW1 83.14099884 W
SFO2 500.1720007 MHz
NUC2 1H
CPDPRG[2 waltz65
PCPD2 80.00 usec
PLW2 20.93000031 W
PLW12 0.32703000 W
PLW13 0.16449000 W

F2 - Processing parameters
SI 32768
SF 125.7678465 MHz
WDW EM
SSB 0
LB 1.00 Hz
GB 0
PC 1.40

CH-1
C13CPD CDCl3 {D:\Spectra} nmr 30

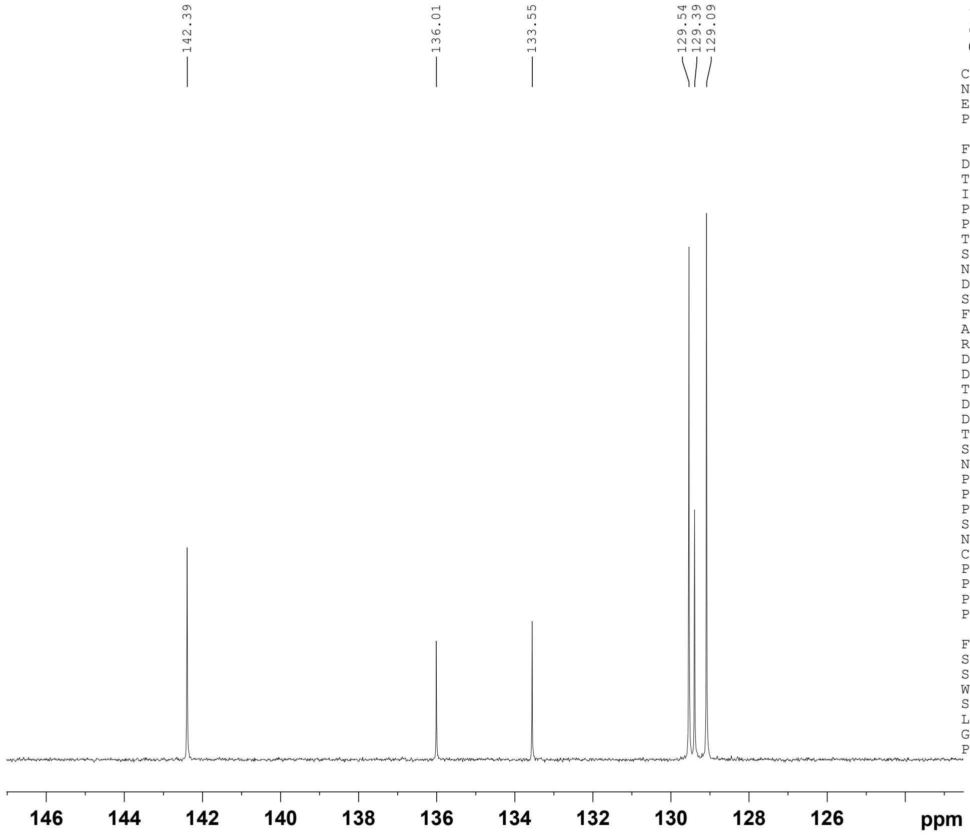

BRUKER
AVANCE NEO
500 MHz NMR SPECTROMETER
SAIF, PANJAB UNIVERSITY,
CHANDIGARH

Current Data Parameters
NAME Apr01-2021
EXPNO 301
PROCNO 1

F2 - Acquisition Parameters
Date_ 20210402
Time 6.22 h
INSTRUM Avance Neo 500
PROBHD Z119470_0333 (
PULPROG zgpg30
TD 65536
SOLVENT CDCl3
NS 1024
DS 4
SWH 37037.035 Hz
FIDRES 1.130281 Hz
AQ 0.8847360 sec
RG 101
DW 13.500 usec
DE 6.50 usec
TE 293.4 K
D1 2.00000000 sec
D11 0.03000000 sec
TD0 1
SFO1 125.7804233 MHz
NUC1 13C
P0 3.33 usec
P1 10.00 usec
PLW1 83.14099884 W
SFO2 500.1720007 MHz
NUC2 1H
CPDPRG[2 waltz65
PCPD2 80.00 usec
PLW2 20.93000031 W
PLW12 0.32703000 W
PLW13 0.16449000 W

F2 - Processing parameters
SI 32768
SF 125.7678465 MHz
WDW EM
SSB 0
LB 1.00 Hz
GB 0
PC 1.40

CH-1
C13CPD CDCl3 {D:\Spectra} nmr 30

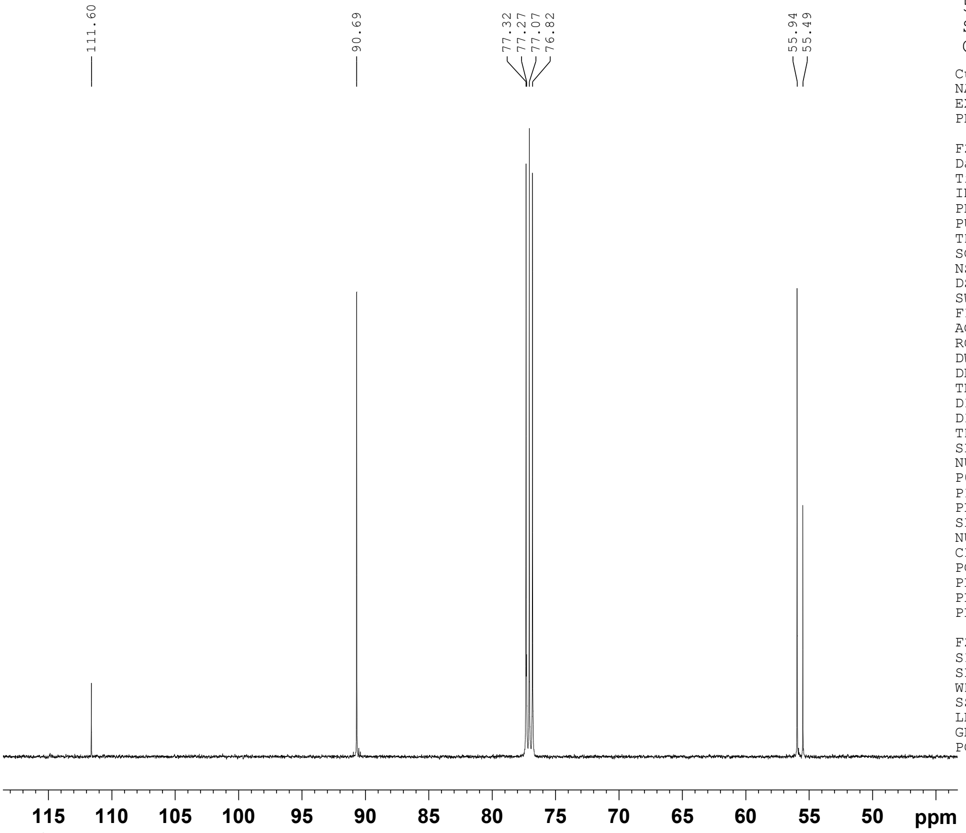

BRUKER
AVANCE NEO
500 MHz NMR SPECTROMETER
SAIF, PANJAB UNIVERSITY,
CHANDIGARH

Current Data Parameters
NAME Apr01-2021
EXPNO 301
PROCNO 1

F2 - Acquisition Parameters
Date_ 20210402
Time 6.22 h
INSTRUM Avance Neo 500
PROBHD Z119470_0333 (
PULPROG zgpg30
TD 65536
SOLVENT CDCl3
NS 1024
DS 4
SWH 37037.035 Hz
FIDRES 1.130281 Hz
AQ 0.8847360 sec
RG 101
DW 13.500 usec
DE 6.50 usec
TE 293.4 K
D1 2.00000000 sec
D11 0.03000000 sec
TD0 1
SFO1 125.7804233 MHz
NUC1 13C
P0 3.33 usec
P1 10.00 usec
PLW1 83.14099884 W
SFO2 500.1720007 MHz
NUC2 1H
CPDPRG[2 waltz65
PCPD2 80.00 usec
PLW2 20.93000031 W
PLW12 0.32703000 W
PLW13 0.16449000 W

F2 - Processing parameters
SI 32768
SF 125.7678465 MHz
WDW EM
SSB 0
LB 1.00 Hz
GB 0
PC 1.40

CH-2
1H_8scan CDCl3 {D:\Spectra} nmr 31

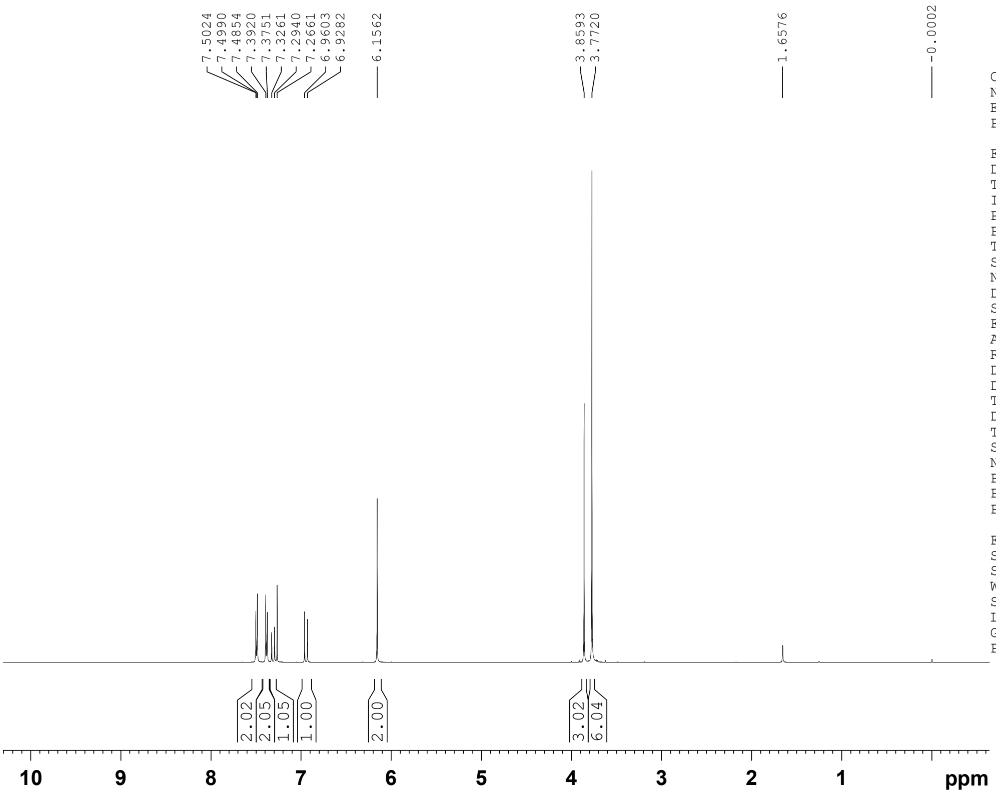

BRUKER
AVANCE NEO
500 MHz NMR
SPECTROMETER
SAIF, P.U.

Current Data Parameters
NAME Apr01-2021
EXPNO 310
PROCNO 1

F2 - Acquisition Parameters
Date_ 20210402
Time 6.24 h
INSTRUM Avance Neo 500
PROBHD Z119470_0333 (
PULPROG zg30
TD 65536
SOLVENT CDCl3
NS 16
DS 0
SWH 14705.883 Hz
FIDRES 0.448788 Hz
AQ 2.2282240 sec
RG 95.7854
DW 34.000 usec
DE 6.79 usec
TE 293.0 K
D1 1.00000000 sec
TD0 1
SFO1 500.1730885 MHz
NUC1 1H
P0 3.33 usec
P1 10.00 usec
PLW1 20.93000031 W

F2 - Processing parameters
SI 65536
SF 500.1700089 MHz
WDW EM
SSB 0
LB 0.30 Hz
GB 0
PC 1.00

CH-2
1H_8scan CDCl3 {D:\Spectra} nmr 31

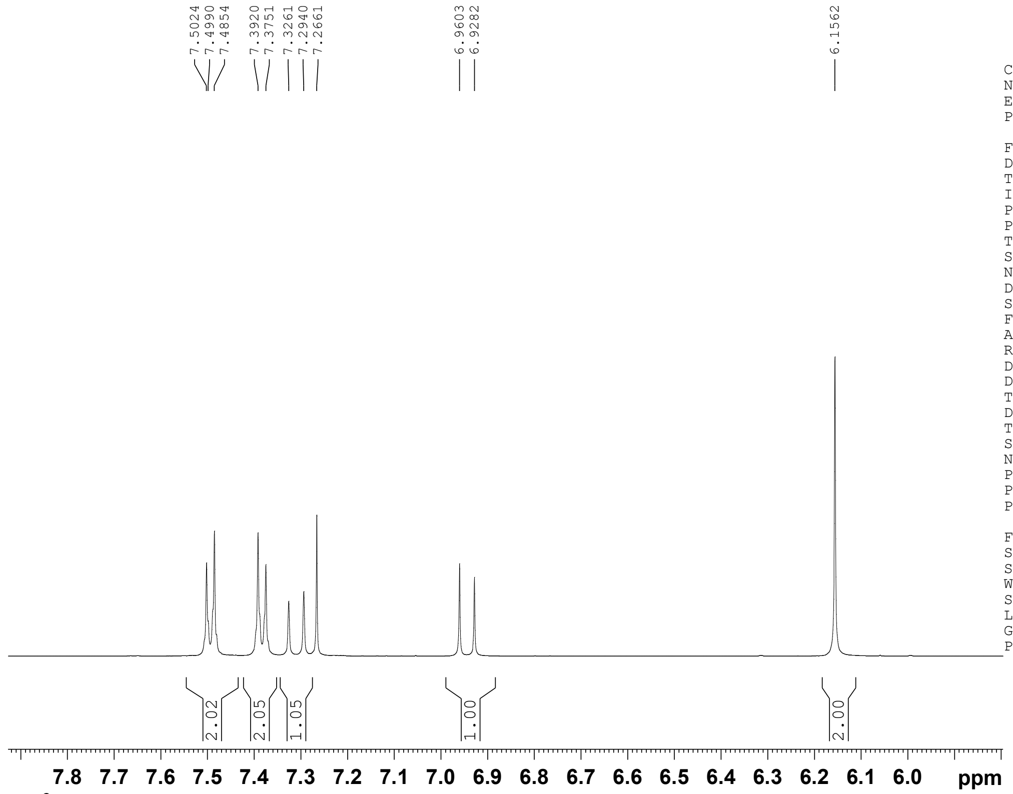

BRUKER
AVANCE NEO
500 MHz NMR
SPECTROMETER
SAIF, P.U.

Current Data Parameters
NAME Apr01-2021
EXPNO 310
PROCNO 1

F2 - Acquisition Parameters
Date_ 20210402
Time 6.24 h
INSTRUM Avance Neo 500
PROBHD Z119470_0333 (
PULPROG zg30
TD 65536
SOLVENT CDCl3
NS 16
DS 0
SWH 14705.883 Hz
FIDRES 0.448788 Hz
AQ 2.2282240 sec
RG 95.7854
DW 34.000 usec
DE 6.79 usec
TE 293.0 K
D1 1.00000000 sec
TD0 1
SFO1 500.1730885 MHz
NUC1 1H
P0 3.33 usec
P1 10.00 usec
PLW1 20.93000031 W

F2 - Processing parameters
SI 65536
SF 500.1700089 MHz
WDW EM
SSB 0
LB 0.30 Hz
GB 0
PC 1.00

CH-2
1H_8scan CDCl3 {D:\Spectra} nmr 31

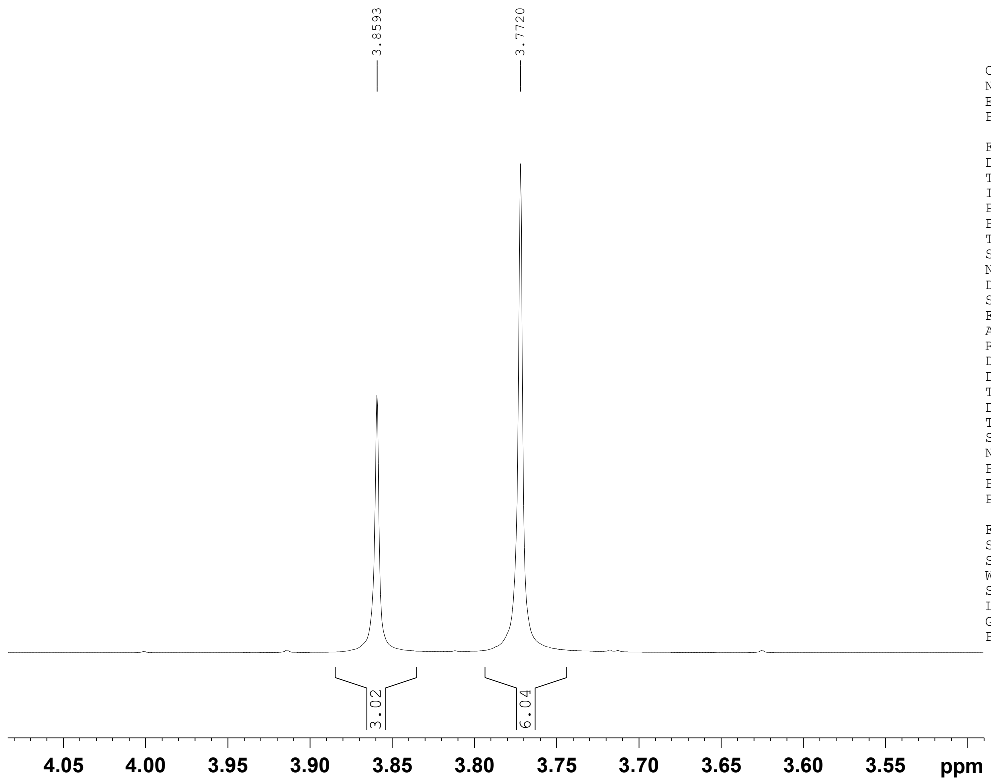

BRUKER
AVANCE NEO
500 MHz NMR
SPECTROMETER
SAIF, P.U.

Current Data Parameters
NAME Apr01-2021
EXPNO 310
PROCNO 1

F2 - Acquisition Parameters
Date_ 20210402
Time 6.24 h
INSTRUM Avance Neo 500
PROBHD Z119470_0333 (
PULPROG zg30
TD 65536
SOLVENT CDCl3
NS 16
DS 0
SWH 14705.883 Hz
FIDRES 0.448788 Hz
AQ 2.2282240 sec
RG 95.7854
DW 34.000 usec
DE 6.79 usec
TE 293.0 K
D1 1.00000000 sec
TD0 1
SFO1 500.1730885 MHz
NUC1 1H
P0 3.33 usec
P1 10.00 usec
PLW1 20.93000031 W

F2 - Processing parameters
SI 65536
SF 500.1700089 MHz
WDW EM
SSB 0
LB 0.30 Hz
GB 0
PC 1.00

CH-2
C13CPD CDCl3 {D:\Spectra} nmr 31

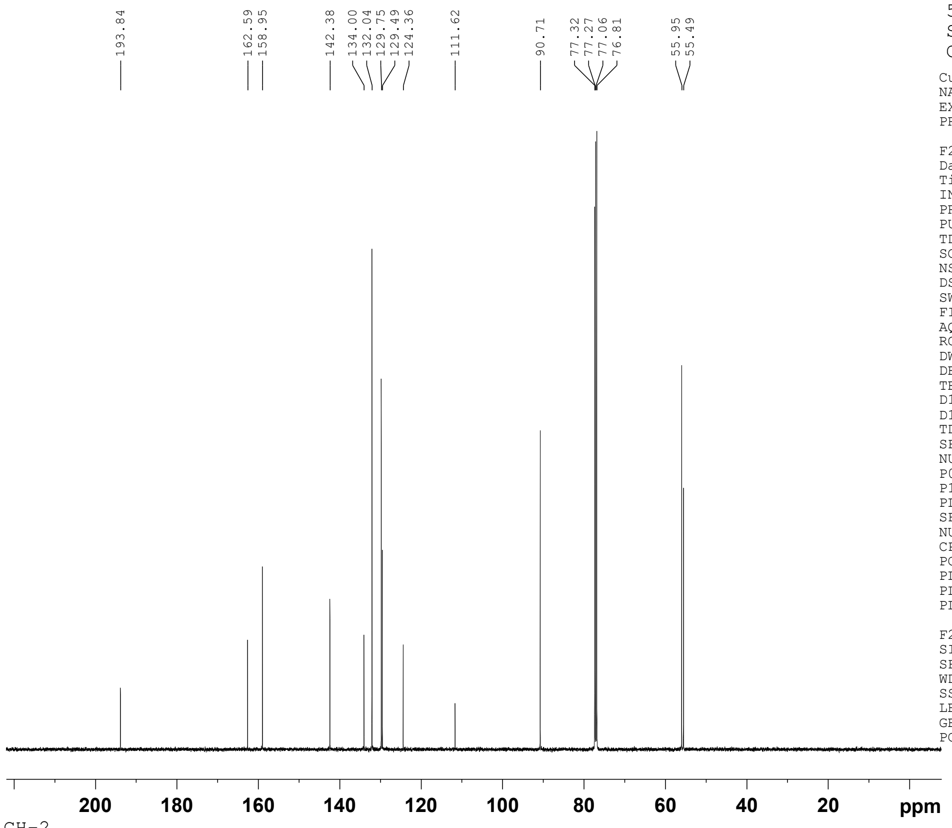

BRUKER
AVANCE NEO
500 MHz NMR SPECTROMETER
SAIF, PANJAB UNIVERSITY,
CHANDIGARH

Current Data Parameters
NAME Apr01-2021
EXPNO 311
PROCNO 1

F2 - Acquisition Parameters
Date_ 20210402
Time 7.15 h
INSTRUM Avance Neo 500
PROBHD Z119470_0333 (
PULPROG zgpg30
TD 65536
SOLVENT CDCl3
NS 1024
DS 4
SWH 37037.035 Hz
FIDRES 1.130281 Hz
AQ 0.8847360 sec
RG 101
DW 13.500 usec
DE 6.50 usec
TE 295.2 K
D1 2.00000000 sec
D11 0.03000000 sec
TD0 1
SFO1 125.7804233 MHz
NUC1 13C
P0 3.33 usec
P1 10.00 usec
PLW1 83.14099884 W
SFO2 500.1720007 MHz
NUC2 1H
CPDPRG[2 waltz65
PCPD2 80.00 usec
PLW2 20.93000031 W
PLW12 0.32703000 W
PLW13 0.16449000 W

F2 - Processing parameters
SI 32768
SF 125.7678465 MHz
WDW EM
SSB 0
LB 1.00 Hz
GB 0
PC 1.40

CH-2
C13CPD CDCl3 {D:\Spectra} nmr 31

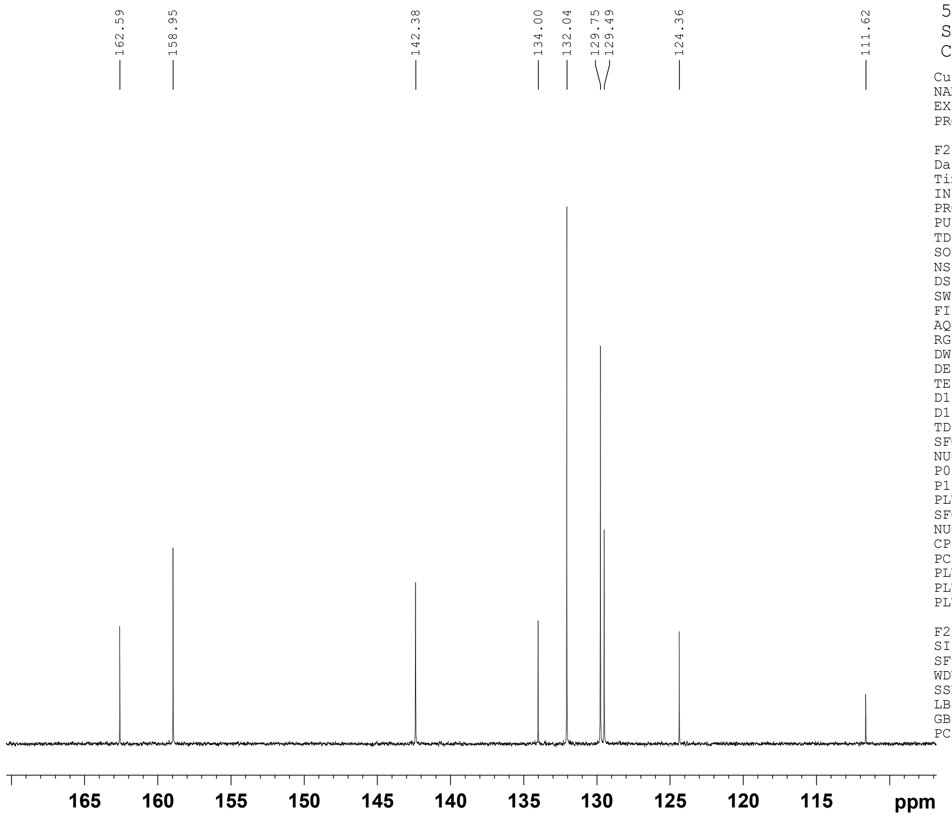

BRUKER
AVANCE NEO
500 MHz NMR SPECTROMETER
SAIF, PANJAB UNIVERSITY,
CHANDIGARH

Current Data Parameters
NAME Apr01-2021
EXPNO 311
PROCNO 1

F2 - Acquisition Parameters
Date_ 20210402
Time 7.15 h
INSTRUM Avance Neo 500
PROBHD Z119470_0333 (
PULPROG zgpg30
TD 65536
SOLVENT CDCl3
NS 1024
DS 4
SWH 37037.035 Hz
FIDRES 1.130281 Hz
AQ 0.8847360 sec
RG 101
DW 13.500 usec
DE 6.50 usec
TE 295.2 K
D1 2.00000000 sec
D11 0.03000000 sec
TD0 1
SFO1 125.7804233 MHz
NUC1 13C
P0 3.33 usec
P1 10.00 usec
PLW1 83.14099884 W
SFO2 500.1720007 MHz
NUC2 1H
CPDPRG[2 waltz65
PCPD2 80.00 usec
PLW2 20.93000031 W
PLW12 0.32703000 W
PLW13 0.16449000 W

F2 - Processing parameters
SI 32768
SF 125.7678465 MHz
WDW EM
SSB 0
LB 1.00 Hz
GB 0
PC 1.40

CH-2
C13CPD CDCl3 {D:\Spectra} nmr 31

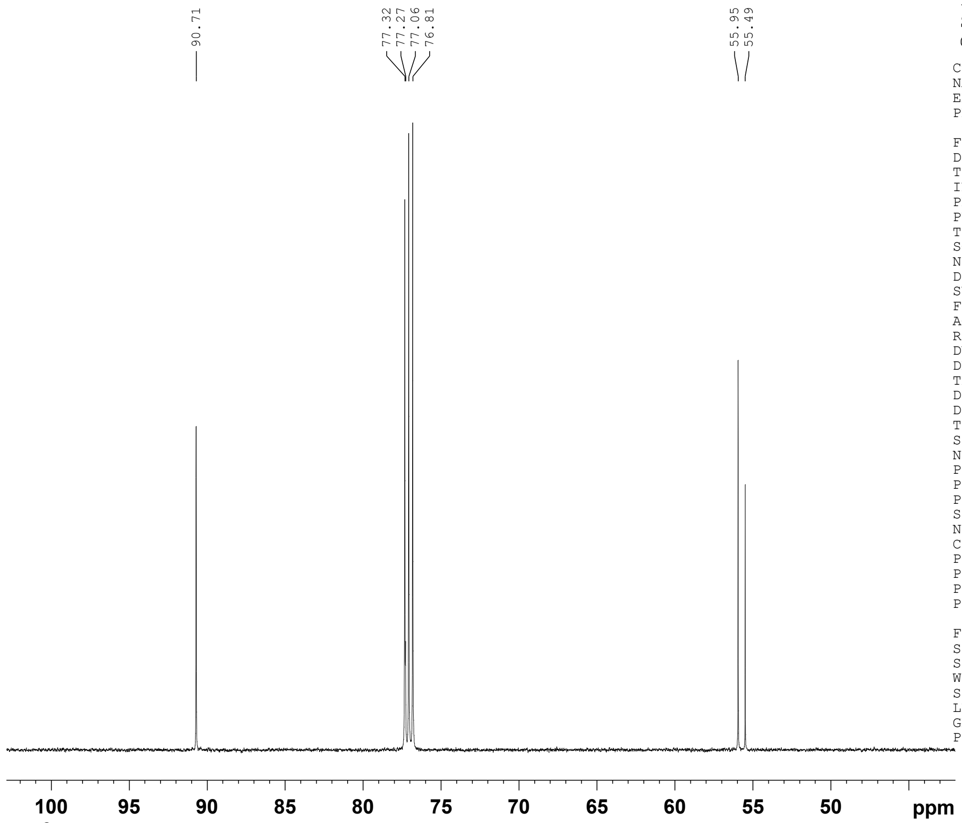

BRUKER
AVANCE NEO
500 MHz NMR SPECTROMETER
SAIF, PANJAB UNIVERSITY,
CHANDIGARH

Current Data Parameters

NAME	Apr01-2021
EXPNO	311
PROCNO	1

F2 - Acquisition Parameters

Date_	20210402
Time	7.15 h
INSTRUM	Avance Neo 500
PROBHD	Z119470_0333 (
PULPROG	zgpg30
TD	65536
SOLVENT	CDCl3
NS	1024
DS	4
SWH	37037.035 Hz
FIDRES	1.130281 Hz
AQ	0.8847360 sec
RG	101
DW	13.500 usec
DE	6.50 usec
TE	295.2 K
D1	2.00000000 sec
D11	0.03000000 sec
TD0	1
SFO1	125.7804233 MHz
NUC1	13C
P0	3.33 usec
P1	10.00 usec
PLW1	83.14099884 W
SFO2	500.1720007 MHz
NUC2	1H
CPDPRG[2	waltz65
PCPD2	80.00 usec
PLW2	20.93000031 W
PLW12	0.32703000 W
PLW13	0.16449000 W

F2 - Processing parameters

SI	32768
SF	125.7678465 MHz
WDW	EM
SSB	0
LB	1.00 Hz
GB	0
PC	1.40

CH-3
1H_8scan CDCl3 {D:\Spectra} nmr 32

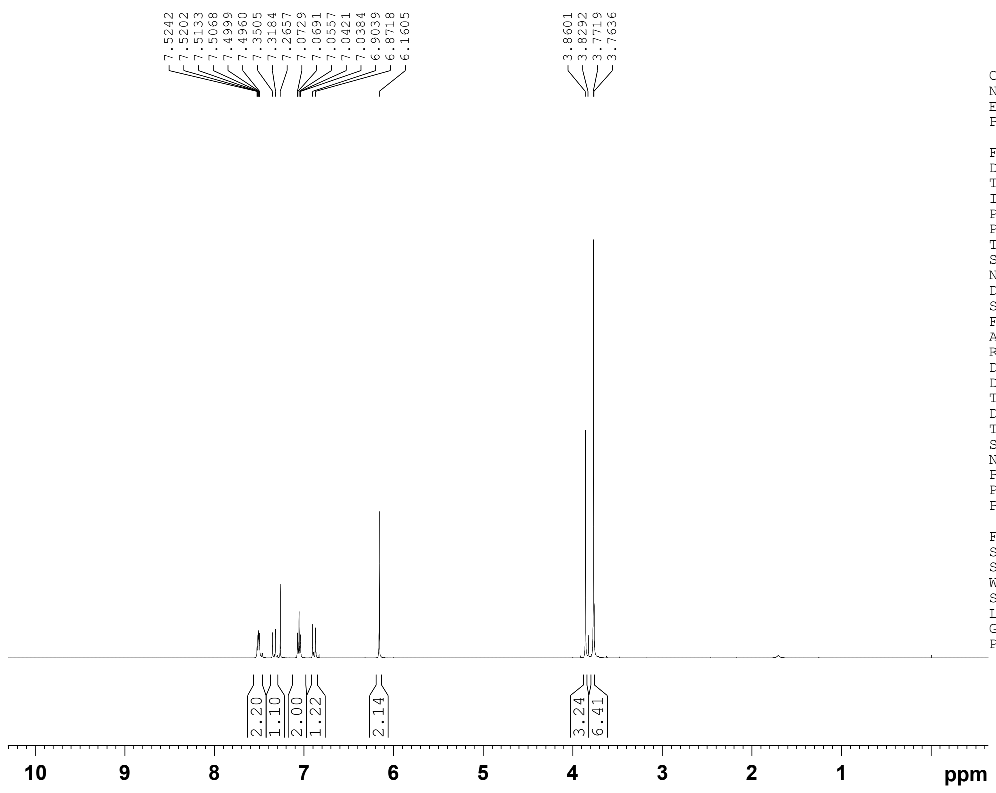

BRUKER
AVANCE NEO
500 MHz NMR
SPECTROMETER
SAIF, P.U.

Current Data Parameters

NAME	Apr01-2021
EXPNO	320
PROCNO	1

F2 - Acquisition Parameters

Date_	20210402
Time	7.17 h
INSTRUM	Avance Neo 500
PROBHD	Z119470_0333 (
PULPROG	zg30
TD	65536
SOLVENT	CDCl3
NS	16
DS	0
SWH	14705.883 Hz
FIDRES	0.448788 Hz
AQ	2.2282240 sec
RG	95.7854
DW	34.000 usec
DE	6.79 usec
TE	294.5 K
D1	1.00000000 sec
TD0	1
SFO1	500.1730885 MHz
NUC1	1H
P0	3.33 usec
P1	10.00 usec
PLW1	20.93000031 W

F2 - Processing parameters

SI	65536
SF	500.1700093 MHz
WDW	EM
SSB	0
LB	0.30 Hz
GB	0
PC	1.00

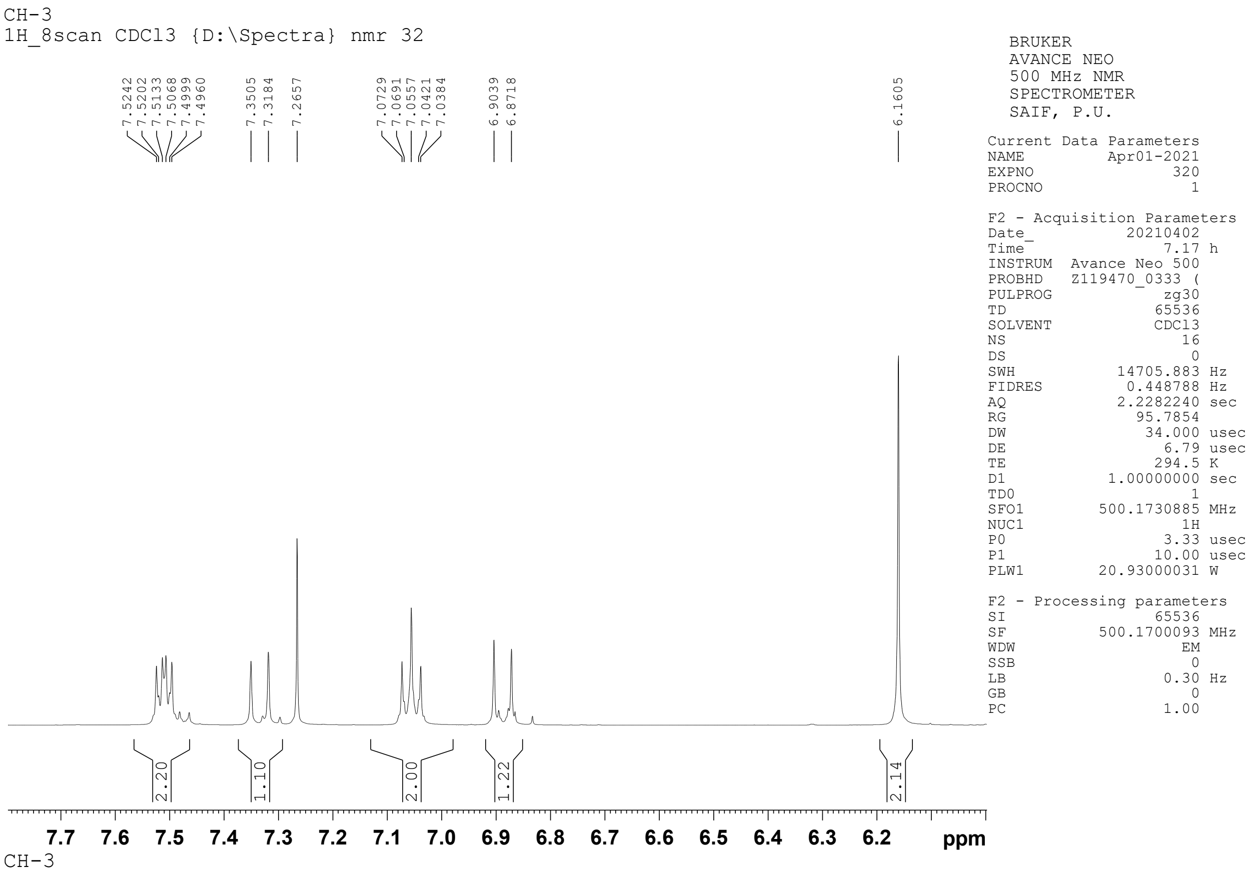
CH-3
1H_8scan CDCl3 {D:\Spectra} nmr 32
7.5242
7.5202
7.5133
7.5068
7.4999
7.4960
7.3505
7.3184
7.2657
7.0729
7.0691
7.0557
7.0421
7.0384
6.9039
6.8718
6.1605
2.20
1.10
2.00
1.22
2.14
7.7 7.6 7.5 7.4 7.3 7.2 7.1 7.0 6.9 6.8 6.7 6.6 6.5 6.4 6.3 6.2 ppm
BRUKER
AVANCE NEO
500 MHz NMR
SPECTROMETER
SAIF, P.U.
Current Data Parameters
NAME Apr01-2021
EXPNO 320
PROCNO 1
F2 - Acquisition Parameters
Date_ 20210402
Time 7.17 h
INSTRUM Avance Neo 500
PROBHD Z119470_0333 (
PULPROG zg30
TD 65536
SOLVENT CDCl3
NS 16
DS 0
SWH 14705.883 Hz
FIDRES 0.448788 Hz
AQ 2.2282240 sec
RG 95.7854
DW 34.000 usec
DE 6.79 usec
TE 294.5 K
D1 1.00000000 sec
TD0 1
SFO1 500.1730885 MHz
NUC1 1H
P0 3.33 usec
P1 10.00 usec
PLW1 20.93000031 W
F2 - Processing parameters
SI 65536
SF 500.1700093 MHz
WDW EM
SSB 0
LB 0.30 Hz
GB 0
PC 1.00

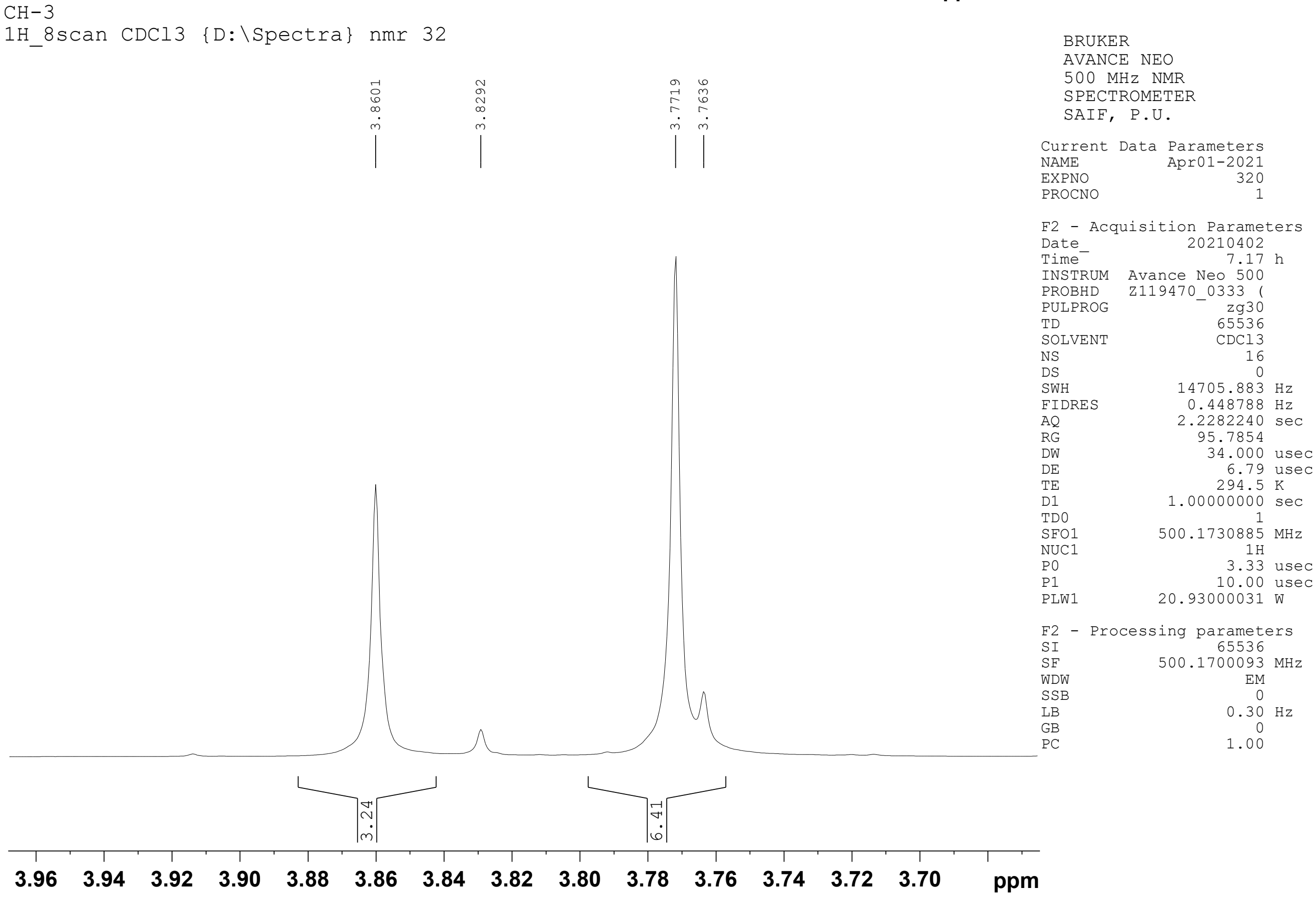
CH-3
1H_8scan CDCl3 {D:\Spectra} nmr 32
3.8601
3.8292
3.7719
3.7636
3.24
6.41
3.96 3.94 3.92 3.90 3.88 3.86 3.84 3.82 3.80 3.78 3.76 3.74 3.72 3.70 ppm
BRUKER
AVANCE NEO
500 MHz NMR
SPECTROMETER
SAIF, P.U.
Current Data Parameters
NAME Apr01-2021
EXPNO 320
PROCNO 1
F2 - Acquisition Parameters
Date_ 20210402
Time 7.17 h
INSTRUM Avance Neo 500
PROBHD Z119470_0333 (
PULPROG zg30
TD 65536
SOLVENT CDCl3
NS 16
DS 0
SWH 14705.883 Hz
FIDRES 0.448788 Hz
AQ 2.2282240 sec
RG 95.7854
DW 34.000 usec
DE 6.79 usec
TE 294.5 K
D1 1.00000000 sec
TD0 1
SFO1 500.1730885 MHz
NUC1 1H
P0 3.33 usec
P1 10.00 usec
PLW1 20.93000031 W
F2 - Processing parameters
SI 65536
SF 500.1700093 MHz
WDW EM
SSB 0
LB 0.30 Hz
GB 0
PC 1.00

CH-3
C13CPD CDCl3 {D:\Spectra} nmr 32

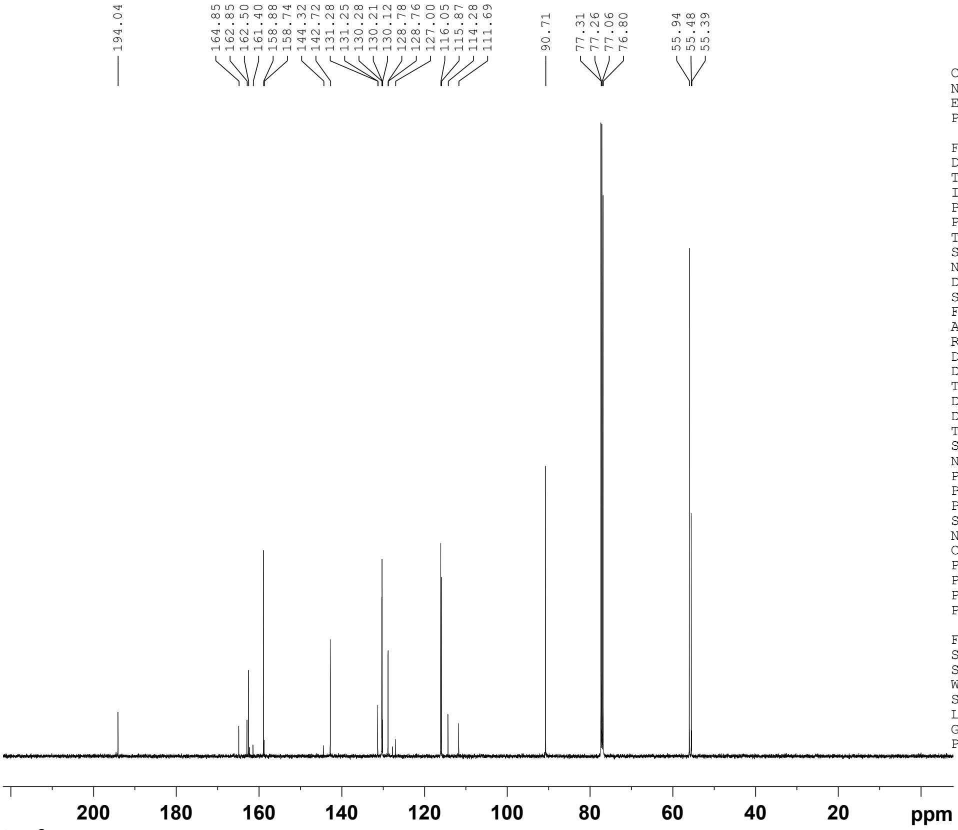

BRUKER
AVANCE NEO
500 MHz NMR SPECTROMETER
SAIF, PANJAB UNIVERSITY,
CHANDIGARH

Current Data Parameters
NAME Apr01-2021
EXPNO 321
PROCNO 1

F2 - Acquisition Parameters
Date_ 20210402
Time 8.08 h
INSTRUM Avance Neo 500
PROBHD Z119470_0333 (
PULPROG zgpg30
TD 65536
SOLVENT CDCl3
NS 1024
DS 4
SWH 37037.035 Hz
FIDRES 1.130281 Hz
AQ 0.8847360 sec
RG 101
DW 13.500 usec
DE 6.50 usec
TE 294.2 K
D1 2.00000000 sec
D11 0.03000000 sec
TD0 1
SFO1 125.7804233 MHz
NUC1 13C
P0 3.33 usec
P1 10.00 usec
PLW1 83.14099884 W
SFO2 500.1720007 MHz
NUC2 1H
CPDPRG[2 waltz65
PCPD2 80.00 usec
PLW2 20.93000031 W
PLW12 0.32703000 W
PLW13 0.16449000 W

F2 - Processing parameters
SI 32768
SF 125.7678465 MHz
WDW EM
SSB 0
LB 1.00 Hz
GB 0
PC 1.40

CH-3
C13CPD CDCl3 {D:\Spectra} nmr 32

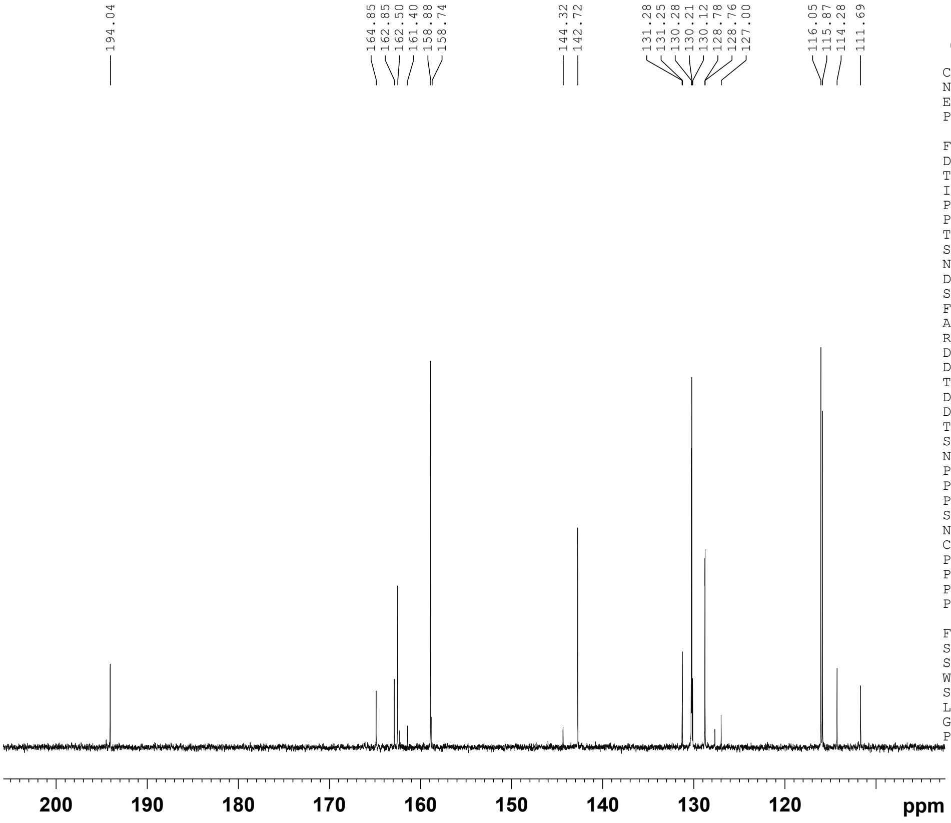

BRUKER
AVANCE NEO
500 MHz NMR SPECTROMETER
SAIF, PANJAB UNIVERSITY,
CHANDIGARH

Current Data Parameters
NAME Apr01-2021
EXPNO 321
PROCNO 1

F2 - Acquisition Parameters
Date_ 20210402
Time 8.08 h
INSTRUM Avance Neo 500
PROBHD Z119470_0333 (
PULPROG zgpg30
TD 65536
SOLVENT CDCl3
NS 1024
DS 4
SWH 37037.035 Hz
FIDRES 1.130281 Hz
AQ 0.8847360 sec
RG 101
DW 13.500 usec
DE 6.50 usec
TE 294.2 K
D1 2.00000000 sec
D11 0.03000000 sec
TD0 1
SFO1 125.7804233 MHz
NUC1 13C
P0 3.33 usec
P1 10.00 usec
PLW1 83.14099884 W
SFO2 500.1720007 MHz
NUC2 1H
CPDPRG[2 waltz65
PCPD2 80.00 usec
PLW2 20.93000031 W
PLW12 0.32703000 W
PLW13 0.16449000 W

F2 - Processing parameters
SI 32768
SF 125.7678465 MHz
WDW EM
SSB 0
LB 1.00 Hz
GB 0
PC 1.40

CH-3
C13CPD CDCl3 {D:\Spectra} nmr 32

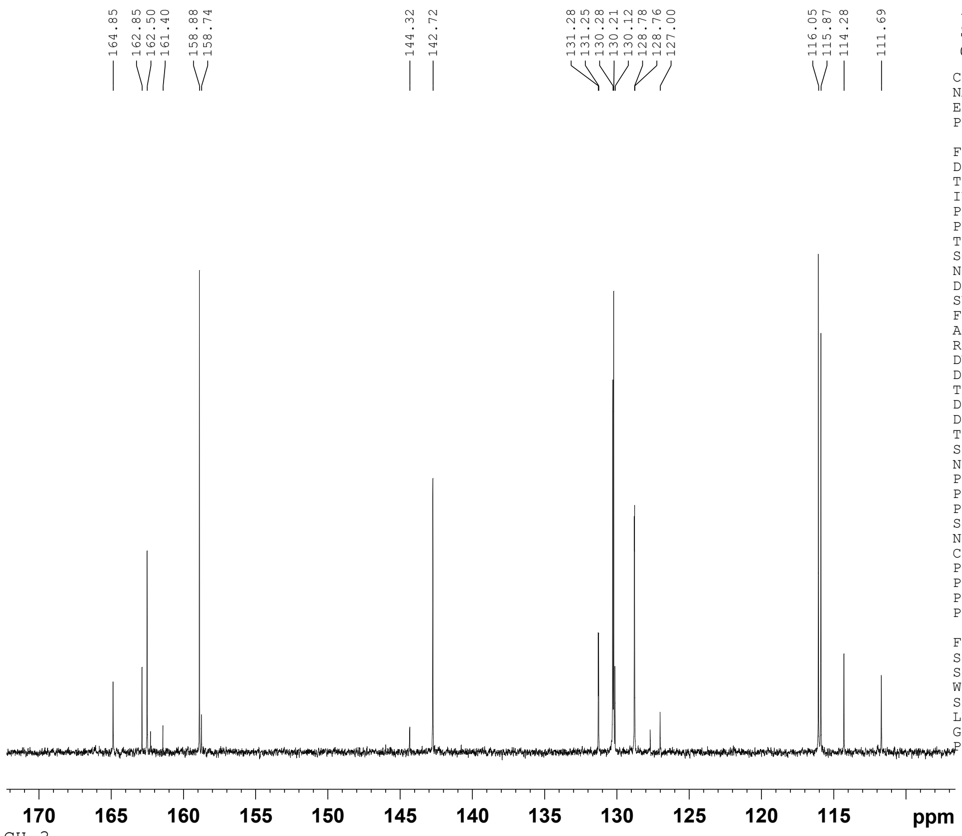

BRUKER
AVANCE NEO
500 MHz NMR SPECTROMETER
SAIF, PANJAB UNIVERSITY,
CHANDIGARH

Current Data Parameters
NAME Apr01-2021
EXPNO 321
PROCNO 1

F2 - Acquisition Parameters
Date_ 20210402
Time 8.08 h
INSTRUM Avance Neo 500
PROBHD Z119470_0333 (
PULPROG zgpg30
TD 65536
SOLVENT CDCl3
NS 1024
DS 4
SWH 37037.035 Hz
FIDRES 1.130281 Hz
AQ 0.8847360 sec
RG 101
DW 13.500 usec
DE 6.50 usec
TE 294.2 K
D1 2.00000000 sec
D11 0.03000000 sec
TD0 1
SFO1 125.7804233 MHz
NUC1 13C
P0 3.33 usec
P1 10.00 usec
PLW1 83.14099884 W
SFO2 500.1720007 MHz
NUC2 1H
CPDPRG[2 waltz65
PCPD2 80.00 usec
PLW2 20.93000031 W
PLW12 0.32703000 W
PLW13 0.16449000 W

F2 - Processing parameters
SI 32768
SF 125.7678465 MHz
WDW EM
SSB 0
LB 1.00 Hz
GB 0
PC 1.40

CH-3
C13CPD CDCl3 {D:\Spectra} nmr 32

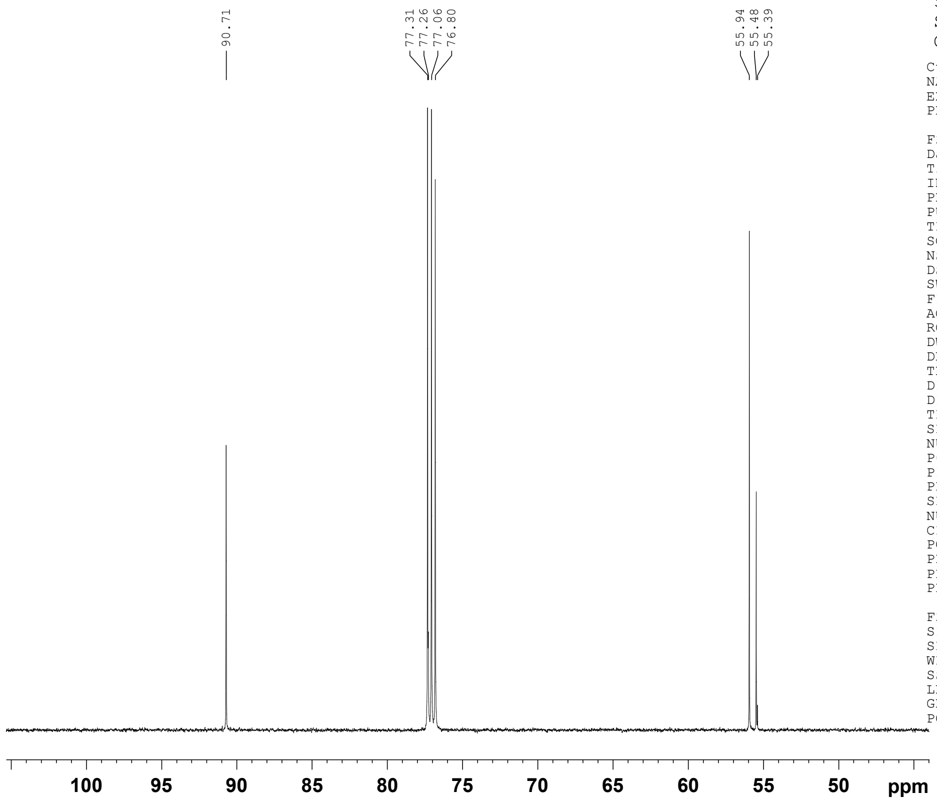

BRUKER
AVANCE NEO
500 MHz NMR SPECTROMETER
SAIF, PANJAB UNIVERSITY,
CHANDIGARH

Current Data Parameters
NAME Apr01-2021
EXPNO 321
PROCNO 1

F2 - Acquisition Parameters
Date_ 20210402
Time 8.08 h
INSTRUM Avance Neo 500
PROBHD Z119470_0333 (
PULPROG zgpg30
TD 65536
SOLVENT CDCl3
NS 1024
DS 4
SWH 37037.035 Hz
FIDRES 1.130281 Hz
AQ 0.8847360 sec
RG 101
DW 13.500 usec
DE 6.50 usec
TE 294.2 K
D1 2.00000000 sec
D11 0.03000000 sec
TD0 1
SFO1 125.7804233 MHz
NUC1 13C
P0 3.33 usec
P1 10.00 usec
PLW1 83.14099884 W
SFO2 500.1720007 MHz
NUC2 1H
CPDPRG[2 waltz65
PCPD2 80.00 usec
PLW2 20.93000031 W
PLW12 0.32703000 W
PLW13 0.16449000 W

F2 - Processing parameters
SI 32768
SF 125.7678465 MHz
WDW EM
SSB 0
LB 1.00 Hz
GB 0
PC 1.40

CH-3
C13CPD CDCl3 {D:\Spectra} nmr 32

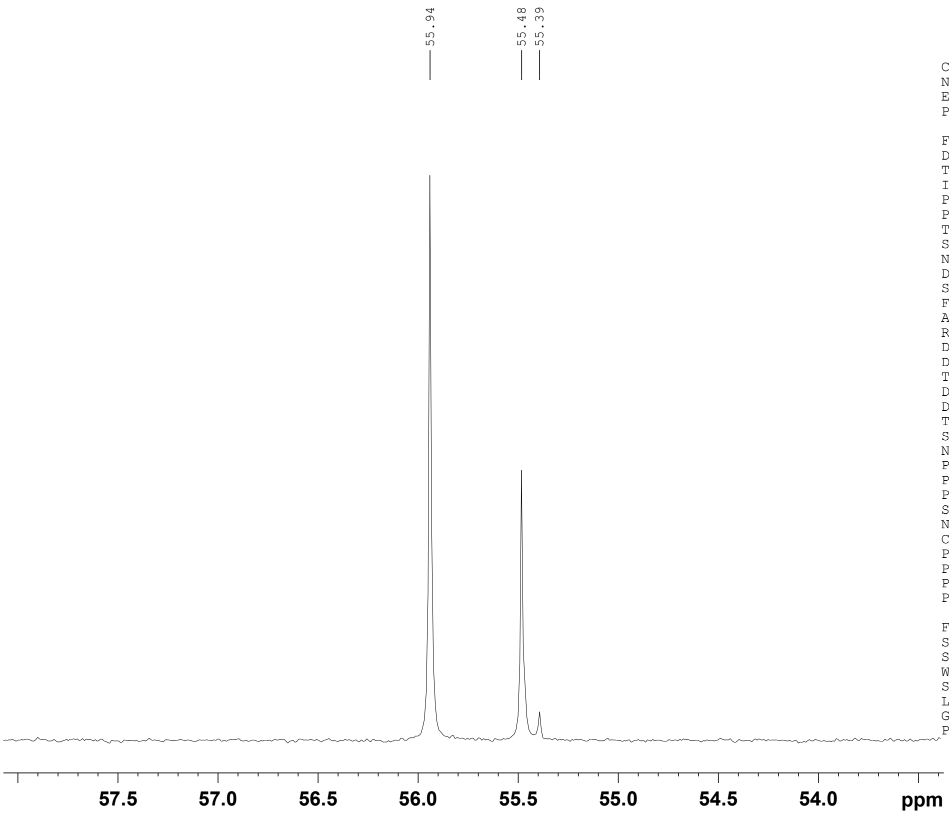

BRUKER
AVANCE NEO
500 MHz NMR SPECTROMETER
SAIF, PANJAB UNIVERSITY,
CHANDIGARH

```
Current Data Parameters
NAME          Apr01-2021
EXPNO                321
PROCNO                 1

F2 - Acquisition Parameters
Date_           20210402
Time                8.08 h
INSTRUM   Avance Neo 500
PROBHD   Z119470_0333 (
PULPROG          zgpg30
TD                 65536
SOLVENT            CDCl3
NS                  1024
DS                     4
SWH            37037.035 Hz
FIDRES          1.130281 Hz
AQ             0.8847360 sec
RG                   101
DW                13.500 usec
DE                  6.50 usec
TE                 294.2 K
D1            2.00000000 sec
D11           0.03000000 sec
TD0                    1
SFO1         125.7804233 MHz
NUC1                 13C
P0                  3.33 usec
P1                 10.00 usec
PLW1         83.14099884 W
SFO2         500.1720007 MHz
NUC2                  1H
CPDPRG[2         waltz65
PCPD2              80.00 usec
PLW2         20.93000031 W
PLW12         0.32703000 W
PLW13         0.16449000 W

F2 - Processing parameters
SI                 32768
SF           125.7678465 MHz
WDW                   EM
SSB                    0
LB                  1.00 Hz
GB                     0
PC                  1.40
```

CH-4
1H_8scan CDCl3 {D:\Spectra} nmr 33

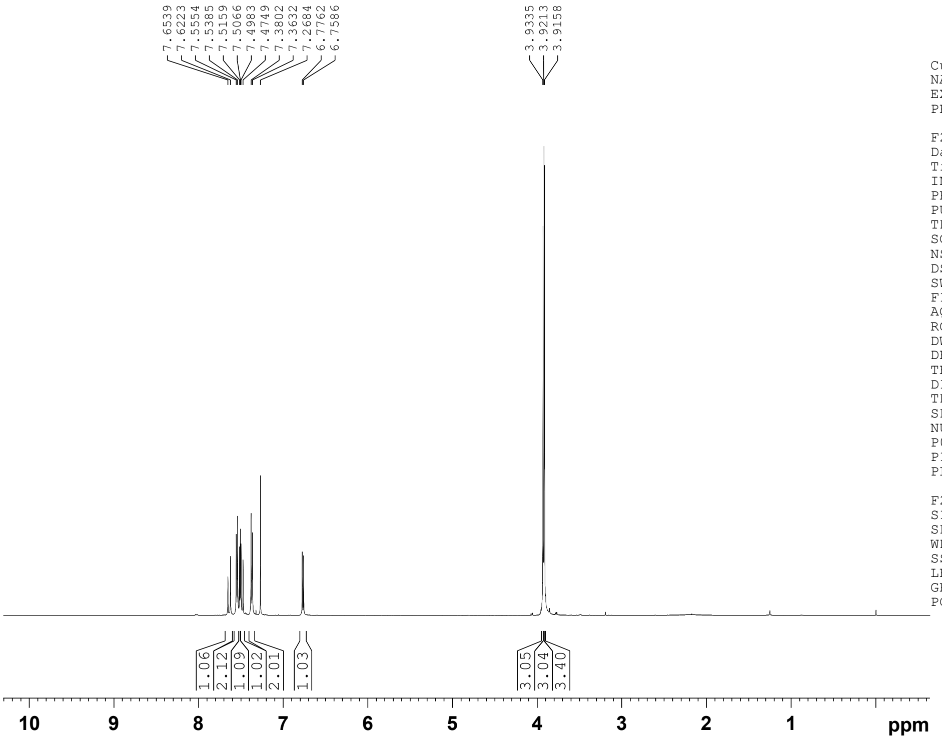

BRUKER
AVANCE NEO
500 MHz NMR
SPECTROMETER
SAIF, P.U.

```
Current Data Parameters
NAME          Apr01-2021
EXPNO                330
PROCNO                 1

F2 - Acquisition Parameters
Date_           20210402
Time                8.11 h
INSTRUM   Avance Neo 500
PROBHD   Z119470_0333 (
PULPROG             zg30
TD                 65536
SOLVENT            CDCl3
NS                    16
DS                     0
SWH            14705.883 Hz
FIDRES          0.448788 Hz
AQ             2.2282240 sec
RG               95.7854
DW                34.000 usec
DE                  6.79 usec
TE                 293.4 K
D1            1.00000000 sec
TD0                    1
SFO1         500.1730885 MHz
NUC1                  1H
P0                  3.33 usec
P1                 10.00 usec
PLW1         20.93000031 W

F2 - Processing parameters
SI                 65536
SF           500.1700079 MHz
WDW                   EM
SSB                    0
LB                  0.30 Hz
GB                     0
PC                  1.00
```

CH-4
1H_8scan CDCl3 {D:\Spectra} nmr 33

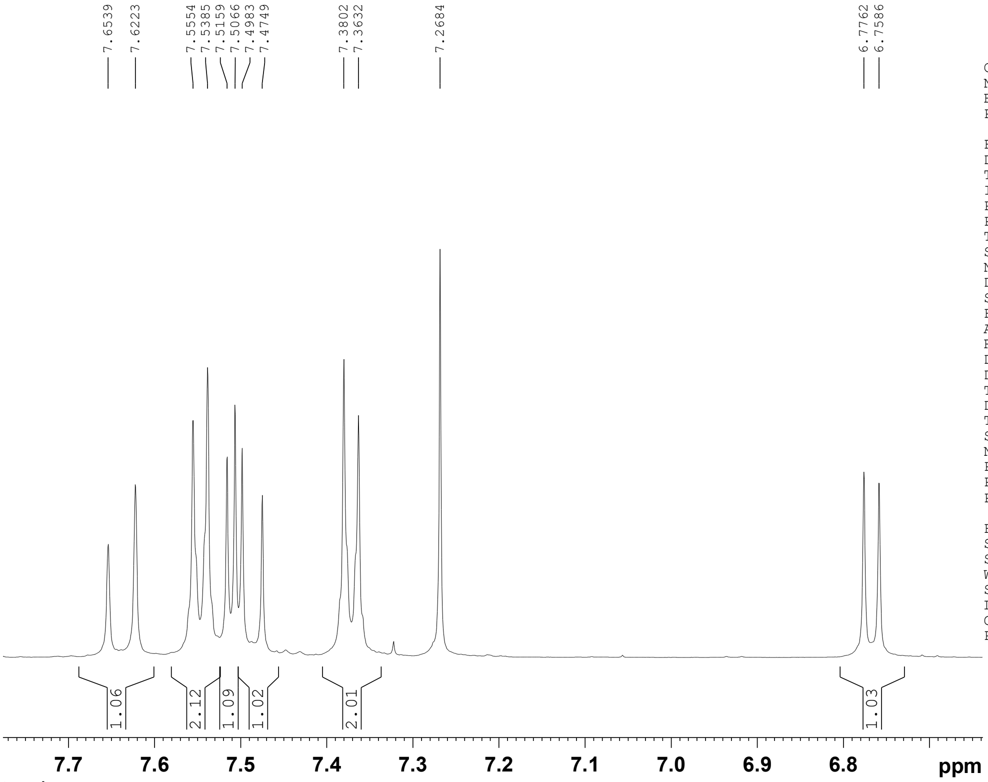

BRUKER
AVANCE NEO
500 MHz NMR
SPECTROMETER
SAIF, P.U.

Current Data Parameters
NAME Apr01-2021
EXPNO 330
PROCNO 1

F2 - Acquisition Parameters
Date_ 20210402
Time 8.11 h
INSTRUM Avance Neo 500
PROBHD Z119470_0333 (
PULPROG zg30
TD 65536
SOLVENT CDCl3
NS 16
DS 0
SWH 14705.883 Hz
FIDRES 0.448788 Hz
AQ 2.2282240 sec
RG 95.7854
DW 34.000 usec
DE 6.79 usec
TE 293.4 K
D1 1.00000000 sec
TD0 1
SFO1 500.1730885 MHz
NUC1 1H
P0 3.33 usec
P1 10.00 usec
PLW1 20.93000031 W

F2 - Processing parameters
SI 65536
SF 500.1700079 MHz
WDW EM
SSB 0
LB 0.30 Hz
GB 0
PC 1.00

CH-4
1H_8scan CDCl3 {D:\Spectra} nmr 33

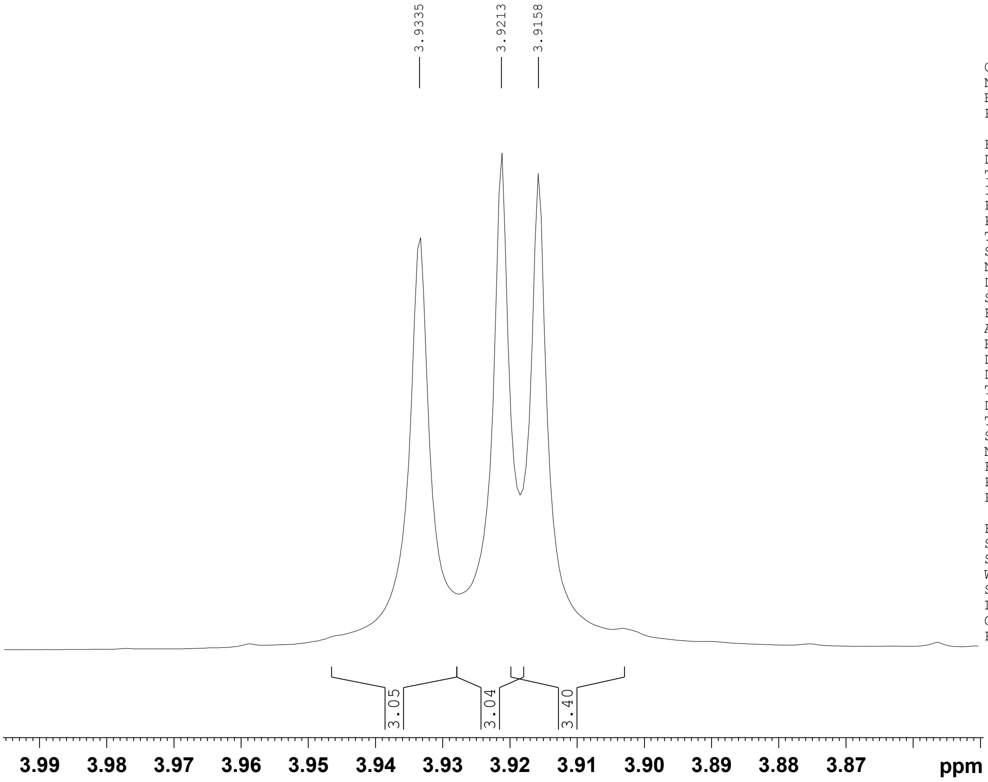

BRUKER
AVANCE NEO
500 MHz NMR
SPECTROMETER
SAIF, P.U.

Current Data Parameters
NAME Apr01-2021
EXPNO 330
PROCNO 1

F2 - Acquisition Parameters
Date_ 20210402
Time 8.11 h
INSTRUM Avance Neo 500
PROBHD Z119470_0333 (
PULPROG zg30
TD 65536
SOLVENT CDCl3
NS 16
DS 0
SWH 14705.883 Hz
FIDRES 0.448788 Hz
AQ 2.2282240 sec
RG 95.7854
DW 34.000 usec
DE 6.79 usec
TE 293.4 K
D1 1.00000000 sec
TD0 1
SFO1 500.1730885 MHz
NUC1 1H
P0 3.33 usec
P1 10.00 usec
PLW1 20.93000031 W

F2 - Processing parameters
SI 65536
SF 500.1700079 MHz
WDW EM
SSB 0
LB 0.30 Hz
GB 0
PC 1.00

CH-4
C13CPD CDCl3 {D:\Spectra} nmr 33

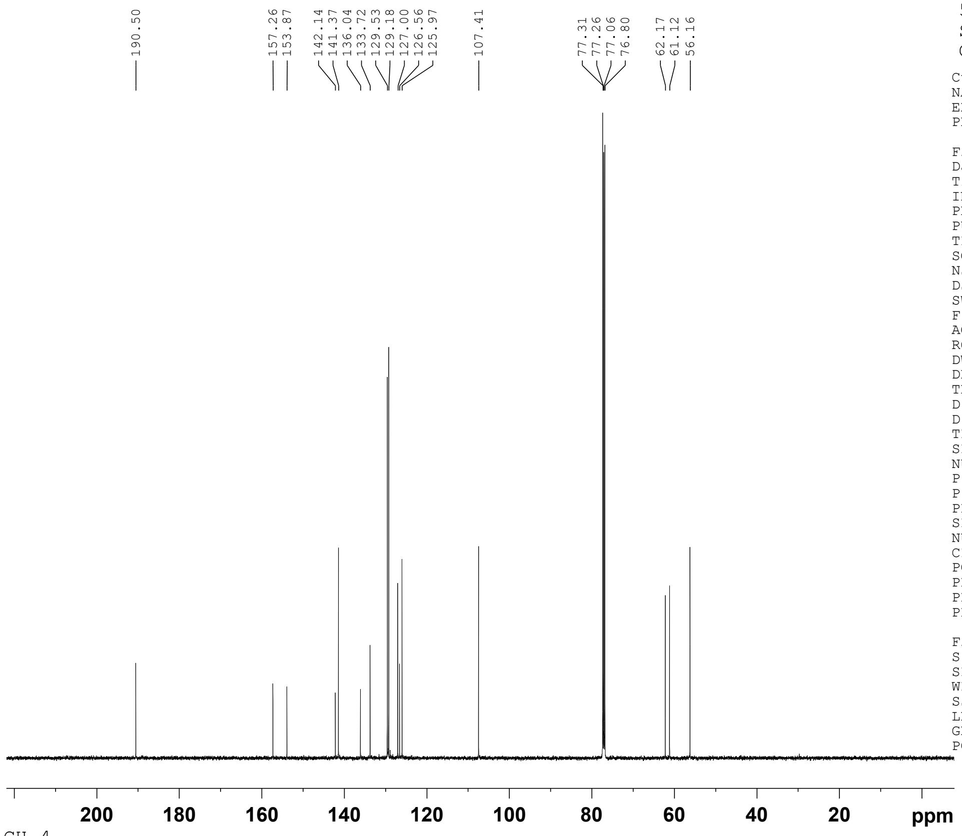

BRUKER
AVANCE NEO
500 MHz NMR SPECTROMETER
SAIF, PANJAB UNIVERSITY,
CHANDIGARH

Current Data Parameters
NAME Apr01-2021
EXPNO 331
PROCNO 1

F2 - Acquisition Parameters
Date_ 20210402
Time 9.01 h
INSTRUM Avance Neo 500
PROBHD Z119470_0333 (
PULPROG zgpg30
TD 65536
SOLVENT CDCl3
NS 1024
DS 4
SWH 37037.035 Hz
FIDRES 1.130281 Hz
AQ 0.8847360 sec
RG 101
DW 13.500 usec
DE 6.50 usec
TE 293.4 K
D1 2.00000000 sec
D11 0.03000000 sec
TD0 1
SFO1 125.7804233 MHz
NUC1 13C
P0 3.33 usec
P1 10.00 usec
PLW1 83.14099884 W
SFO2 500.1720007 MHz
NUC2 1H
CPDPRG[2 waltz65
PCPD2 80.00 usec
PLW2 20.93000031 W
PLW12 0.32703000 W
PLW13 0.16449000 W

F2 - Processing parameters
SI 32768
SF 125.7678465 MHz
WDW EM
SSB 0
LB 1.00 Hz
GB 0
PC 1.40

CH-4
C13CPD CDCl3 {D:\Spectra} nmr 33

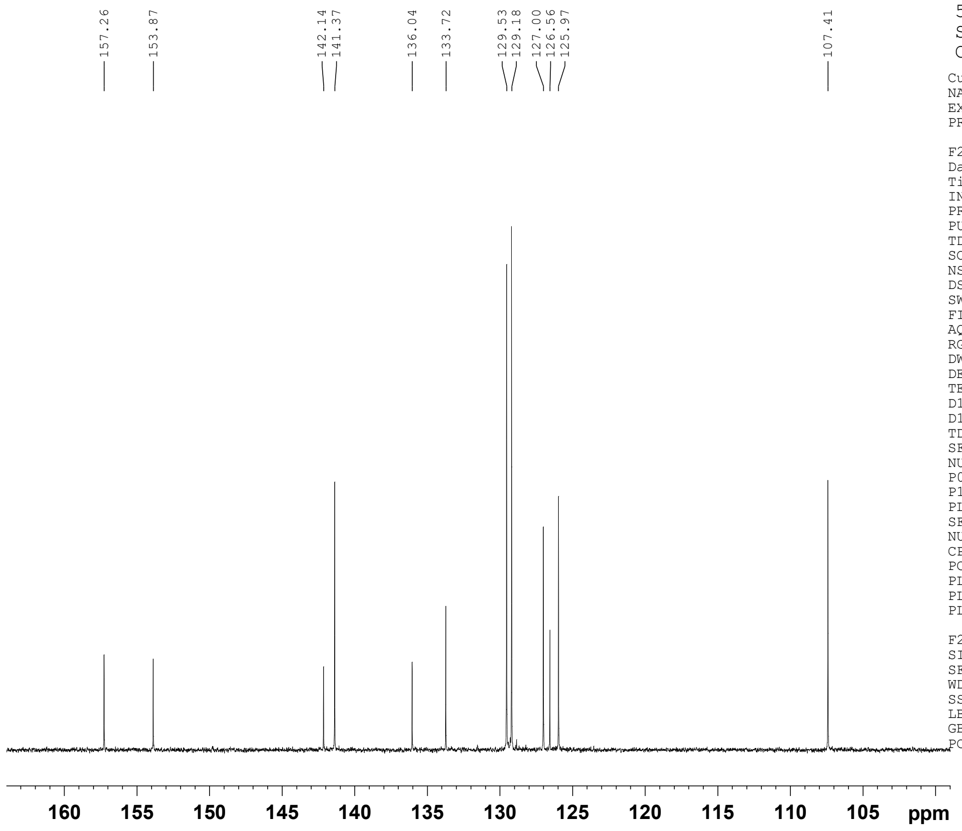

BRUKER
AVANCE NEO
500 MHz NMR SPECTROMETER
SAIF, PANJAB UNIVERSITY,
CHANDIGARH

Current Data Parameters
NAME Apr01-2021
EXPNO 331
PROCNO 1

F2 - Acquisition Parameters
Date_ 20210402
Time 9.01 h
INSTRUM Avance Neo 500
PROBHD Z119470_0333 (
PULPROG zgpg30
TD 65536
SOLVENT CDCl3
NS 1024
DS 4
SWH 37037.035 Hz
FIDRES 1.130281 Hz
AQ 0.8847360 sec
RG 101
DW 13.500 usec
DE 6.50 usec
TE 293.4 K
D1 2.00000000 sec
D11 0.03000000 sec
TD0 1
SFO1 125.7804233 MHz
NUC1 13C
P0 3.33 usec
P1 10.00 usec
PLW1 83.14099884 W
SFO2 500.1720007 MHz
NUC2 1H
CPDPRG[2 waltz65
PCPD2 80.00 usec
PLW2 20.93000031 W
PLW12 0.32703000 W
PLW13 0.16449000 W

F2 - Processing parameters
SI 32768
SF 125.7678465 MHz
WDW EM
SSB 0
LB 1.00 Hz
GB 0
PC 1.40

CH-4
C13CPD CDCl3 {D:\Spectra} nmr 33

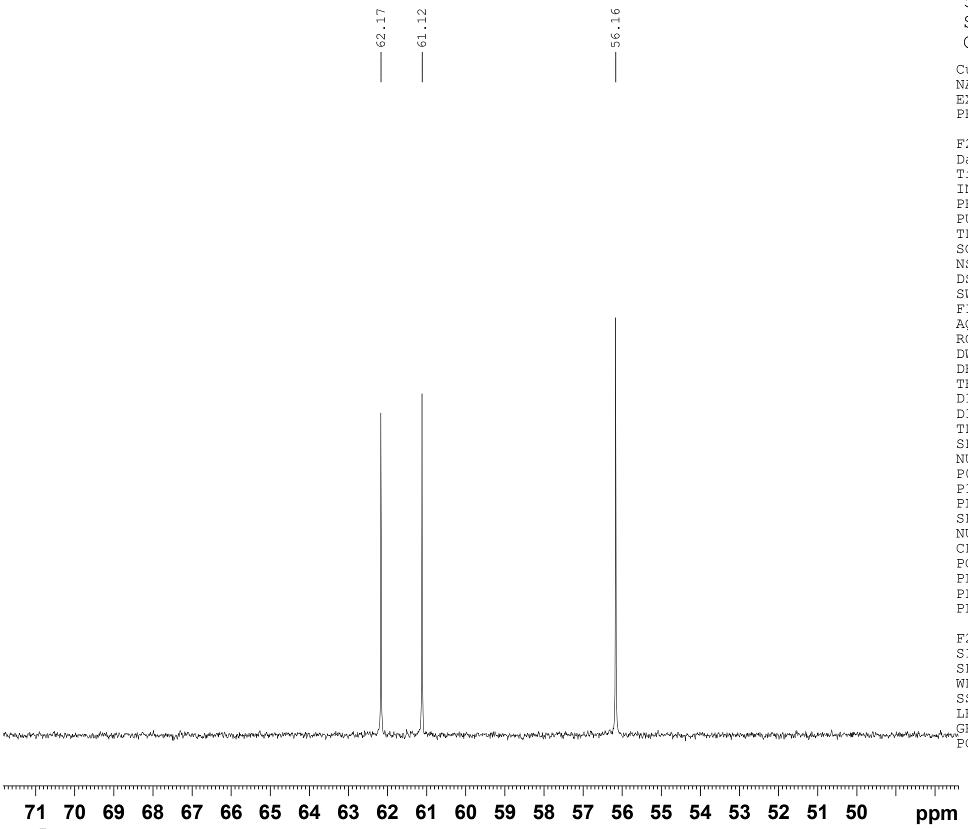

BRUKER
AVANCE NEO
500 MHz NMR SPECTROMETER
SAIF, PANJAB UNIVERSITY,
CHANDIGARH

Current Data Parameters
NAME Apr01-2021
EXPNO 331
PROCNO 1

F2 - Acquisition Parameters
Date_ 20210402
Time 9.01 h
INSTRUM Avance Neo 500
PROBHD Z119470_0333 (
PULPROG zgpg30
TD 65536
SOLVENT CDCl3
NS 1024
DS 4
SWH 37037.035 Hz
FIDRES 1.130281 Hz
AQ 0.8847360 sec
RG 101
DW 13.500 usec
DE 6.50 usec
TE 293.4 K
D1 2.00000000 sec
D11 0.03000000 sec
TD0 1
SFO1 125.7804233 MHz
NUC1 13C
P0 3.33 usec
P1 10.00 usec
PLW1 83.14099884 W
SFO2 500.1720007 MHz
NUC2 1H
CPDPRG[2 waltz65
PCPD2 80.00 usec
PLW2 20.93000031 W
PLW12 0.32703000 W
PLW13 0.16449000 W

F2 - Processing parameters
SI 32768
SF 125.7678465 MHz
WDW EM
SSB 0
LB 1.00 Hz
GB 0
PC 1.40

CH-5
1H_8scan CDCl3 {D:\Spectra} nmr 34

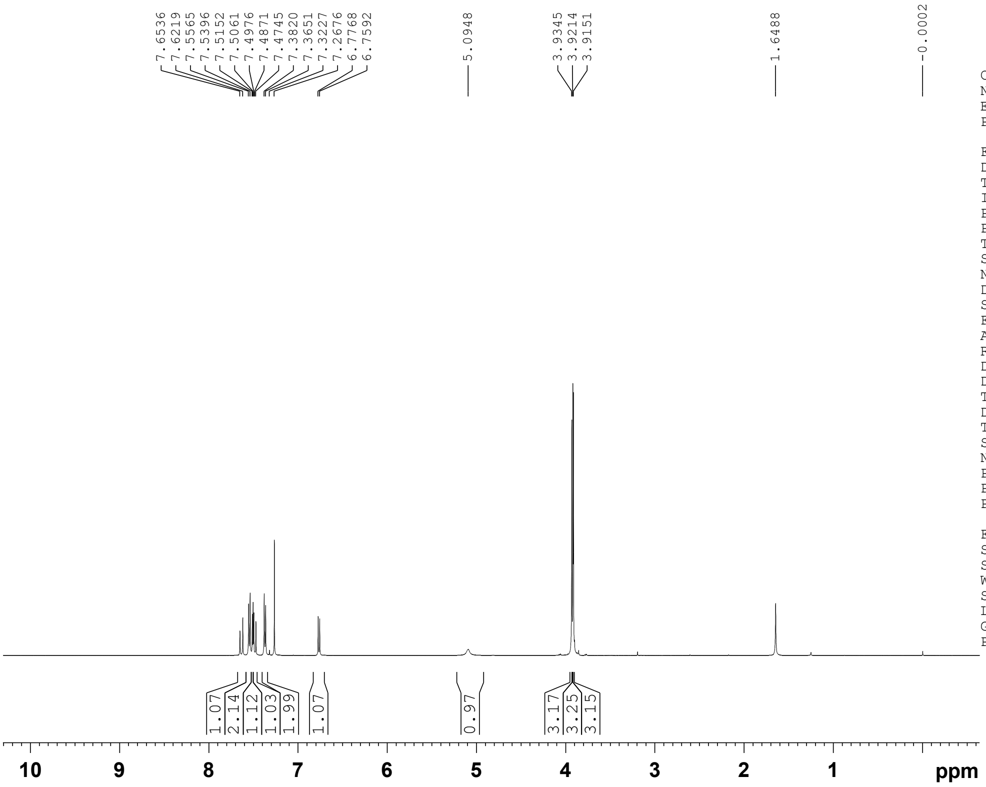

BRUKER
AVANCE NEO
500 MHz NMR
SPECTROMETER
SAIF, P.U.

Current Data Parameters
NAME Apr01-2021
EXPNO 340
PROCNO 1

F2 - Acquisition Parameters
Date_ 20210402
Time 9.04 h
INSTRUM Avance Neo 500
PROBHD Z119470_0333 (
PULPROG zg30
TD 65536
SOLVENT CDCl3
NS 16
DS 0
SWH 14705.883 Hz
FIDRES 0.448788 Hz
AQ 2.2282240 sec
RG 101
DW 34.000 usec
DE 6.79 usec
TE 292.7 K
D1 1.00000000 sec
TD0 1
SFO1 500.1730885 MHz
NUC1 1H
P0 3.33 usec
P1 10.00 usec
PLW1 20.93000031 W

F2 - Processing parameters
SI 65536
SF 500.1700082 MHz
WDW EM
SSB 0
LB 0.30 Hz
GB 0
PC 1.00

CH-5
1H_8scan CDCl3 {D:\Spectra} nmr 34

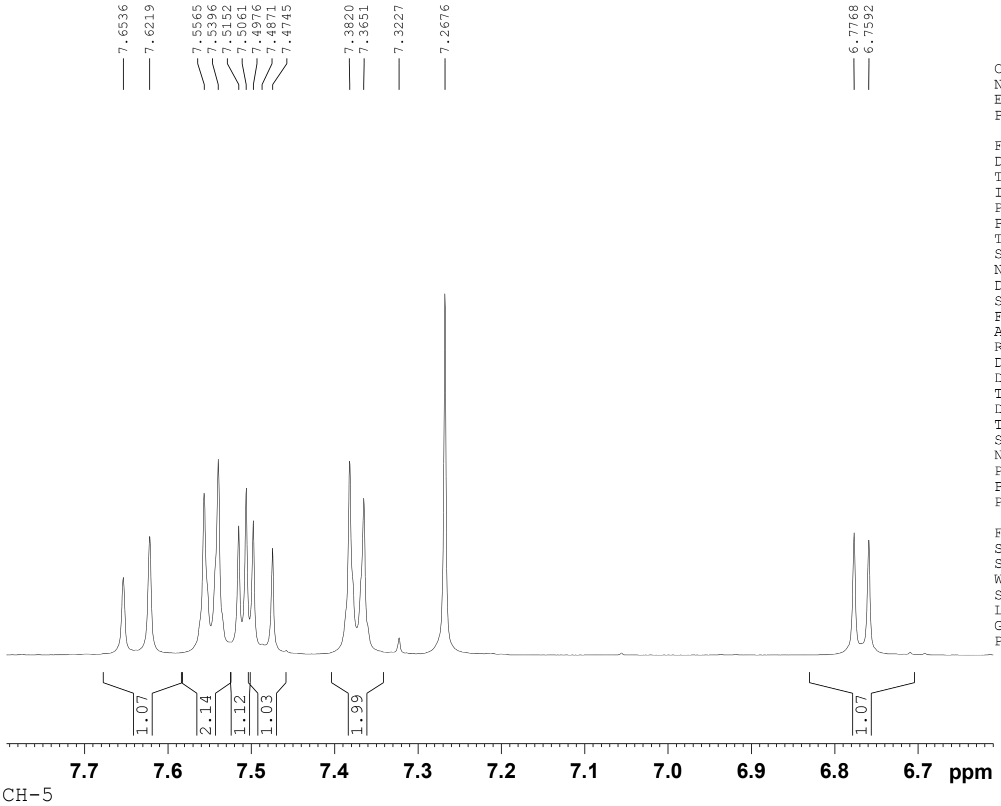

BRUKER
AVANCE NEO
500 MHz NMR
SPECTROMETER
SAIF, P.U.

Current Data Parameters
NAME Apr01-2021
EXPNO 340
PROCNO 1

F2 - Acquisition Parameters
Date_ 20210402
Time 9.04 h
INSTRUM Avance Neo 500
PROBHD Z119470_0333 (
PULPROG zg30
TD 65536
SOLVENT CDCl3
NS 16
DS 0
SWH 14705.883 Hz
FIDRES 0.448788 Hz
AQ 2.2282240 sec
RG 101
DW 34.000 usec
DE 6.79 usec
TE 292.7 K
D1 1.00000000 sec
TD0 1
SFO1 500.1730885 MHz
NUC1 1H
P0 3.33 usec
P1 10.00 usec
PLW1 20.93000031 W

F2 - Processing parameters
SI 65536
SF 500.1700082 MHz
WDW EM
SSB 0
LB 0.30 Hz
GB 0
PC 1.00

CH-5
1H_8scan CDCl3 {D:\Spectra} nmr 34

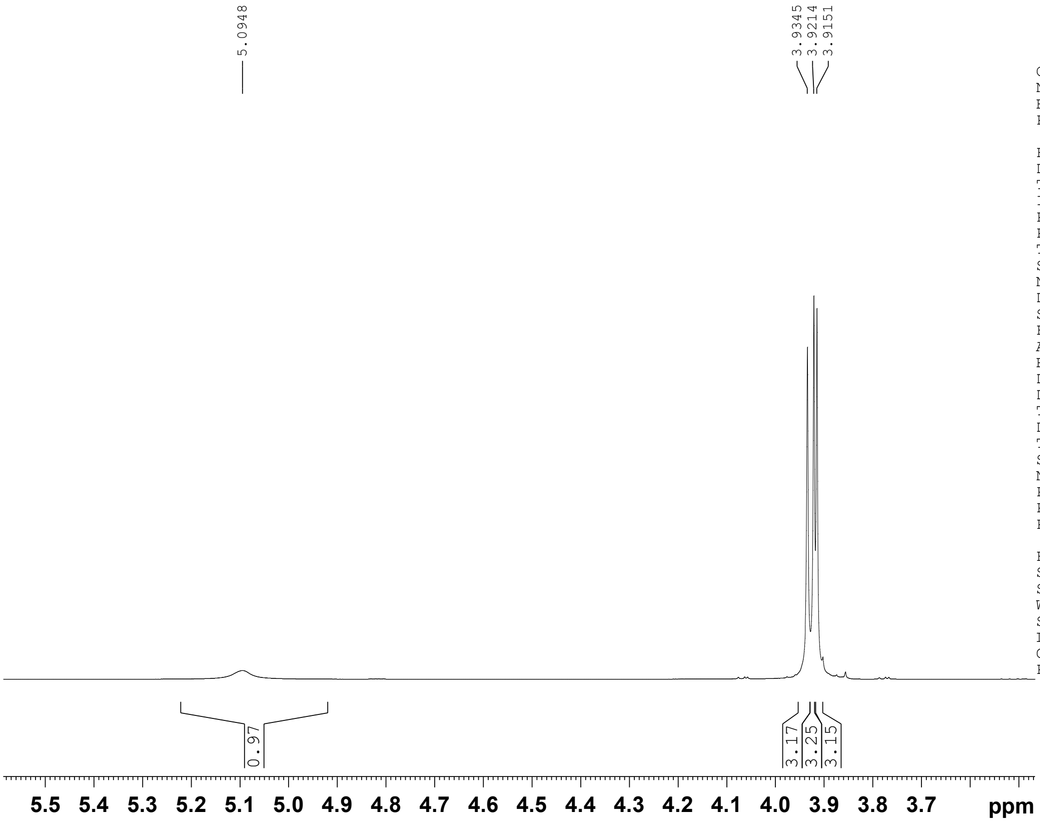

BRUKER
AVANCE NEO
500 MHz NMR
SPECTROMETER
SAIF, P.U.

Current Data Parameters
NAME Apr01-2021
EXPNO 340
PROCNO 1

F2 - Acquisition Parameters
Date_ 20210402
Time 9.04 h
INSTRUM Avance Neo 500
PROBHD Z119470_0333 (
PULPROG zg30
TD 65536
SOLVENT CDCl3
NS 16
DS 0
SWH 14705.883 Hz
FIDRES 0.448788 Hz
AQ 2.2282240 sec
RG 101
DW 34.000 usec
DE 6.79 usec
TE 292.7 K
D1 1.00000000 sec
TD0 1
SFO1 500.1730885 MHz
NUC1 1H
P0 3.33 usec
P1 10.00 usec
PLW1 20.93000031 W

F2 - Processing parameters
SI 65536
SF 500.1700082 MHz
WDW EM
SSB 0
LB 0.30 Hz
GB 0
PC 1.00

CH-5
1H_8scan CDCl3 {D:\Spectra} nmr 34

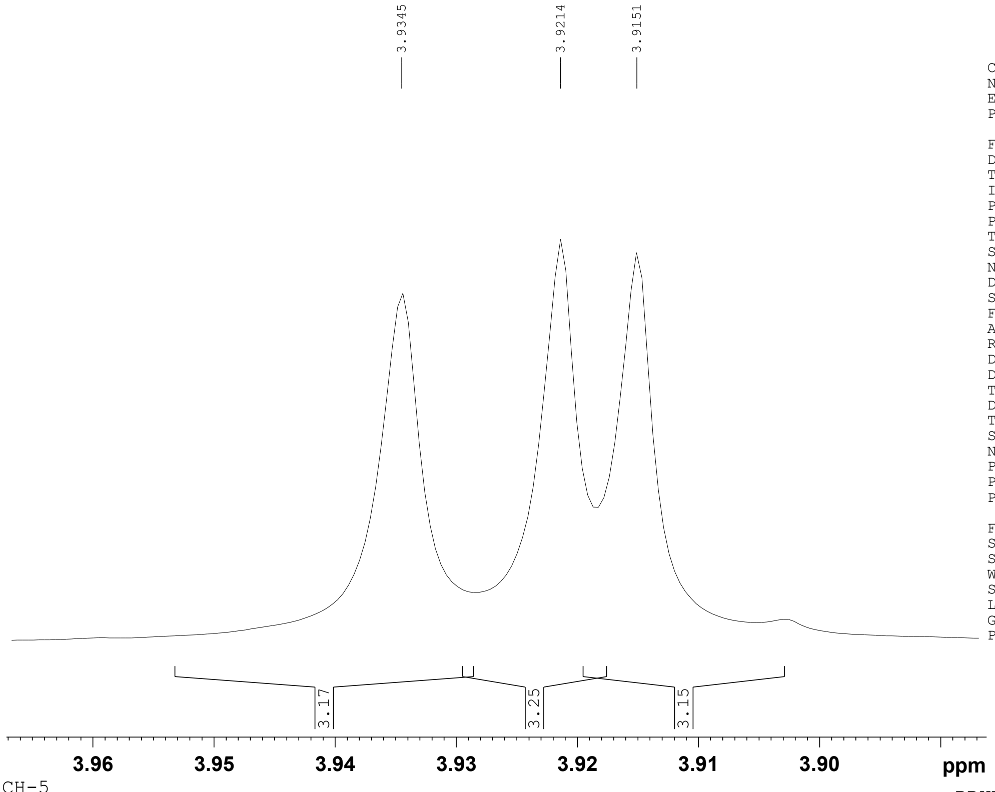

BRUKER
AVANCE NEO
500 MHz NMR
SPECTROMETER
SAIF, P.U.

Current Data Parameters
NAME Apr01-2021
EXPNO 340
PROCNO 1

F2 - Acquisition Parameters
Date_ 20210402
Time 9.04 h
INSTRUM Avance Neo 500
PROBHD Z119470_0333 (
PULPROG zg30
TD 65536
SOLVENT CDCl3
NS 16
DS 0
SWH 14705.883 Hz
FIDRES 0.448788 Hz
AQ 2.2282240 sec
RG 101
DW 34.000 usec
DE 6.79 usec
TE 292.7 K
D1 1.00000000 sec
TD0 1
SFO1 500.1730885 MHz
NUC1 1H
P0 3.33 usec
P1 10.00 usec
PLW1 20.93000031 W

F2 - Processing parameters
SI 65536
SF 500.1700082 MHz
WDW EM
SSB 0
LB 0.30 Hz
GB 0
PC 1.00

CH-5
C13CPD CDCl3 {D:\Spectra} nmr 34

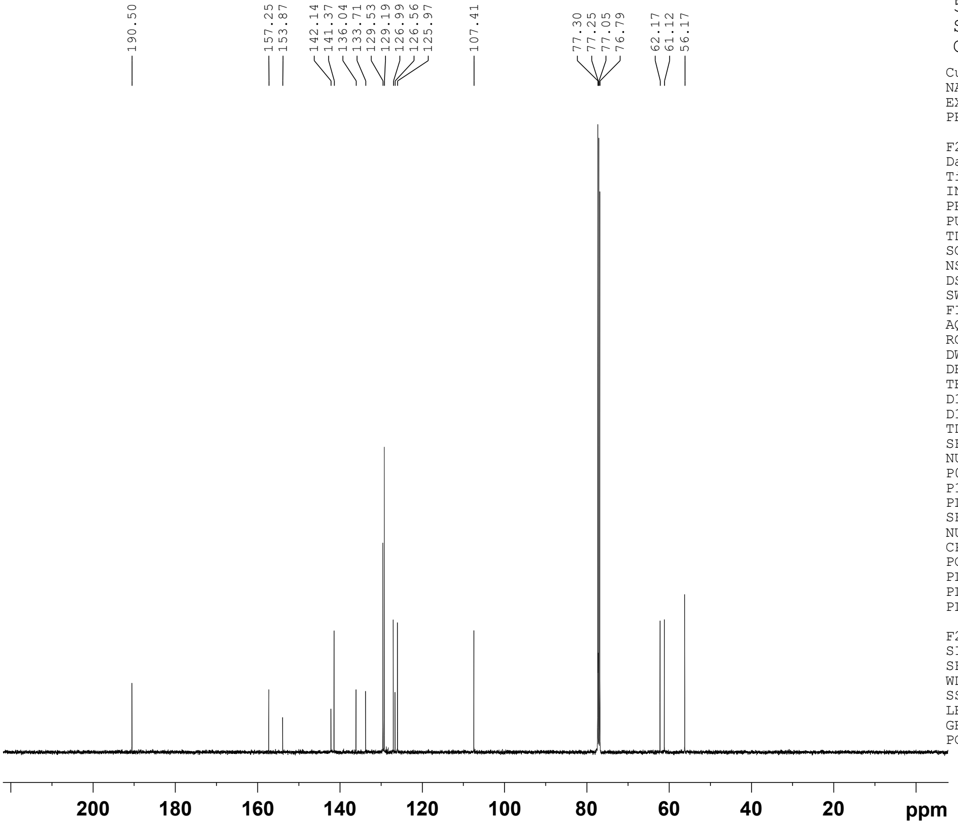

BRUKER
AVANCE NEO
500 MHz NMR SPECTROMETER
SAIF, PANJAB UNIVERSITY,
CHANDIGARH

Current Data Parameters
NAME Apr01-2021
EXPNO 341
PROCNO 1

F2 - Acquisition Parameters
Date_ 20210402
Time 9.55 h
INSTRUM Avance Neo 500
PROBHD Z119470_0333 (
PULPROG zgpg30
TD 65536
SOLVENT CDCl3
NS 1024
DS 4
SWH 37037.035 Hz
FIDRES 1.130281 Hz
AQ 0.8847360 sec
RG 101
DW 13.500 usec
DE 6.50 usec
TE 293.1 K
D1 2.00000000 sec
D11 0.03000000 sec
TD0 1
SFO1 125.7804233 MHz
NUC1 13C
P0 3.33 usec
P1 10.00 usec
PLW1 83.14099884 W
SFO2 500.1720007 MHz
NUC2 1H
CPDPRG[2 waltz65
PCPD2 80.00 usec
PLW2 20.93000031 W
PLW12 0.32703000 W
PLW13 0.16449000 W

F2 - Processing parameters
SI 32768
SF 125.7678465 MHz
WDW EM
SSB 0
LB 1.00 Hz
GB 0
PC 1.40

CH-5
C13CPD CDCl3 {D:\Spectra} nmr 34

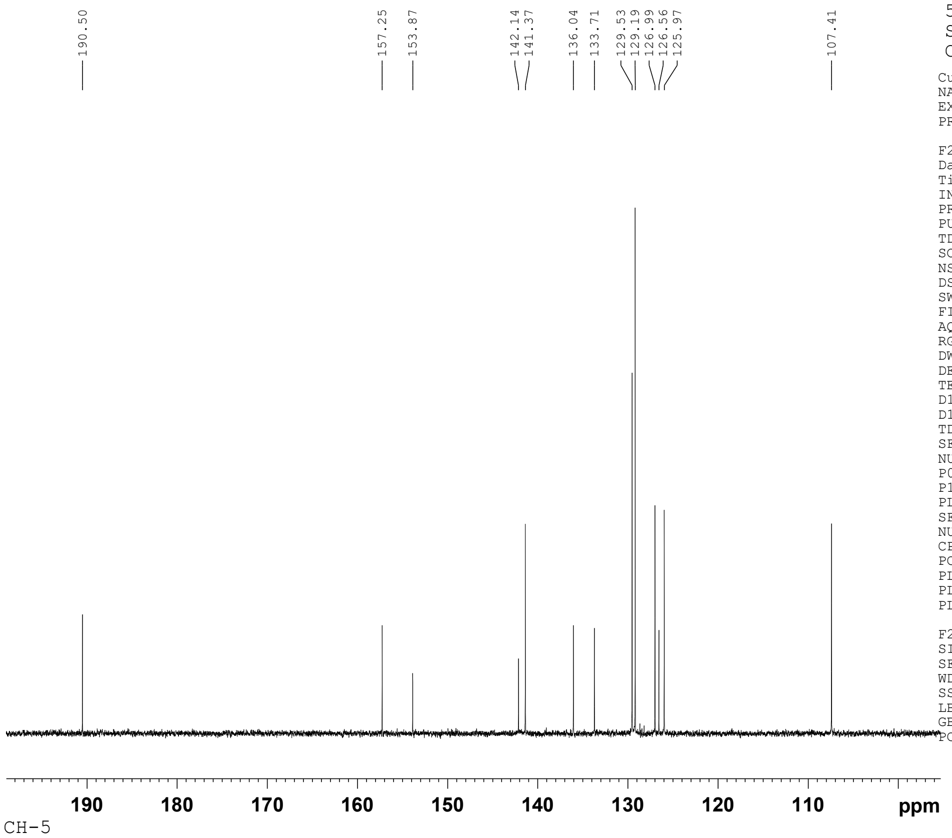

BRUKER
AVANCE NEO
500 MHz NMR SPECTROMETER
SAIF, PANJAB UNIVERSITY,
CHANDIGARH

Current Data Parameters
NAME Apr01-2021
EXPNO 341
PROCNO 1

F2 - Acquisition Parameters
Date_ 20210402
Time 9.55 h
INSTRUM Avance Neo 500
PROBHD Z119470_0333 (
PULPROG zgpg30
TD 65536
SOLVENT CDCl3
NS 1024
DS 4
SWH 37037.035 Hz
FIDRES 1.130281 Hz
AQ 0.8847360 sec
RG 101
DW 13.500 usec
DE 6.50 usec
TE 293.1 K
D1 2.00000000 sec
D11 0.03000000 sec
TD0 1
SFO1 125.7804233 MHz
NUC1 13C
P0 3.33 usec
P1 10.00 usec
PLW1 83.14099884 W
SFO2 500.1720007 MHz
NUC2 1H
CPDPRG[2 waltz65
PCPD2 80.00 usec
PLW2 20.93000031 W
PLW12 0.32703000 W
PLW13 0.16449000 W

F2 - Processing parameters
SI 32768
SF 125.7678465 MHz
WDW EM
SSB 0
LB 1.00 Hz
GB 0
PC 1.40

CH-5
C13CPD CDCl3 {D:\Spectra} nmr 34

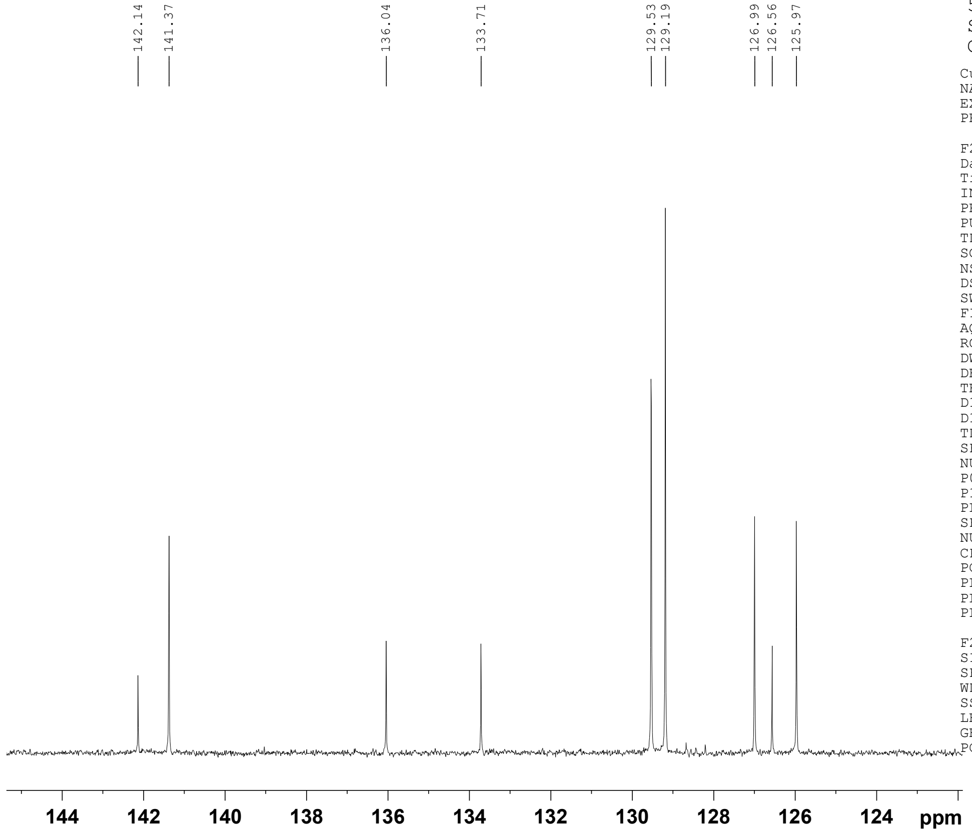

BRUKER
AVANCE NEO
500 MHz NMR SPECTROMETER
SAIF, PANJAB UNIVERSITY,
CHANDIGARH

Current Data Parameters
NAME Apr01-2021
EXPNO 341
PROCNO 1

F2 - Acquisition Parameters
Date_ 20210402
Time 9.55 h
INSTRUM Avance Neo 500
PROBHD Z119470_0333 (
PULPROG zgpg30
TD 65536
SOLVENT CDCl3
NS 1024
DS 4
SWH 37037.035 Hz
FIDRES 1.130281 Hz
AQ 0.8847360 sec
RG 101
DW 13.500 usec
DE 6.50 usec
TE 293.1 K
D1 2.00000000 sec
D11 0.03000000 sec
TD0 1
SFO1 125.7804233 MHz
NUC1 13C
P0 3.33 usec
P1 10.00 usec
PLW1 83.14099884 W
SFO2 500.1720007 MHz
NUC2 1H
CPDPRG[2 waltz65
PCPD2 80.00 usec
PLW2 20.93000031 W
PLW12 0.32703000 W
PLW13 0.16449000 W

F2 - Processing parameters
SI 32768
SF 125.7678465 MHz
WDW EM
SSB 0
LB 1.00 Hz
GB 0
PC 1.40

CH-5
C13CPD CDCl3 {D:\Spectra} nmr 34

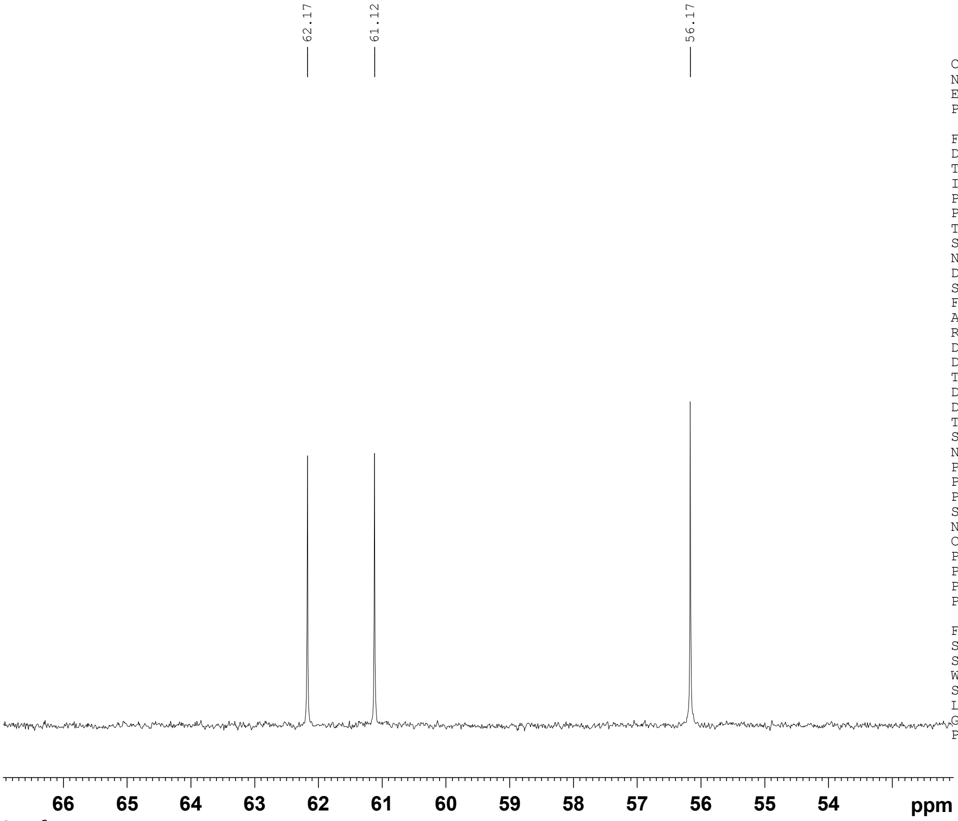

BRUKER
AVANCE NEO
500 MHz NMR SPECTROMETER
SAIF, PANJAB UNIVERSITY,
CHANDIGARH

Current Data Parameters

NAME	Apr01-2021	
EXPNO	341	
PROCNO	1	

F2 - Acquisition Parameters

Date_	20210402	
Time	9.55	h
INSTRUM	Avance Neo 500	
PROBHD	Z119470_0333 (	
PULPROG	zgpg30	
TD	65536	
SOLVENT	CDCl3	
NS	1024	
DS	4	
SWH	37037.035	Hz
FIDRES	1.130281	Hz
AQ	0.8847360	sec
RG	101	
DW	13.500	usec
DE	6.50	usec
TE	293.1	K
D1	2.00000000	sec
D11	0.03000000	sec
TD0	1	
SFO1	125.7804233	MHz
NUC1	13C	
P0	3.33	usec
P1	10.00	usec
PLW1	83.14099884	W
SFO2	500.1720007	MHz
NUC2	1H	
CPDPRG[2	waltz65	
PCPD2	80.00	usec
PLW2	20.93000031	W
PLW12	0.32703000	W
PLW13	0.16449000	W

F2 - Processing parameters

SI	32768	
SF	125.7678465	MHz
WDW	EM	
SSB	0	
LB	1.00	Hz
GB	0	
PC	1.40	

CH-6
1H_8scan CDCl3 {D:\Spectra} nmr 35

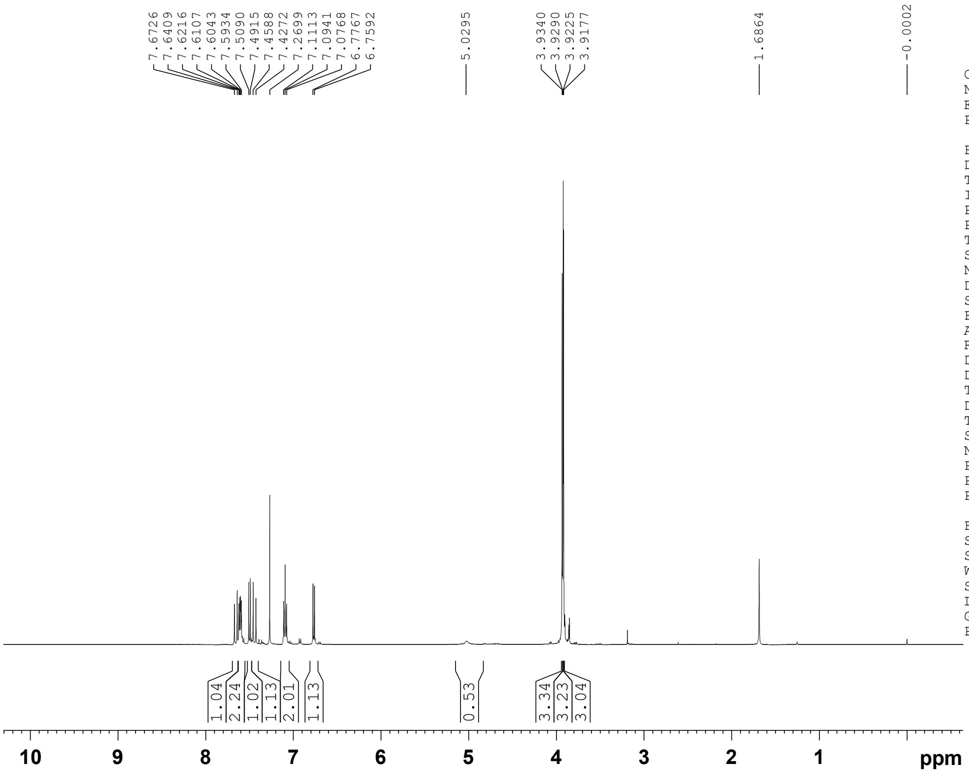

BRUKER
AVANCE NEO
500 MHz NMR
SPECTROMETER
SAIF, P.U.

Current Data Parameters

NAME	Apr01-2021	
EXPNO	350	
PROCNO	1	

F2 - Acquisition Parameters

Date_	20210402	
Time	9.57	h
INSTRUM	Avance Neo 500	
PROBHD	Z119470_0333 (	
PULPROG	zg30	
TD	65536	
SOLVENT	CDCl3	
NS	16	
DS	0	
SWH	14705.883	Hz
FIDRES	0.448788	Hz
AQ	2.2282240	sec
RG	95.7854	
DW	34.000	usec
DE	6.79	usec
TE	292.3	K
D1	1.00000000	sec
TD0	1	
SFO1	500.1730885	MHz
NUC1	1H	
P0	3.33	usec
P1	10.00	usec
PLW1	20.93000031	W

F2 - Processing parameters

SI	65536	
SF	500.1700071	MHz
WDW	EM	
SSB	0	
LB	0.30	Hz
GB	0	
PC	1.00	

CH-6
1H_8scan CDCl3 {D:\Spectra} nmr 35

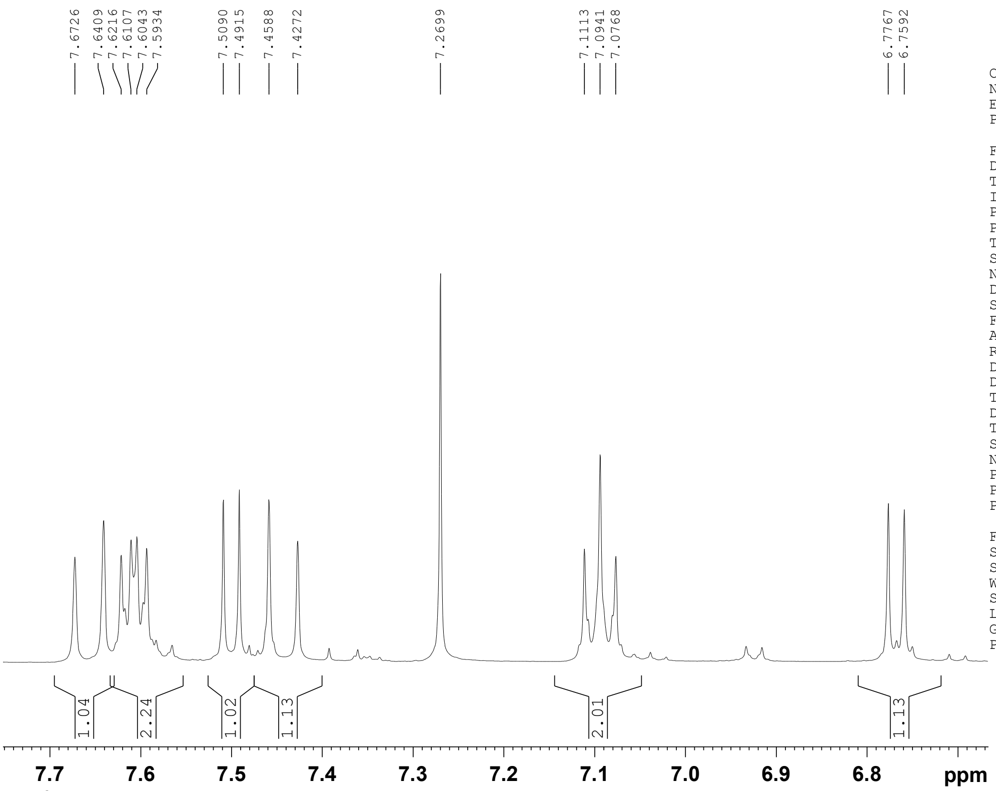

BRUKER
AVANCE NEO
500 MHz NMR
SPECTROMETER
SAIF, P.U.

Current Data Parameters
NAME Apr01-2021
EXPNO 350
PROCNO 1

F2 - Acquisition Parameters
Date_ 20210402
Time 9.57 h
INSTRUM Avance Neo 500
PROBHD Z119470_0333 (
PULPROG zg30
TD 65536
SOLVENT CDCl3
NS 16
DS 0
SWH 14705.883 Hz
FIDRES 0.448788 Hz
AQ 2.2282240 sec
RG 95.7854
DW 34.000 usec
DE 6.79 usec
TE 292.3 K
D1 1.00000000 sec
TD0 1
SFO1 500.1730885 MHz
NUC1 1H
P0 3.33 usec
P1 10.00 usec
PLW1 20.93000031 W

F2 - Processing parameters
SI 65536
SF 500.1700071 MHz
WDW EM
SSB 0
LB 0.30 Hz
GB 0
PC 1.00

CH-6
1H_8scan CDCl3 {D:\Spectra} nmr 35

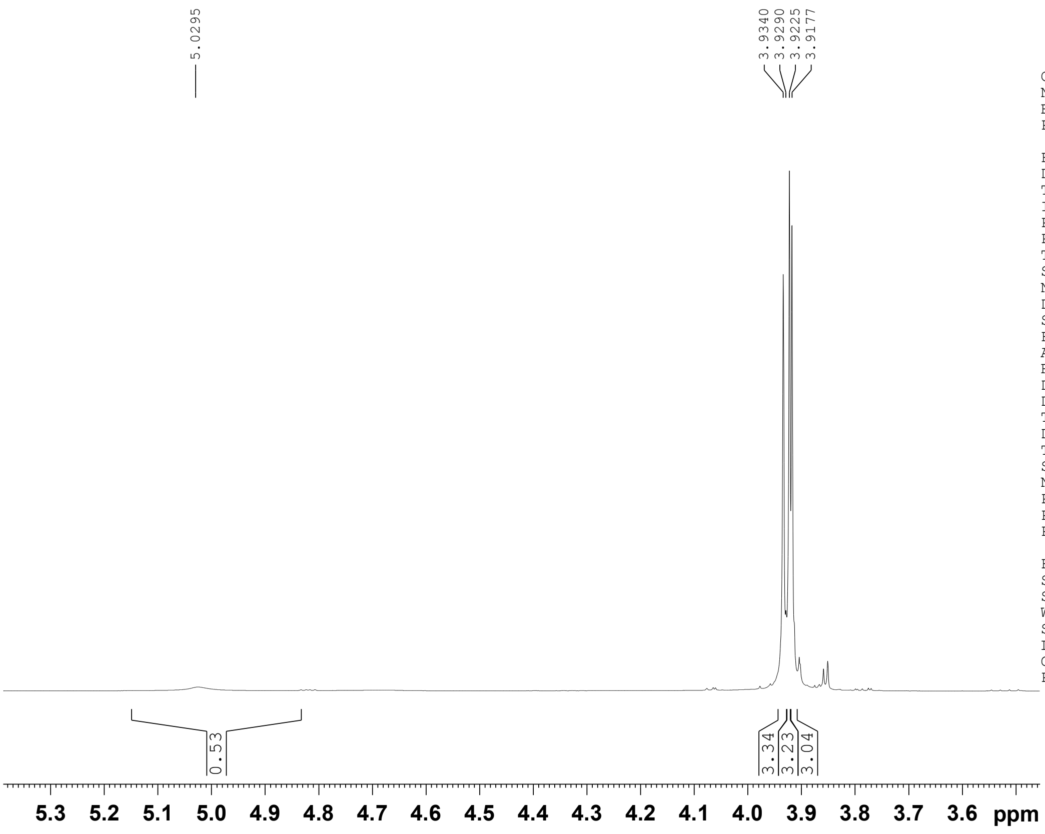

BRUKER
AVANCE NEO
500 MHz NMR
SPECTROMETER
SAIF, P.U.

Current Data Parameters
NAME Apr01-2021
EXPNO 350
PROCNO 1

F2 - Acquisition Parameters
Date_ 20210402
Time 9.57 h
INSTRUM Avance Neo 500
PROBHD Z119470_0333 (
PULPROG zg30
TD 65536
SOLVENT CDCl3
NS 16
DS 0
SWH 14705.883 Hz
FIDRES 0.448788 Hz
AQ 2.2282240 sec
RG 95.7854
DW 34.000 usec
DE 6.79 usec
TE 292.3 K
D1 1.00000000 sec
TD0 1
SFO1 500.1730885 MHz
NUC1 1H
P0 3.33 usec
P1 10.00 usec
PLW1 20.93000031 W

F2 - Processing parameters
SI 65536
SF 500.1700071 MHz
WDW EM
SSB 0
LB 0.30 Hz
GB 0
PC 1.00

CH-6
1H_8scan CDCl3 {D:\Spectra} nmr 35

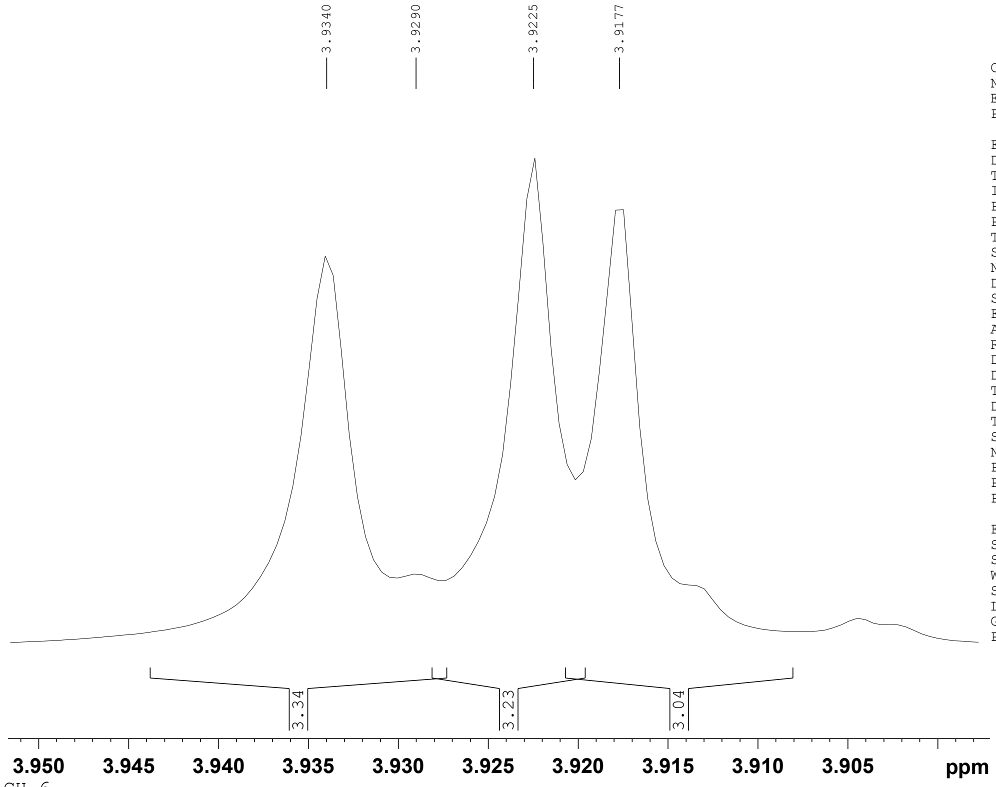

BRUKER
AVANCE NEO
500 MHz NMR
SPECTROMETER
SAIF, P.U.

```
Current Data Parameters
NAME          Apr01-2021
EXPNO                350
PROCNO                 1

F2 - Acquisition Parameters
Date_           20210402
Time                9.57 h
INSTRUM  Avance Neo 500
PROBHD   Z119470_0333 (
PULPROG             zg30
TD                 65536
SOLVENT            CDCl3
NS                    16
DS                     0
SWH            14705.883 Hz
FIDRES          0.448788 Hz
AQ             2.2282240 sec
RG               95.7854
DW                34.000 usec
DE                  6.79 usec
TE                 292.3 K
D1            1.00000000 sec
TD0                    1
SFO1         500.1730885 MHz
NUC1                  1H
P0                  3.33 usec
P1                 10.00 usec
PLW1         20.93000031 W

F2 - Processing parameters
SI                 65536
SF           500.1700071 MHz
WDW                   EM
SSB                    0
LB                  0.30 Hz
GB                     0
PC                  1.00
```

CH-6
C13CPD CDCl3 {D:\Spectra} nmr 35

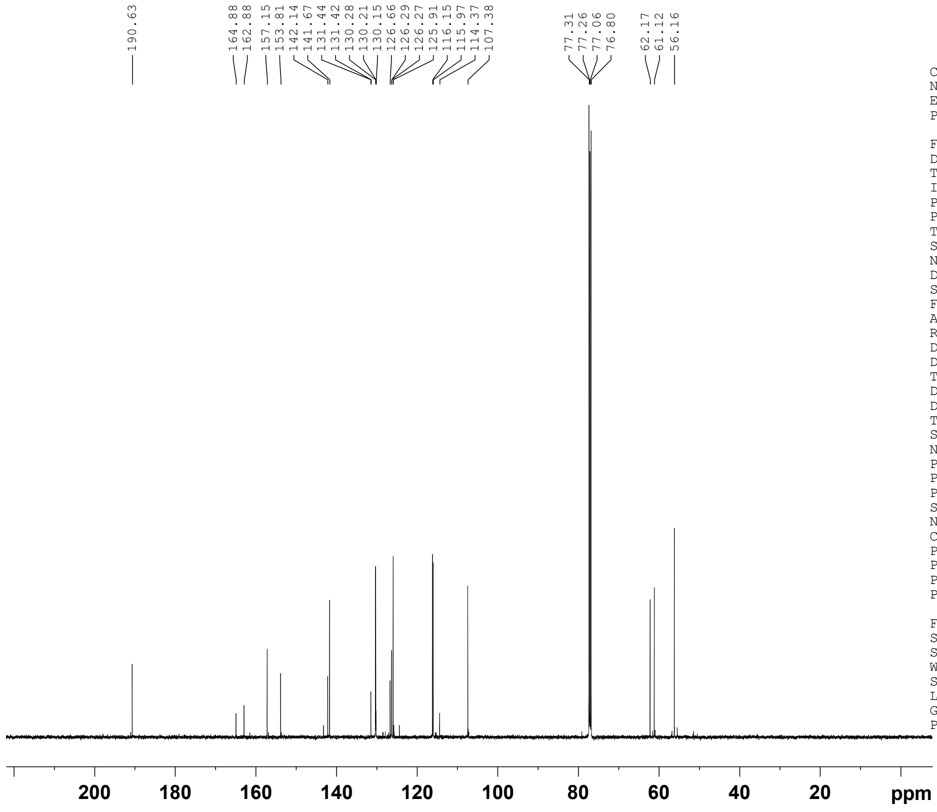

BRUKER
AVANCE NEO
500 MHz NMR SPECTROMETER
SAIF, PANJAB UNIVERSITY,
CHANDIGARH

```
Current Data Parameters
NAME          Apr01-2021
EXPNO                351
PROCNO                 1

F2 - Acquisition Parameters
Date_           20210402
Time               10.48 h
INSTRUM  Avance Neo 500
PROBHD   Z119470_0333 (
PULPROG           zgpg30
TD                 65536
SOLVENT            CDCl3
NS                  1024
DS                     4
SWH            37037.035 Hz
FIDRES          1.130281 Hz
AQ             0.8847360 sec
RG                   101
DW                13.500 usec
DE                  6.50 usec
TE                 293.0 K
D1            2.00000000 sec
D11           0.03000000 sec
TD0                    1
SFO1         125.7804233 MHz
NUC1                 13C
P0                  3.33 usec
P1                 10.00 usec
PLW1         83.14099884 W
SFO2         500.1720007 MHz
NUC2                  1H
CPDPRG[2         waltz65
PCPD2              80.00 usec
PLW2         20.93000031 W
PLW12         0.32703000 W
PLW13         0.16449000 W

F2 - Processing parameters
SI                 32768
SF           125.7678465 MHz
WDW                   EM
SSB                    0
LB                  1.00 Hz
GB                     0
PC                  1.40
```

CH-6
C13CPD CDCl3 {D:\Spectra} nmr 35

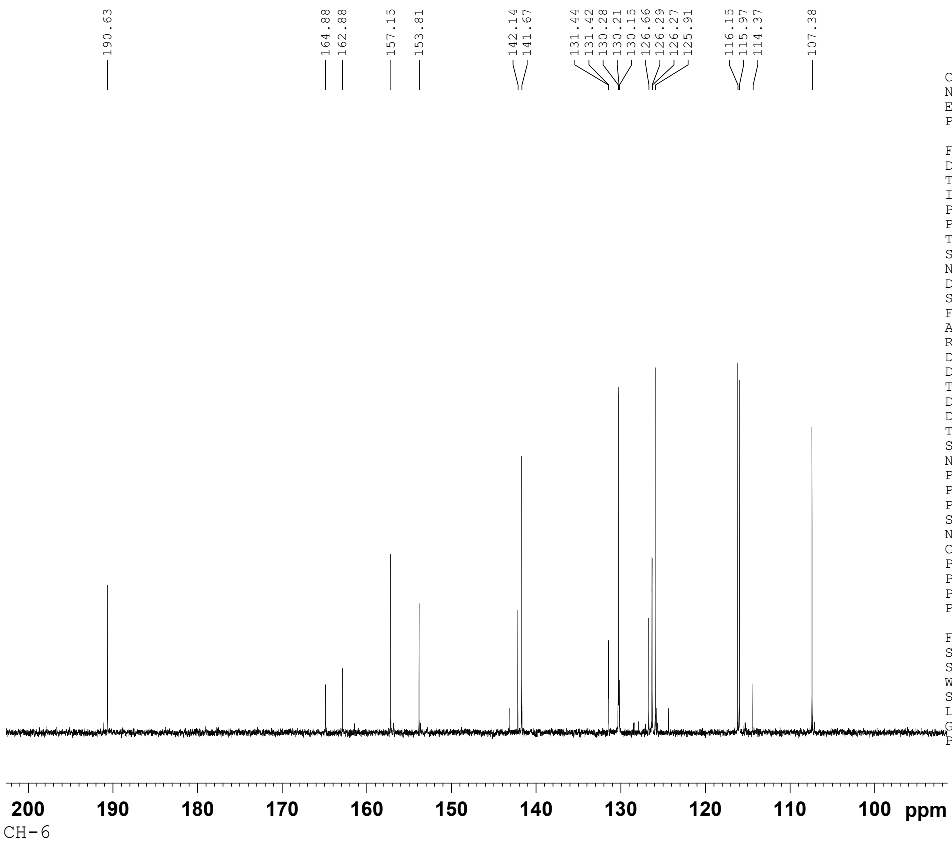

BRUKER
AVANCE NEO
500 MHz NMR SPECTROMETER
SAIF, PANJAB UNIVERSITY,
CHANDIGARH

Current Data Parameters
NAME Apr01-2021
EXPNO 351
PROCNO 1

F2 - Acquisition Parameters
Date_ 20210402
Time 10.48 h
INSTRUM Avance Neo 500
PROBHD Z119470_0333 (
PULPROG zgpg30
TD 65536
SOLVENT CDCl3
NS 1024
DS 4
SWH 37037.035 Hz
FIDRES 1.130281 Hz
AQ 0.8847360 sec
RG 101
DW 13.500 usec
DE 6.50 usec
TE 293.0 K
D1 2.00000000 sec
D11 0.03000000 sec
TD0 1
SFO1 125.7804233 MHz
NUC1 13C
P0 3.33 usec
P1 10.00 usec
PLW1 83.14099884 W
SFO2 500.1720007 MHz
NUC2 1H
CPDPRG[2 waltz65
PCPD2 80.00 usec
PLW2 20.93000031 W
PLW12 0.32703000 W
PLW13 0.16449000 W

F2 - Processing parameters
SI 32768
SF 125.7678465 MHz
WDW EM
SSB 0
LB 1.00 Hz
GB 0
PC 1.40

CH-6
C13CPD CDCl3 {D:\Spectra} nmr 35

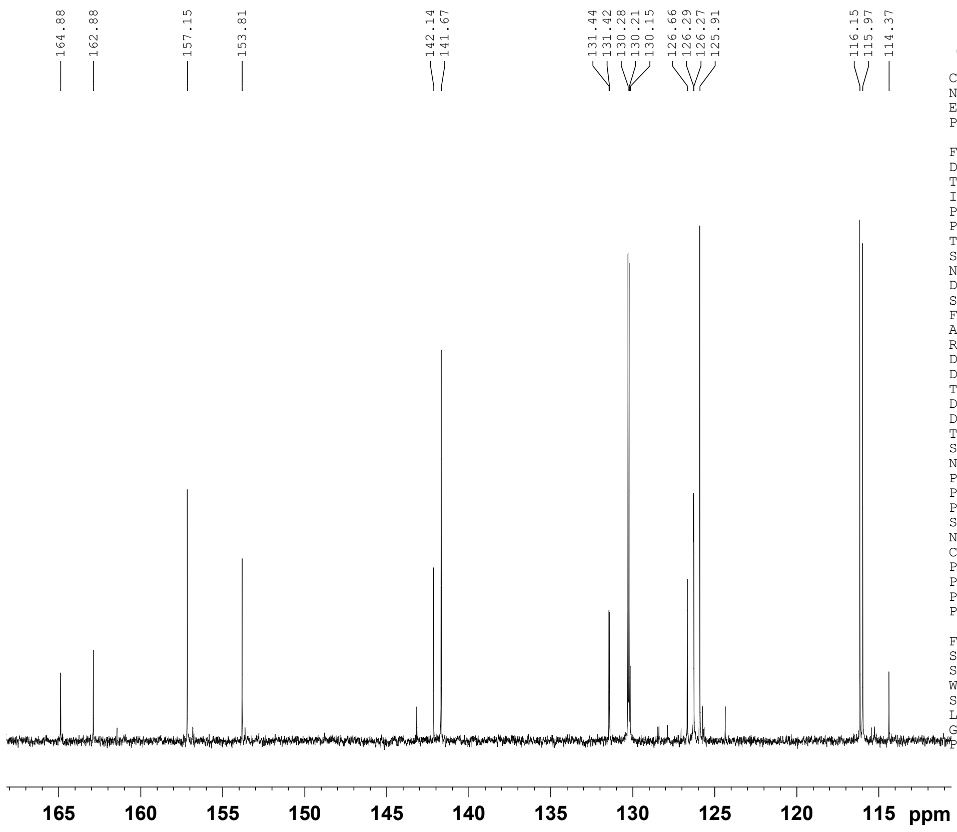

BRUKER
AVANCE NEO
500 MHz NMR SPECTROMETER
SAIF, PANJAB UNIVERSITY,
CHANDIGARH

Current Data Parameters
NAME Apr01-2021
EXPNO 351
PROCNO 1

F2 - Acquisition Parameters
Date_ 20210402
Time 10.48 h
INSTRUM Avance Neo 500
PROBHD Z119470_0333 (
PULPROG zgpg30
TD 65536
SOLVENT CDCl3
NS 1024
DS 4
SWH 37037.035 Hz
FIDRES 1.130281 Hz
AQ 0.8847360 sec
RG 101
DW 13.500 usec
DE 6.50 usec
TE 293.0 K
D1 2.00000000 sec
D11 0.03000000 sec
TD0 1
SFO1 125.7804233 MHz
NUC1 13C
P0 3.33 usec
P1 10.00 usec
PLW1 83.14099884 W
SFO2 500.1720007 MHz
NUC2 1H
CPDPRG[2 waltz65
PCPD2 80.00 usec
PLW2 20.93000031 W
PLW12 0.32703000 W
PLW13 0.16449000 W

F2 - Processing parameters
SI 32768
SF 125.7678465 MHz
WDW EM
SSB 0
LB 1.00 Hz
GB 0
PC 1.40

CH-6
C13CPD CDCl3 {D:\Spectra} nmr 35

BRUKER
AVANCE NEO
500 MHz NMR SPECTROMETER
SAIF, PANJAB UNIVERSITY,
CHANDIGARH

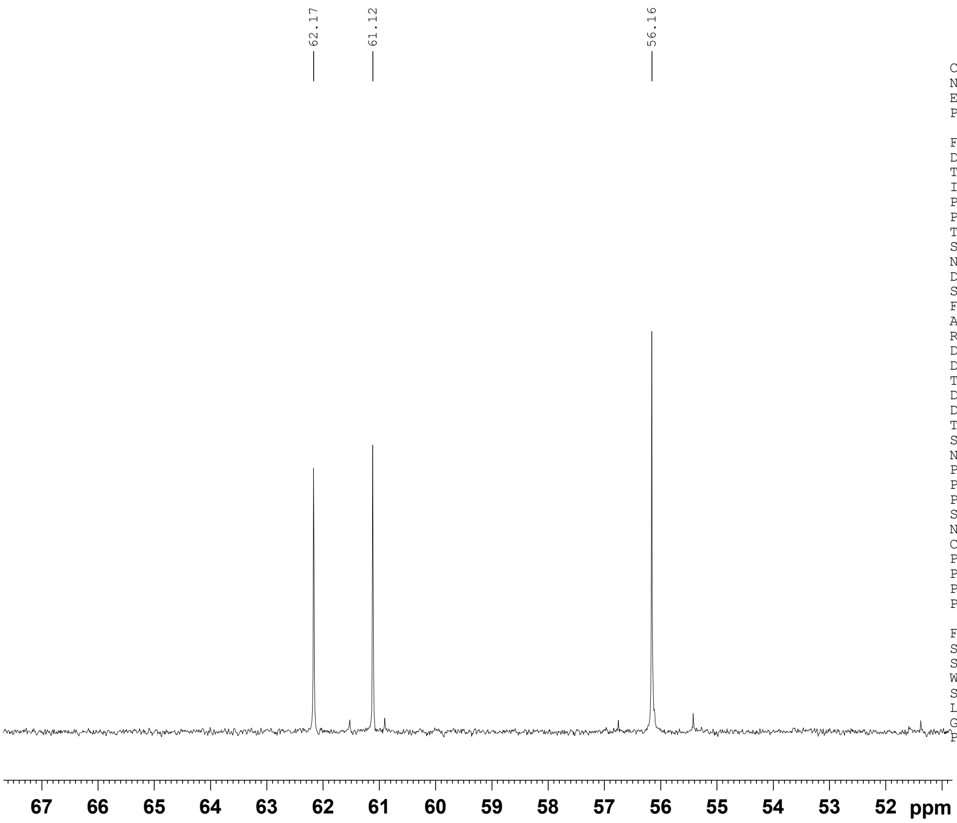

Current Data Parameters	
NAME	Apr01-2021
EXPNO	351
PROCNO	1

F2 - Acquisition Parameters	
Date_	20210402
Time	10.48 h
INSTRUM	Avance Neo 500
PROBHD	Z119470_0333 (
PULPROG	zgpg30
TD	65536
SOLVENT	CDCl3
NS	1024
DS	4
SWH	37037.035 Hz
FIDRES	1.130281 Hz
AQ	0.8847360 sec
RG	101
DW	13.500 usec
DE	6.50 usec
TE	293.0 K
D1	2.00000000 sec
D11	0.03000000 sec
TD0	1
SFO1	125.7804233 MHz
NUC1	13C
P0	3.33 usec
P1	10.00 usec
PLW1	83.14099884 W
SFO2	500.1720007 MHz
NUC2	1H
CPDPRG[2	waltz65
PCPD2	80.00 usec
PLW2	20.93000031 W
PLW12	0.32703000 W
PLW13	0.16449000 W

F2 - Processing parameters	
SI	32768
SF	125.7678465 MHz
WDW	EM
SSB	0
LB	1.00 Hz
GB	0
PC	1.40

Blank02SAIF,PANJAB UNIVERSITY ,CHANDIGARH SYNAPT-XS#DBA064 24-May-2021 13:11:55

VISHAL CH-1 5 (0.120) Cm (5:6) 1: TOF MS ES+ 1.04e7

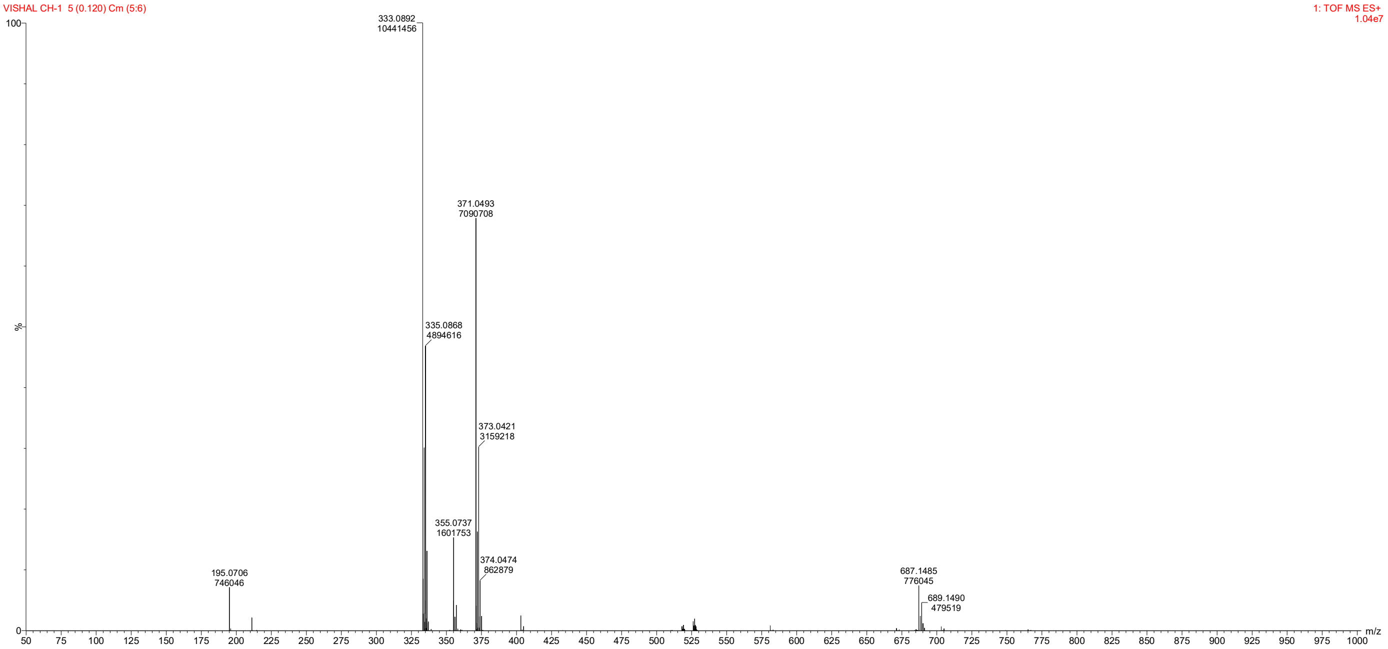

Blank02SAIF,PANJAB UNIVERSITY ,CHANDIGARH SYNAPT-XS#DBA064 24-May-2021 13:11:55

VISHAL CH-1 5 (0.120) Cm (5:6) 1: TOF MS ES+ 1.04e7

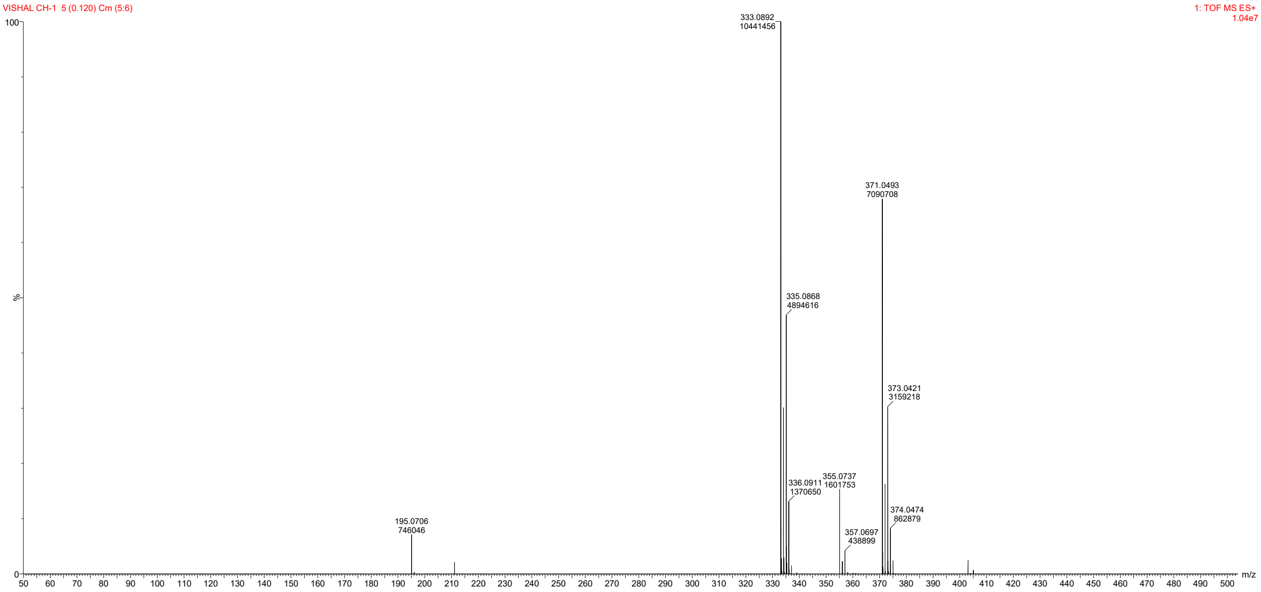

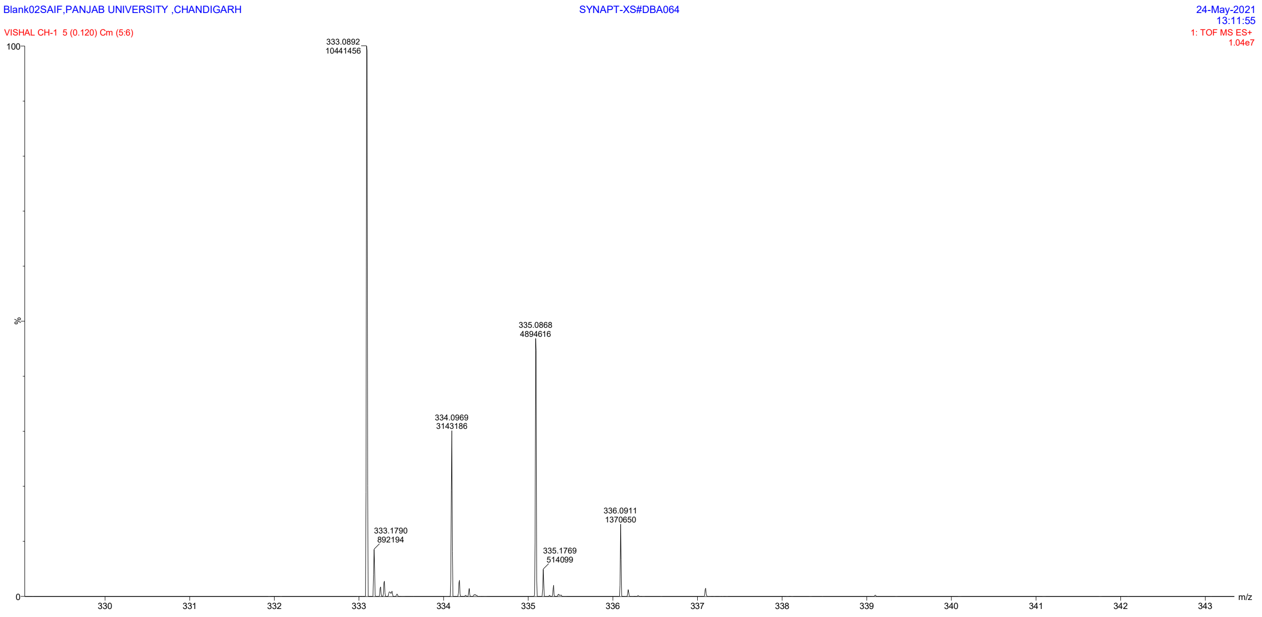
Blank02SAIF,PANJAB UNIVERSITY ,CHANDIGARH
SYNAPT-XS#DBA064
24-May-2021
13:11:55
VISHAL CH-1 5 (0.120) Cm (5:6)
1: TOF MS ES+
1.04e7
333.0892
10441456
335.0868
4894616
334.0969
3143186
333.1790
892194
336.0911
1370650
335.1769
514099
%
m/z
330
331
332
333
334
335
336
337
338
339
340
341
342
343

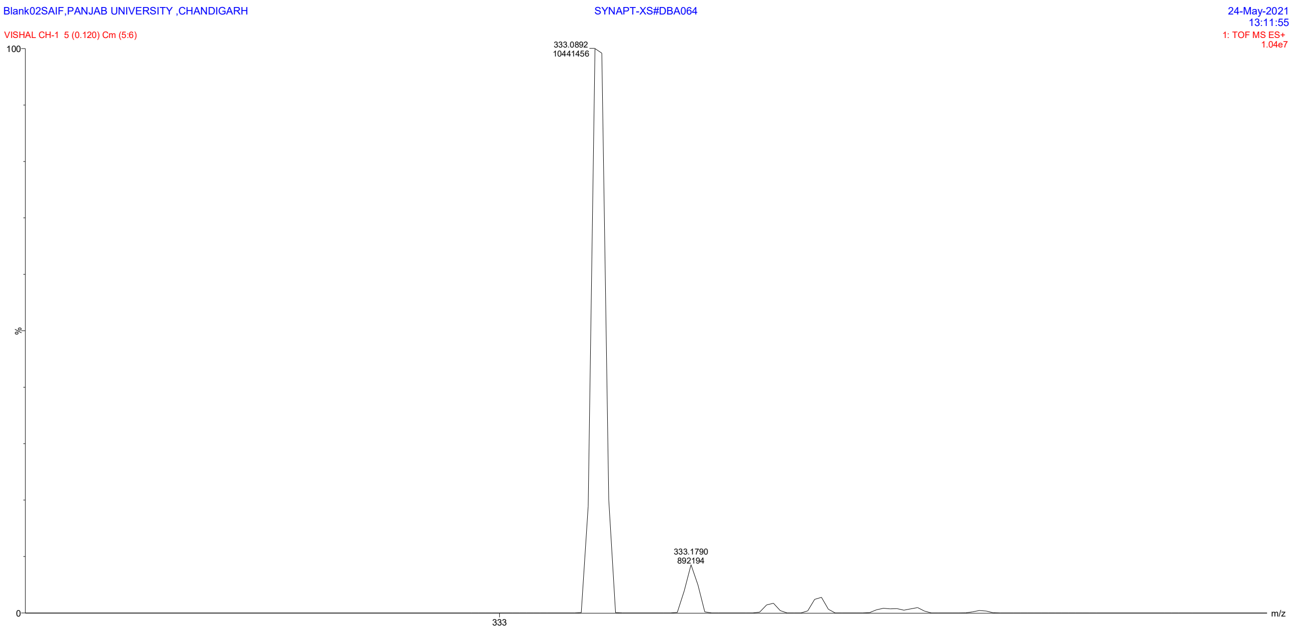
Blank02SAIF,PANJAB UNIVERSITY ,CHANDIGARH
SYNAPT-XS#DBA064
24-May-2021
13:11:55
VISHAL CH-1 5 (0.120) Cm (5:6)
1: TOF MS ES+
1.04e7
333.0892
10441456
333.1790
892194
%
m/z
333

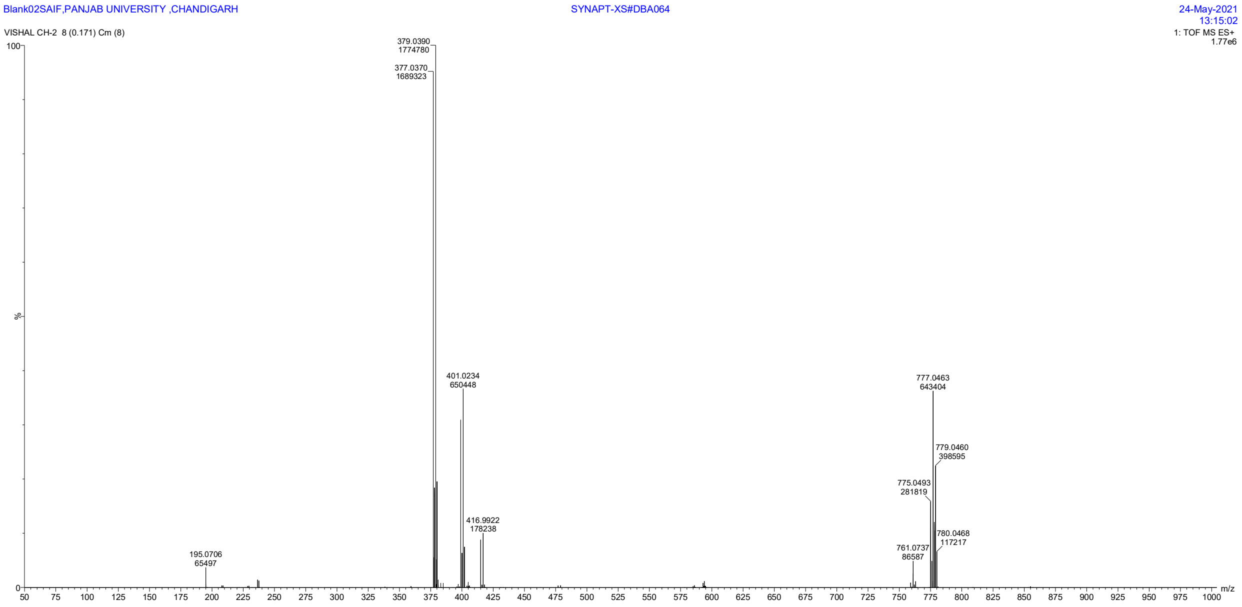
Blank02SAIF,PANJAB UNIVERSITY ,CHANDIGARH
SYNAPT-XS#DBA064
24-May-2021
13:15:02
VISHAL CH-2 8 (0.171) Cm (8)
1: TOF MS ES+
1.77e6
379.0390
1774780
377.0370
1689323
401.0234
650448
416.9922
178238
195.0706
65497
777.0463
643404
779.0460
398595
775.0493
281819
780.0468
117217
761.0737
86587
%
m/z

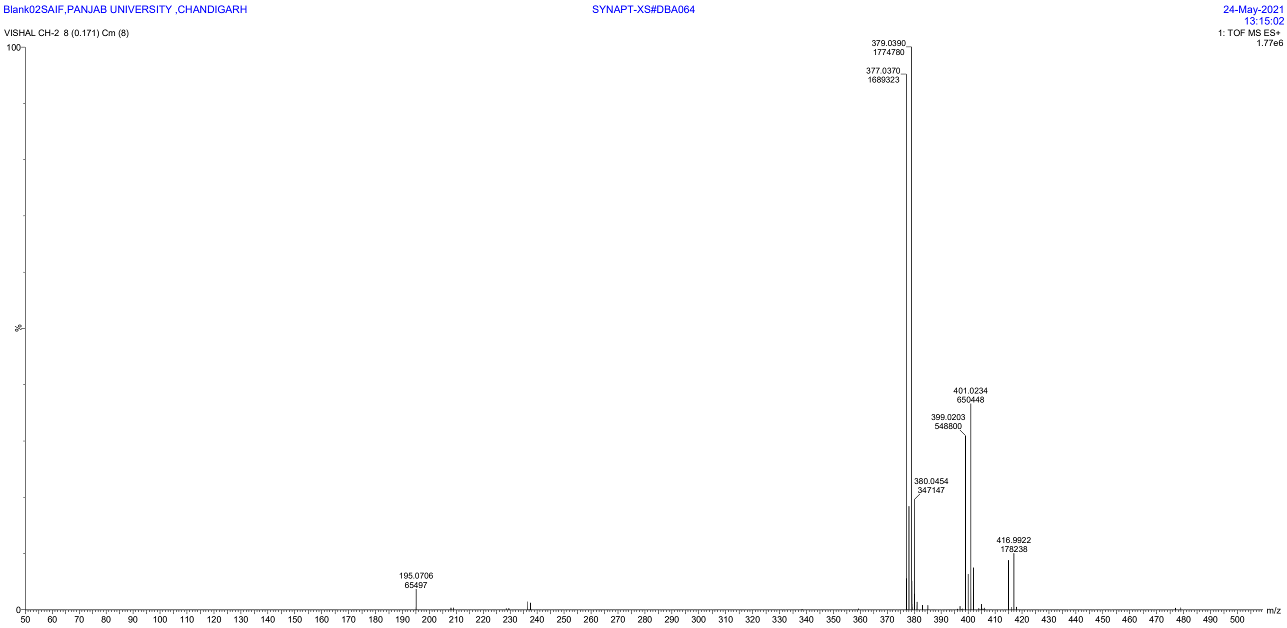
Blank02SAIF,PANJAB UNIVERSITY ,CHANDIGARH
SYNAPT-XS#DBA064
24-May-2021
13:15:02
VISHAL CH-2 8 (0.171) Cm (8)
1: TOF MS ES+
1.77e6
379.0390
1774780
377.0370
1689323
401.0234
650448
399.0203
548800
380.0454
347147
416.9922
178238
195.0706
65497
%
m/z

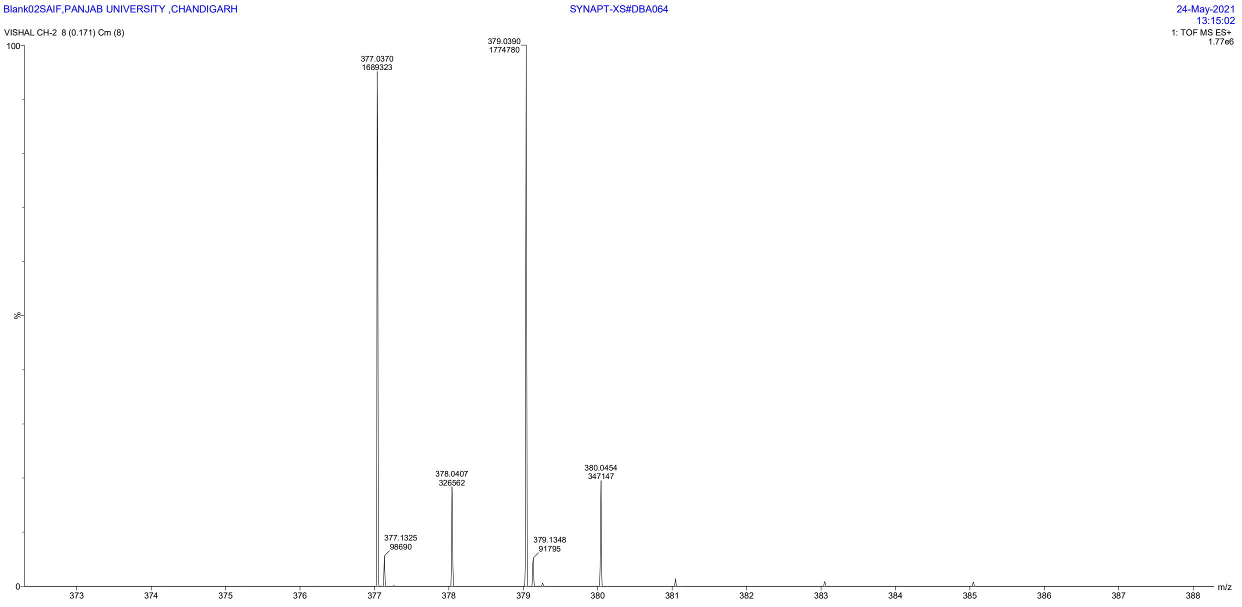

Blank02SAIF,PANJAB UNIVERSITY ,CHANDIGARH
SYNAPT-XS#DBA064
24-May-2021
13:15:02
VISHAL CH-2 8 (0.171) Cm (8)
1: TOF MS ES+
1.77e6
377.0370
1689323
379.0390
1774780
378.0407
326562
380.0454
347147
377.1325
98690
379.1348
91795
m/z

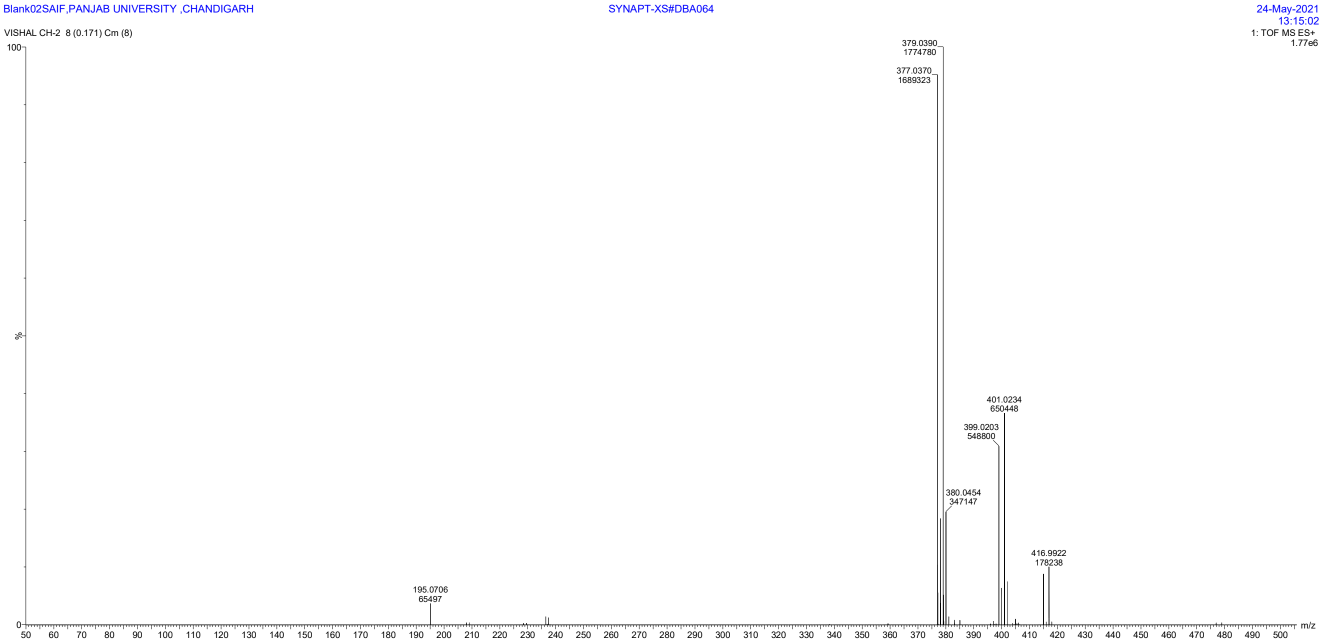

Blank02SAIF,PANJAB UNIVERSITY ,CHANDIGARH
SYNAPT-XS#DBA064
24-May-2021
13:15:02
VISHAL CH-2 8 (0.171) Cm (8)
1: TOF MS ES+
1.77e6
379.0390
1774780
377.0370
1689323
401.0234
650448
399.0203
548800
380.0454
347147
416.9922
178238
195.0706
65497
m/z

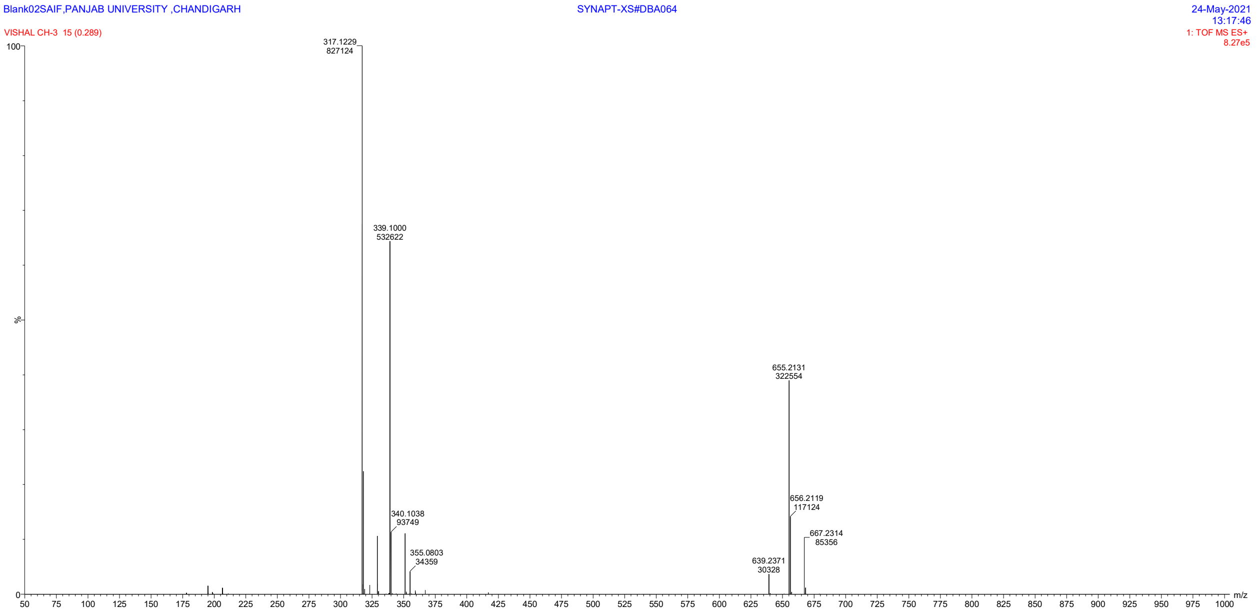
Blank02SAIF,PANJAB UNIVERSITY ,CHANDIGARH
SYNAPT-XS#DBA064
24-May-2021
13:17:46
VISHAL CH-3 15 (0.289)
1: TOF MS ES+
8.27e5
317.1229
827124
339.1000
532622
340.1038
93749
355.0803
34359
655.2131
322554
656.2119
117124
667.2314
85356
639.2371
30328
%
m/z

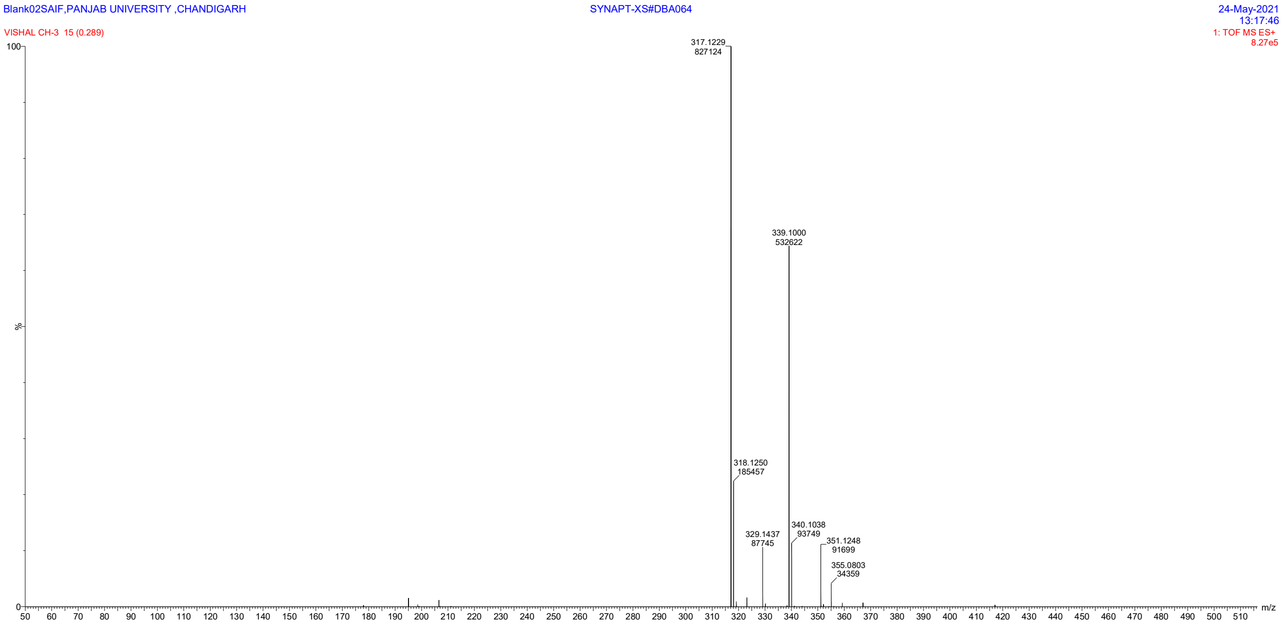
Blank02SAIF,PANJAB UNIVERSITY ,CHANDIGARH
SYNAPT-XS#DBA064
24-May-2021
13:17:46
VISHAL CH-3 15 (0.289)
1: TOF MS ES+
8.27e5
317.1229
827124
339.1000
532622
318.1250
185457
329.1437
87745
340.1038
93749
351.1248
91699
355.0803
34359
%
m/z

Blank02SAIF,PANJAB UNIVERSITY ,CHANDIGARH SYNAPT-XS#DBA064 24-May-2021 13:17:46

VISHAL CH-3 15 (0.289) 1: TOF MS ES+ 8.27e5

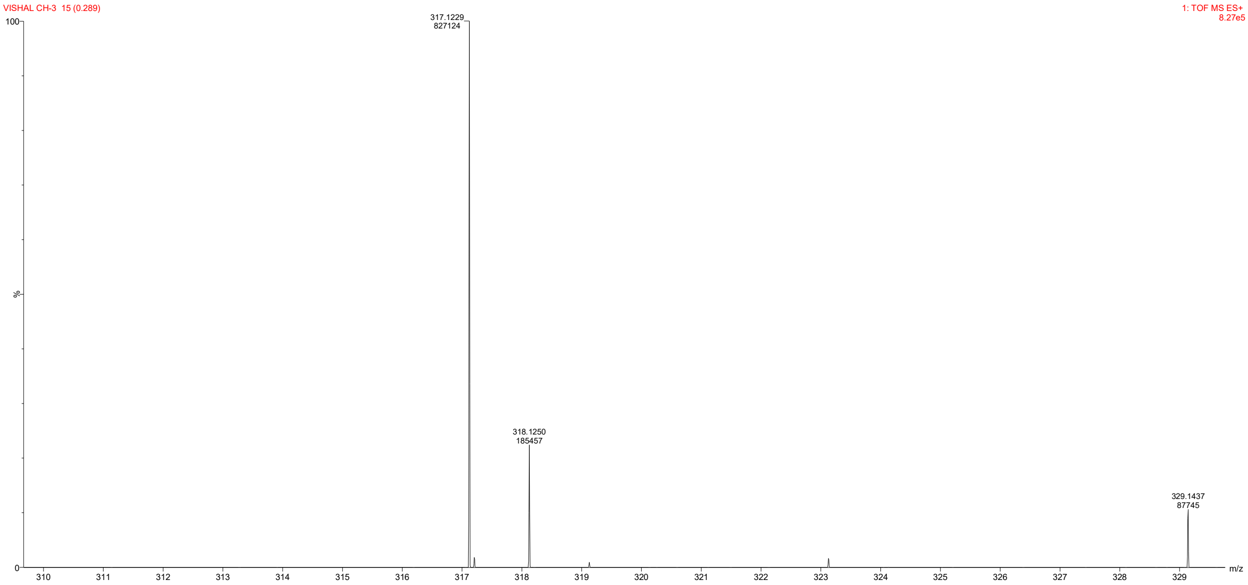

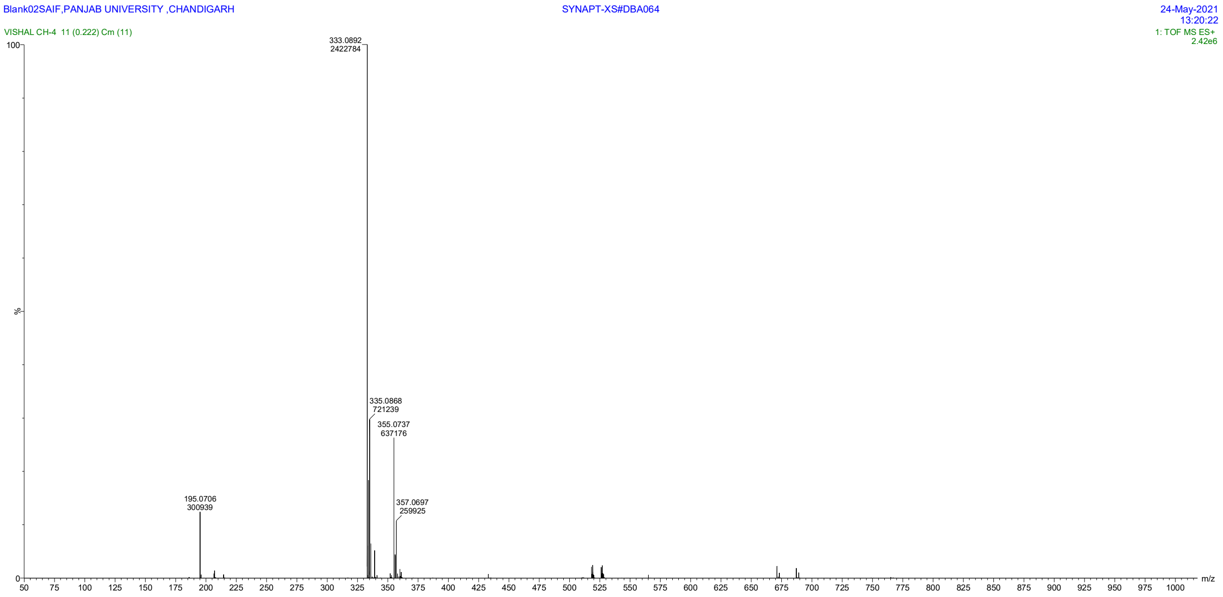

Blank02SAIF,PANJAB UNIVERSITY ,CHANDIGARH
SYNAPT-XS#DBA064
24-May-2021
13:20:22
VISHAL CH-4 11 (0.222) Cm (11)
1: TOF MS ES+
2.42e6
333.0892
2422784
335.0868
721239
355.0737
637176
195.0706
300939
357.0697
259925
%
m/z

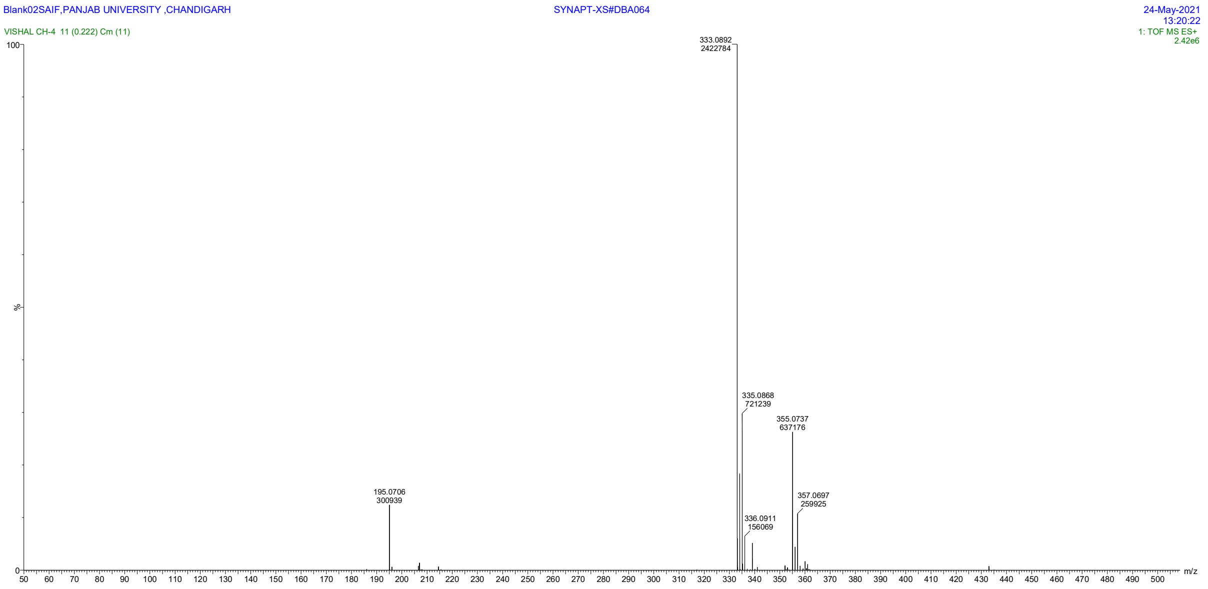

Blank02SAIF,PANJAB UNIVERSITY ,CHANDIGARH
SYNAPT-XS#DBA064
24-May-2021
13:20:22
VISHAL CH-4 11 (0.222) Cm (11)
1: TOF MS ES+
2.42e6
333.0892
2422784
335.0868
721239
355.0737
637176
195.0706
300939
336.0911
156069
357.0697
259925
%
m/z

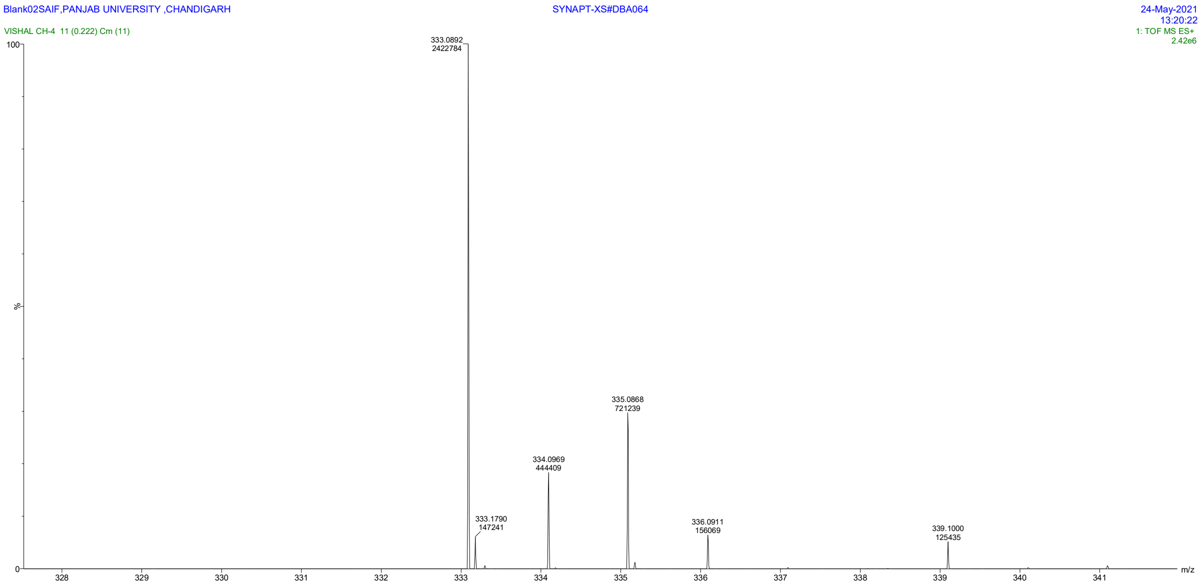

Blank02SAIF,PANJAB UNIVERSITY ,CHANDIGARH
SYNAPT-XS#DBA064
24-May-2021
13:20:22
VISHAL CH-4 11 (0.222) Cm (11)
1: TOF MS ES+
2.42e6
333.0892
2422784
333.1790
147241
334.0969
444409
335.0868
721239
336.0911
156069
339.1000
125435
%
m/z

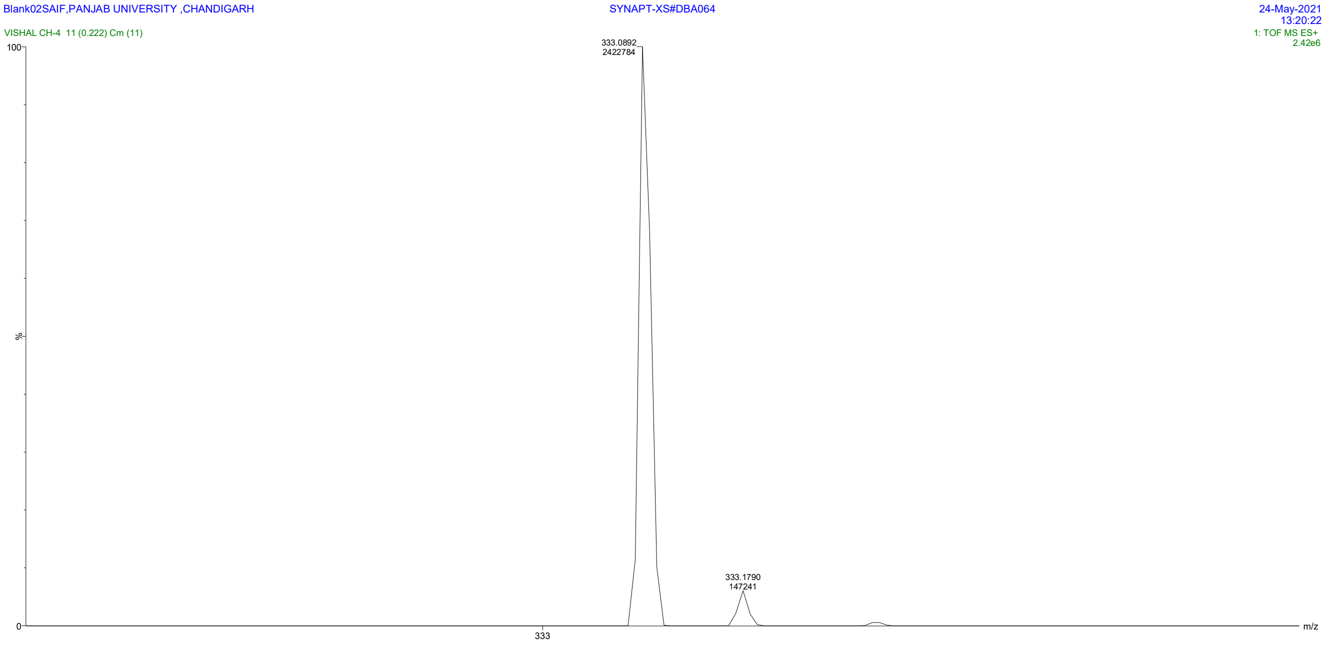

Blank02SAIF,PANJAB UNIVERSITY ,CHANDIGARH
SYNAPT-XS#DBA064
24-May-2021
13:20:22
VISHAL CH-4 11 (0.222) Cm (11)
1: TOF MS ES+
2.42e6
333.0892
2422784
333.1790
147241
%
333
m/z

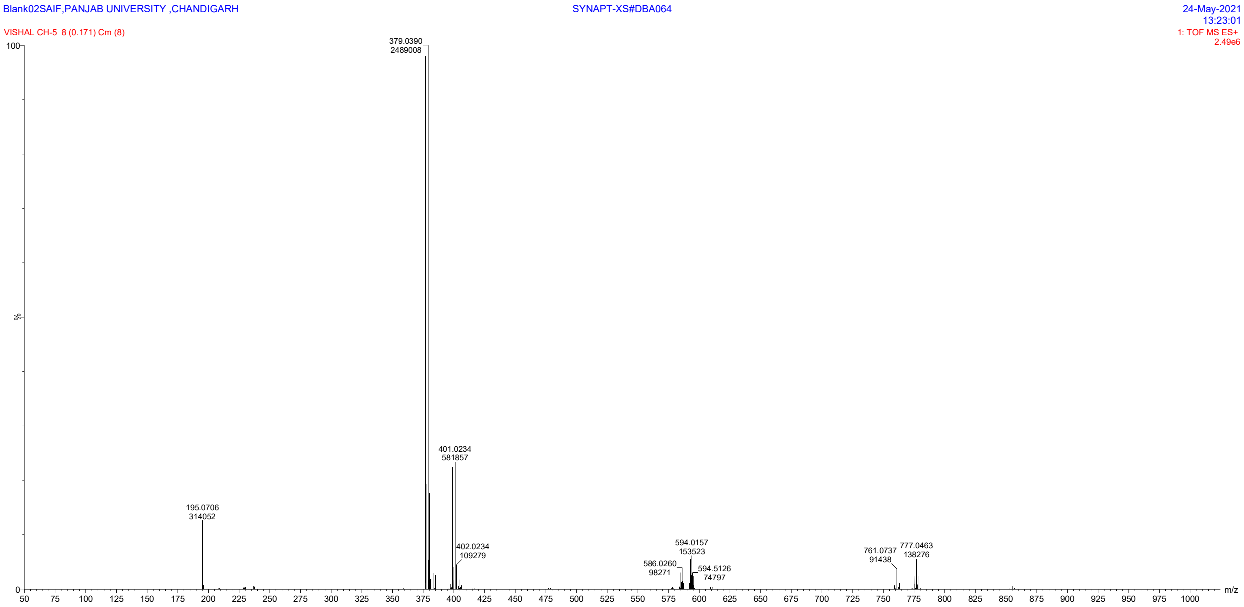
Blank02SAIF,PANJAB UNIVERSITY ,CHANDIGARH
SYNAPT-XS#DBA064
24-May-2021
13:23:01
VISHAL CH-5 8 (0.171) Cm (8)
1: TOF MS ES+
2.49e6
379.0390
2489008
401.0234
581857
195.0706
314052
402.0234
109279
594.0157
153523
586.0260
98271
594.5126
74797
761.0737
91438
777.0463
138276
m/z

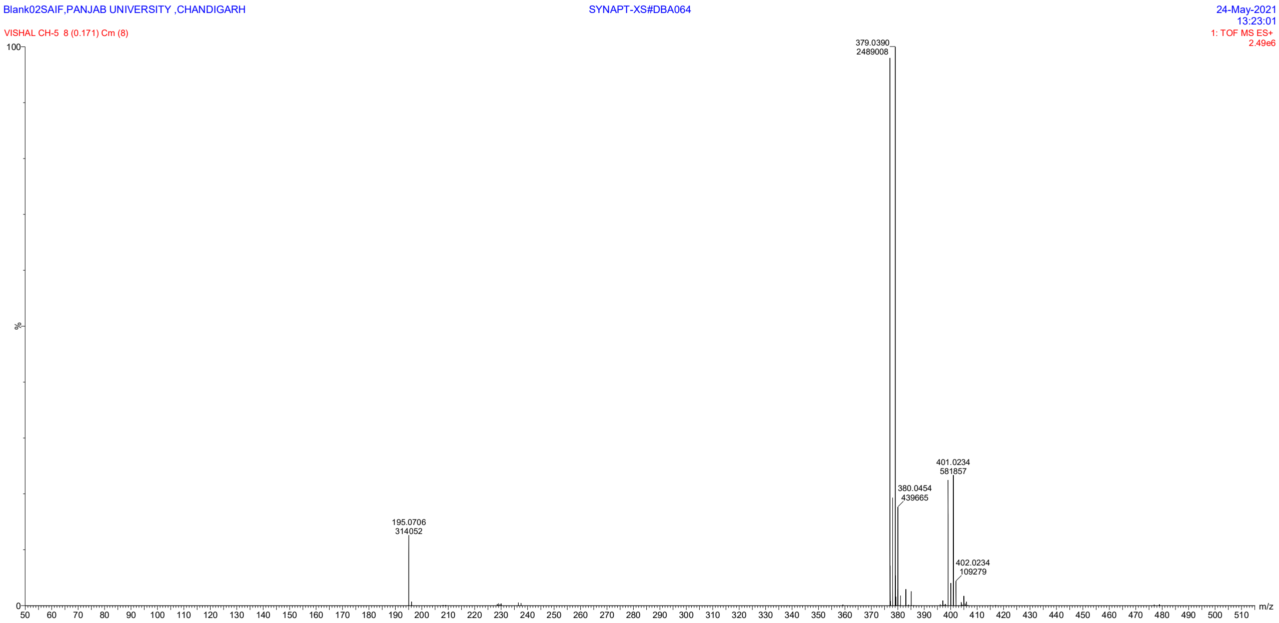
Blank02SAIF,PANJAB UNIVERSITY ,CHANDIGARH
SYNAPT-XS#DBA064
24-May-2021
13:23:01
VISHAL CH-5 8 (0.171) Cm (8)
1: TOF MS ES+
2.49e6
379.0390
2489008
401.0234
581857
380.0454
439665
195.0706
314052
402.0234
109279
m/z

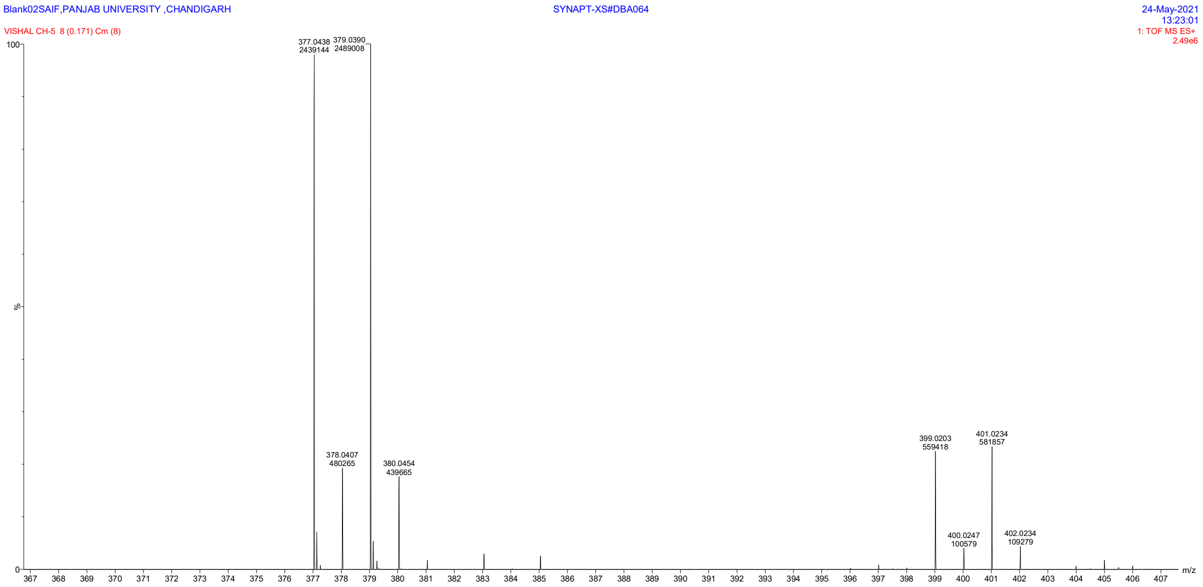
Blank02SAIF,PANJAB UNIVERSITY ,CHANDIGARH
SYNAPT-XS#DBA064
24-May-2021
13:23:01
VISHAL CH-5 8 (0.171) Cm (8)
1: TOF MS ES+
2.49e6
377.0438
2439144
379.0390
2489008
378.0407
480265
380.0454
439665
399.0203
559418
401.0234
581857
400.0247
100579
402.0234
109279
%
m/z

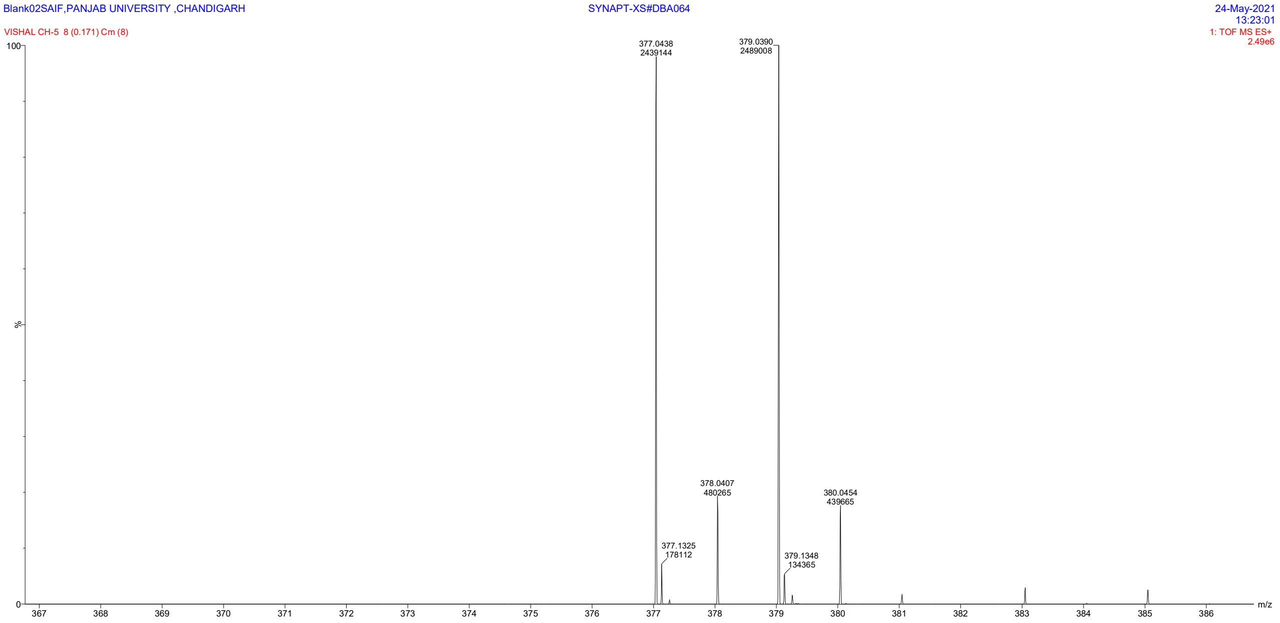
Blank02SAIF,PANJAB UNIVERSITY ,CHANDIGARH
SYNAPT-XS#DBA064
24-May-2021
13:23:01
VISHAL CH-5 8 (0.171) Cm (8)
1: TOF MS ES+
2.49e6
377.0438
2439144
379.0390
2489008
378.0407
480265
380.0454
439665
377.1325
178112
379.1348
134365
%
m/z

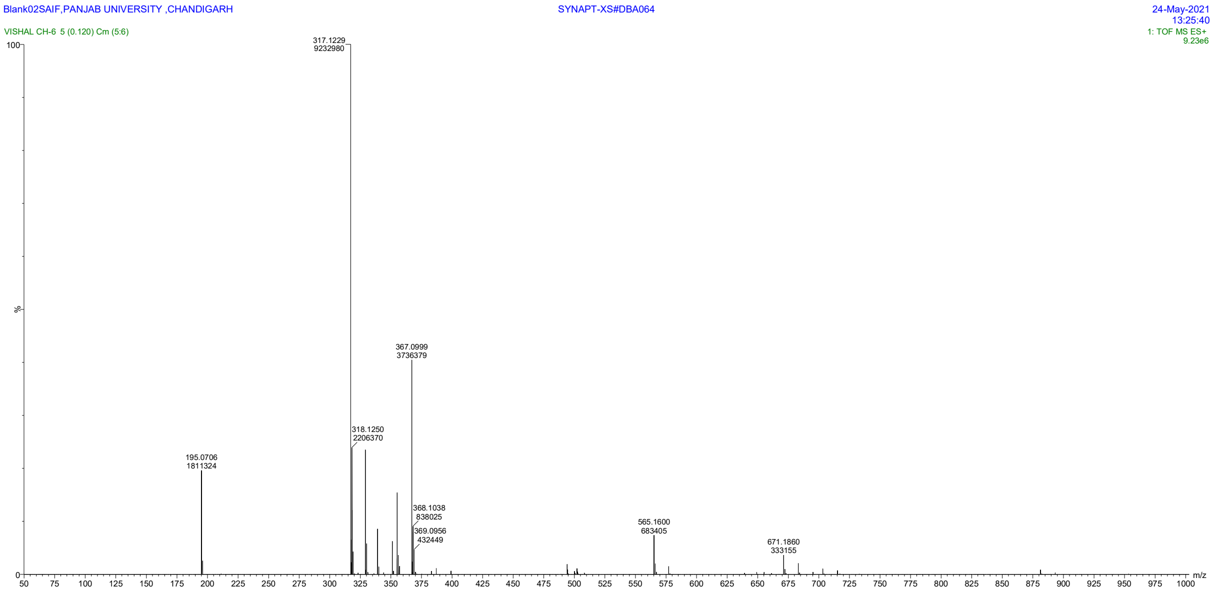
Blank02SAIF,PANJAB UNIVERSITY ,CHANDIGARH
SYNAPT-XS#DBA064
24-May-2021
13:25:40
VISHAL CH-6 5 (0.120) Cm (5:6)
1: TOF MS ES+
9.23e6
317.1229
9232980
367.0999
3736379
318.1250
2206370
195.0706
1811324
368.1038
838025
369.0956
432449
565.1600
683405
671.1860
333155
%
m/z

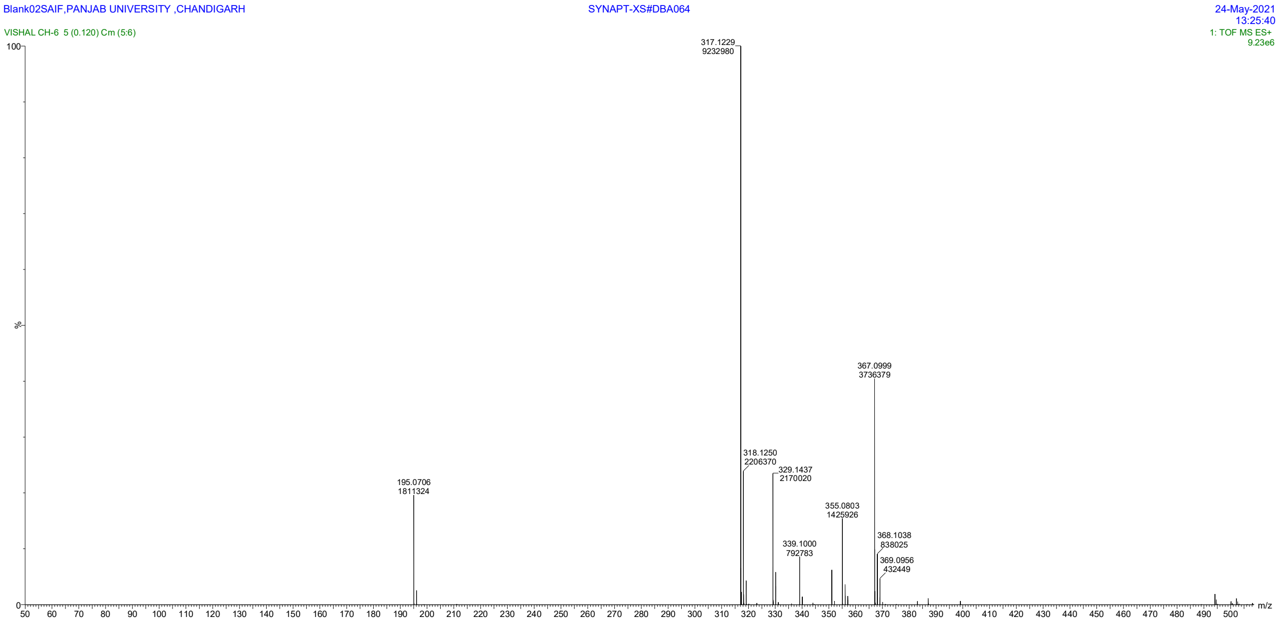
Blank02SAIF,PANJAB UNIVERSITY ,CHANDIGARH
SYNAPT-XS#DBA064
24-May-2021
13:25:40
VISHAL CH-6 5 (0.120) Cm (5:6)
1: TOF MS ES+
9.23e6
317.1229
9232980
367.0999
3736379
318.1250
2206370
329.1437
2170020
195.0706
1811324
355.0803
1425926
339.1000
792783
368.1038
838025
369.0956
432449
%
m/z

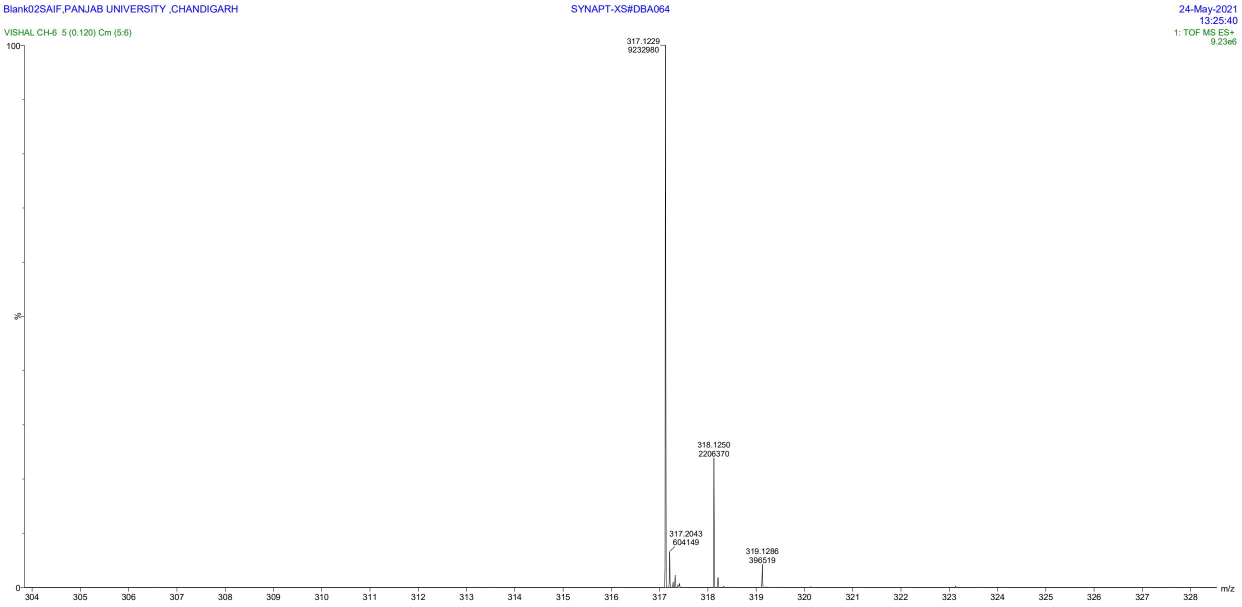

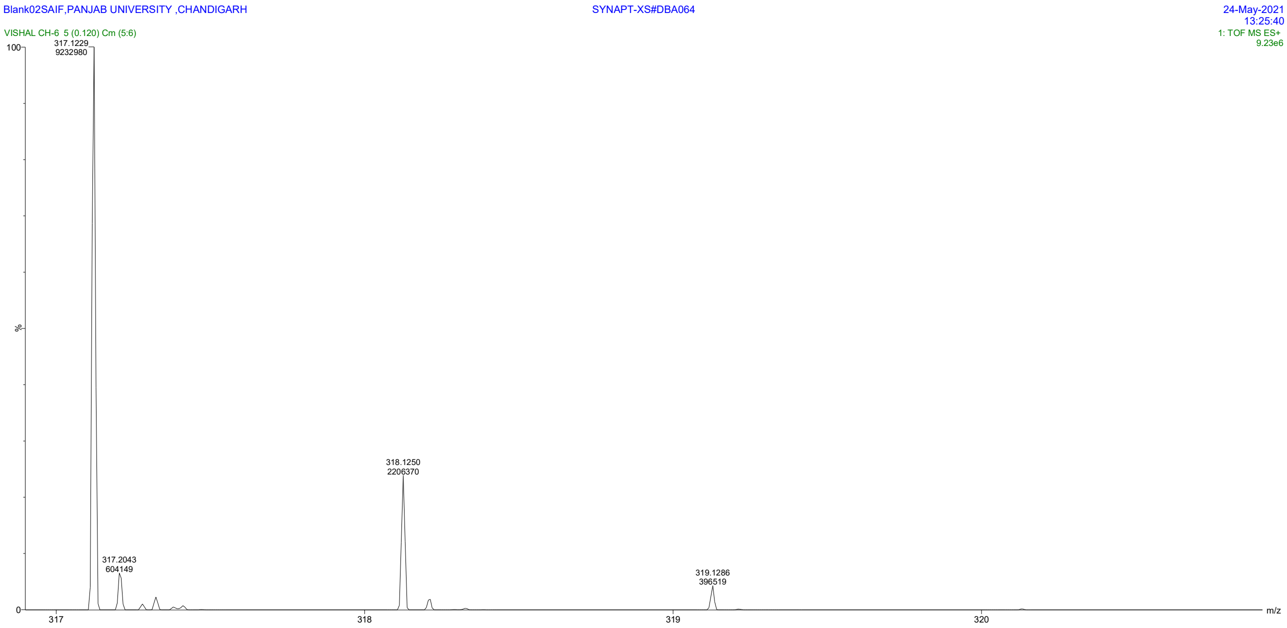

1.2. Cytotoxicity Studies

The cell line of Vero (African green monkey kidney cells) was procured from NCCS, Pune and were grown in liquid medium (DMEM) containing, 100 ug/mL penicillin, 100 µg/mL streptomycin, and 10% Fetal Bovine Serum (FBS) and preserved under an atmosphere of 5% CO_2 at 37°C. The **CH4** and **CH5** sample was assayed for *in vitro* cytotoxicity by MTT assay using the cultured Vero cells. Briefly, the cultured Vero cells were produced by cell dissociation with trypsin (trypsinization), collectively in a 15 mL tube. At a density of $1{\times}10^5$ cells/mL cells/well (200 µL) the cells were plated for 24-48 hour at 37°C into 96-well tissue culture plate in DMEM medium containing 10 % FBS and 1% antibiotic solution. The wells were washed with sterile Phosphate buffered saline and further allowed to react with varying concentrations of the **CH4** and **CH5** sample in a serum free DMEM medium. The samples were triplicated and the cells were kept for incubation at 37°C for 24 h in a humidified 5% CO_2 incubator. MTT (20 µL of 5 mg/mL) was added into each well after the incubation and the cells were incubated for another 2–4

h. The end point was determined by the purple precipitation which was clearly seen under an inverted microscope. At the end, the medium along with MTT (220 μL) were used for the aspiration of the wells and later washed with 1X PBS (200 μL). In order, to dissolve formazan crystals, DMSO (100 μL) was added to the plate with shaking for 5 min. The absorbance at 570 nm was measured using a micro plate reader (Thermo Fisher Scientific, USA). The IC_{50} value and the percentage cell viability was calculated using GraphPad Prism 8.0 software (USA).

1.3. ROS Assay

The **CH5 and CH4** sample was tested for ROS using Vero cells. In Brief, the cultured Vero cells were grown by cell dissociation with trypsin (trypsinization), in a 15 mL tube. Then, the cells were plated at a density of 1×10^6 cells/mL into 24-well tissue culture plate in DMEM medium containing 10 % FBS and 1% antibiotic solution for 24 hour at 37°C. The wells were washed and pretreated with 126.4 μg/mL of **CH4** and **CH5** sample in serum free DMEM medium and incubated at 37°C for 24 h. 24 h later, 1 mL of ROS assay buffer was added followed by 100 μl of 1X ROS assay staining solution was added to the wells and mixed gently. Then the plate was incubated for 60 minutes in a 37°C incubator with 5% CO_2. After the incubation period, the cells were treated with 100 μM/mL of 30% H_2O_2 and the production of ROS was evaluated immediately by fluorescence imaging system (ZOE, BIO-RAD).

1.4. Mussel Prediction

ligando

Prediction for ligando: COc2ccc(C(=O)/C=C/c1ccc(Cl)cc1)c(OC)c2OC

Position	Target	Score	Reliabil
1	ATP-binding cassette sub-family G member 2:Homo sapiens	86.490 % (11.244)	YES
2	Induced myeloid leukemia cell differentiation protein Mcl-1:Homo sapiens	85.164 % (11.071)	YES
3	Tubulin beta-1 chain:Homo sapiens	84.560 % (10.993)	YES
4	Trophozoite cysteine proteinase:Plasmodium falciparum	84.530 % (10.989)	YES
5	Protein kinase Pfmrk:Plasmodium falciparum	82.602 % (10.738)	YES
6	Tumor necrosis factor receptor R1:Homo sapiens	81.475 % (10.592)	YES
7	Cytochrome P450 1A2:Homo sapiens	80.777 % (10.501)	YES
8	Phosphodiesterase 5A:Bos taurus	77.728 % (10.105)	YES
9	Monoamine oxidase B:Homo sapiens	76.364 % (9.927)	YES
10	Arachidonate 5-lipoxygenase:Homo sapiens	76.000 % (9.880)	YES
11	Aldose reductase:Rattus norvegicus	74.934 % (9.741)	YES
12	Arachidonate 5-lipoxygenase:Rattus norvegicus	74.404 % (9.673)	YES
13	P-glycoprotein 1:Homo sapiens	73.479 % (9.552)	YES
14	Cytochrome P450 2C9:Homo sapiens	71.337 % (9.274)	YES
15	Nitric oxide synthase, inducible:Mus musculus	71.118 % (9.245)	YES
16	Cytochrome P450 2D6:Homo sapiens	70.560 % (9.173)	YES
17	Beta amyloid A4 protein:Homo sapiens	69.747 % (9.067)	YES
18	Cytochrome P450 1A1:Homo sapiens	65.914 % (8.569)	YES
19	Cytochrome P450 1B1:Homo sapiens	65.914 % (8.569)	YES
20	Cytochrome P450 3A4:Homo sapiens	64.527 % (8.389)	YES
21	Probable low molecular weight protein-tyrosine-phosphatase:Mycobacterium tuberculosis	64.073 % (8.329)	YES
22	Cytochrome P450 2C19:Homo sapiens	59.455 % (7.729)	YES
23	Acetylcholinesterase:Electrophorus electricus	58.430 % (7.596)	YES
24	Tubulin alpha chain:Sus scrofa	57.078 % (7.420)	YES
25	Cyclooxygenase-2:Ovis aries	54.038 % (7.025)	YES

ligando

Prediction for ligando: COc2ccc(C(=O)/C=C/c1ccc(Br)cc1)c(OC)c2OC

Position	Target	Score	Reliability	Similars
1	Tumor necrosis factor receptor R1:Homo sapiens	85.930 % (11.171)	YES	6
2	Tubulin beta-1 chain:Homo sapiens	85.244 % (11.082)	YES	4
3	Protein kinase Pfmrk:Plasmodium falciparum	82.748 % (10.757)	YES	5
4	Arachidonate 5-lipoxygenase:Homo sapiens	81.072 % (10.539)	YES	5
5	ATP-binding cassette sub-family G member 2:Homo sapiens	80.597 % (10.478)	YES	6
6	Phosphodiesterase 5A:Bos taurus	77.857 % (10.121)	YES	3
7	Induced myeloid leukemia cell differentiation protein Mcl-1:Homo sapiens	74.622 % (9.701)	YES	3
8	Cytochrome P450 1A2:Homo sapiens	74.270 % (9.655)	YES	5
9	Arachidonate 5-lipoxygenase:Rattus norvegicus	74.073 % (9.629)	YES	7
10	Nitric oxide synthase, inducible:Mus musculus	71.100 % (9.243)	YES	6
11	Beta amyloid A4 protein:Homo sapiens	70.625 % (9.181)	YES	4
12	Cytochrome P450 2D6:Homo sapiens	70.621 % (9.181)	YES	4
13	Aldose reductase:Rattus norvegicus	69.381 % (9.020)	YES	5
14	P-glycoprotein 1:Homo sapiens	68.308 % (8.880)	YES	6
15	Trophozoite cysteine proteinase:Plasmodium falciparum	66.135 % (8.598)	YES	5
16	Cytochrome P450 1A1:Homo sapiens	65.663 % (8.536)	YES	7
17	Cytochrome P450 1B1:Homo sapiens	65.663 % (8.536)	YES	7
18	Cytochrome P450 2C9:Homo sapiens	64.978 % (8.447)	YES	3
19	Cytochrome P450 3A4:Homo sapiens	64.586 % (8.396)	YES	5
20	Probable low molecular weight protein-tyrosine-phosphatase:Mycobacterium tuberculosis	64.041 % (8.325)	YES	5
21	Protein-tyrosine phosphatase 1B:Homo sapiens	61.521 % (7.998)	YES	5
22	Cathepsin L:Homo sapiens	60.967 % (7.926)	YES	3
23	Cytochrome P450 2C19:Homo sapiens	59.689 % (7.760)	YES	1
24	Integrase:Human immunodeficiency virus 1	59.565 % (7.743)	YES	6
25	Cyclooxygenase-2:Mus musculus	58.451 % (7.599)	YES	3

Position	Target	Score	Reliability	Similars
26	Tubulin alpha chain:Sus scrofa	57.365 % (7.457)	YES	8
27	Acetylcholinesterase:Electrophorus electricus	57.244 % (7.442)	YES	9
28	Monoamine oxidase B:Homo sapiens	57.032 % (7.414)	YES	11
29	Monoamine oxidase A:Homo sapiens	56.218 % (7.308)	YES	6
30	Serine/threonine-protein kinase AKT:Homo sapiens	50.657 % (6.585)	YES	6
31	Human immunodeficiency virus type 1 integrase:Human immunodeficiency virus 1	50.603 % (6.578)	YES	5
32	Tyrosinase:Agaricus bisporus	50.508 % (6.566)	YES	6

1.5. Toxicity Prediction

Pred-hERG Results

Prediction / Potency	Confidence	Applicability domain (AD)	Probabilty Map
**Non-cardiotoxic (-)**	60%	Yes<br>(Value= 0.27 and limit = 0.26)	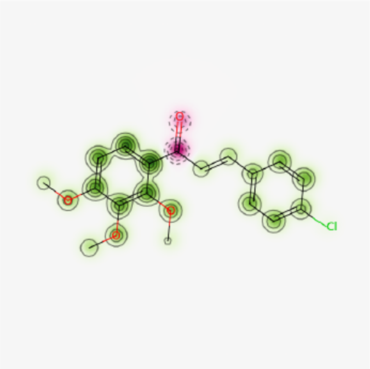
Not applicable	Not applicable		

Prediction for compound Molecule 1

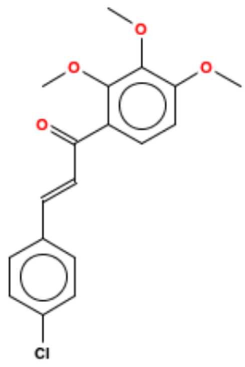

Prediction: Reliability:

Prediction is NON-Mutagenic, the result appears reliable. Anyhow, you should check it through the evaluation of the information given in the following sections.

Prediction for compound Molecule 1

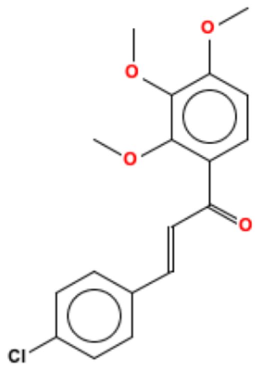

Prediction:

Prediction is NON-Mutagenic with a consensus score of 0.6, based on 4 models.

Prediction for compound Molecule 1

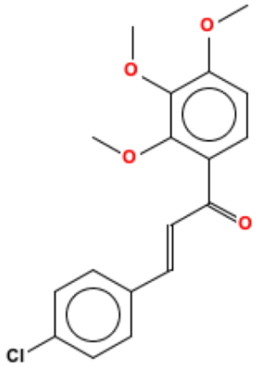

Prediction: Reliability:

Prediction is Mutagenic, but the result shows some critical aspects, which require to be checked:
- similar molecules found in the training set have experimental values that disagree with the predicted value
The following alerts have been found: SA38 Alkenylbenzenes

Prediction for compound Molecule 1

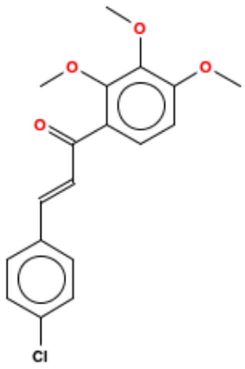

Prediction: Reliability:

Prediction is NON-Mutagen, but the result shows some critical aspects, which require to be checked:
- accuracy of prediction for similar molecules found in the training set is not optimal

Prediction for compound Molecule 1

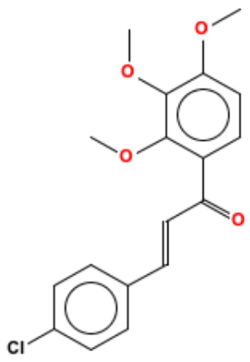

Prediction: Reliability:

Prediction is NON-Mutagenic, the result appears reliable. Anyhow, you should check it through the evaluation of the information given in the following sections.
The following relevant fragments have been found: SM172; SM191; SM193; SM200

Pred-hERG Results

Prediction / Potency	Confidence	Applicability domain (AD)	Probabilty Map
Potential cardiotoxic (+)	50%	Yes<br>(Value= 0.26 and limit = 0.26)	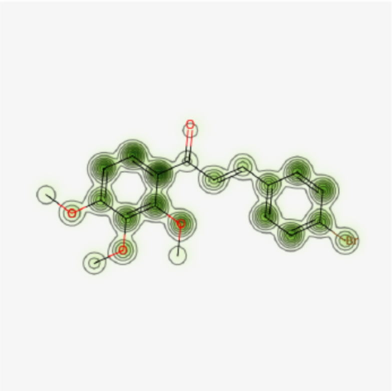
Weak or Moderate	60%		

Prediction for compound Molecule 1

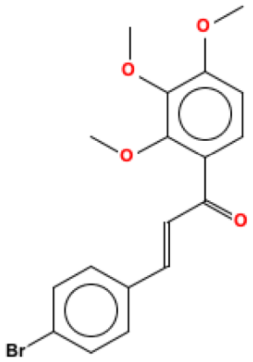

Prediction: Reliability:

Prediction is NON-Mutagenic, the result appears reliable. Anyhow, you should check it through the evaluation of the information given in the following sections.

Prediction for compound Molecule 1

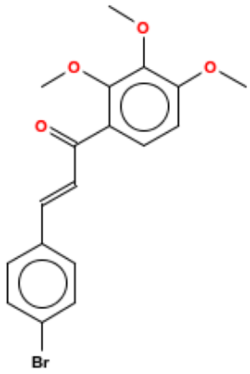

Prediction:

Prediction is NON-Mutagenic with a consensus score of 0.6, based on 4 models.

Prediction for compound Molecule 1

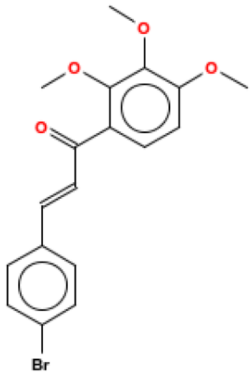

Prediction: Reliability:

Prediction is Mutagenic, but the result may be not reliable. A check of the information given in the following section should be done, paying particular attention to the following issues:
- similar molecules found in the training set have experimental values that disagree with the predicted value
- some atom centered fragments of the compound have not been found in the compounds of the training set or are rare fragments (2 infrequent fragments found)
The following alerts have been found: SA38 Alkenylbenzenes

Prediction for compound Molecule 1

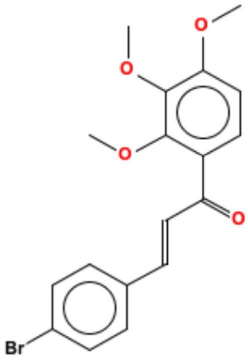

Prediction: Reliability:

Prediction is NON-Mutagen, but the result shows some critical aspects, which require to be checked:
- accuracy of prediction for similar molecules found in the training set is not optimal

Prediction for compound Molecule 1

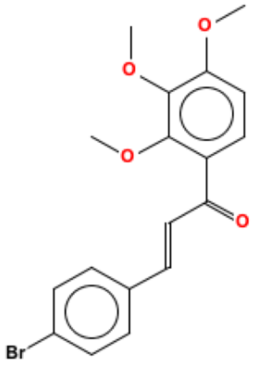

Prediction: ● Reliability: ★ ★ ★

Prediction is NON-Mutagenic, the result appears reliable. Anyhow, you should check it through the evaluation of the information given in the following sections.
The following relevant fragments have been found: SM152; SM172; SM191; SM200
